# Upcycling Water Pollutants Into Long‐Chain Polymers via Synergistic Interfacial Dechlorination and Organic Radical Stabilization

**DOI:** 10.1002/anie.3215238

**Published:** 2026-06-11

**Authors:** Ziwei Yao, Yidi Chen, Penghui Shao, Jian Liu, Xiaodan Wang, Kunsheng Hu, Xubiao Luo, Nanqi Ren, Xiaoguang Duan

**Affiliations:** ^1^ State Key Laboratory of Urban‐Rural Water Resources and Environment National Engineering Research Center for Safe Disposal and Resources Recovery of Sludge School of Ecology and Environment Harbin Institute of Technology Shenzhen Shenzhen People's Republic of China; ^2^ School of Chemical Engineering Adelaide University Adelaide South Australia Australia; ^3^ National‐Local Joint Engineering Research Center of Heavy Metals Pollutants Control and Resource Utilization Nanchang Hangkong University Nanchang People's Republic of China; ^4^ School of Environment and Energy Guangdong Provincial Key Laboratory of Solid Wastes Pollution Control and Recycling South China University of Technology Guangzhou China

**Keywords:** coal water remediation, electron transfer pathway, high‐entropy oxide composites, periodate, polymerization

## Abstract

High‐entropy oxides (HEOs) offer unusual electronic structures arising from lattice distortion, multimetal synergy, and high chemical stability. Here we report nitrogen‐doped carbon‐encapsulated HEOs catalysts (HEO@NC) synthesized by in situ carbothermal reduction for efficient pollutant removal and upcycling through selective oxidative polymerization. HEO@NC activates periodate via an electron‐transfer pathway coupled with surface‐adsorbed hydroxyl radicals, enabling the selective conversion of phenolic contaminants into polymeric products under extreme pH and strong ionic interference. Relative to metal‐free nitrogen‐doped carbon, HEO@NC markedly enhances periodate activation through interfacial electronic coupling and achieves a periodate utilization efficiency of 449.2%, far exceeding that of conventional mineralization. Density functional theory and experimental analyses reveal complementary roles of the HEOs components: Co/Ni provide periodate‐binding sites, Pt lowers the barrier for electron transport, Bi/Pb promote charge delocalization to stabilize polymeric intermediates, and oxygen orbitals strengthen periodate coordination and surface charge transfer via *p*–*d* hybridization. This synergy drives dechlorination‐coupled polymerization with sustained 4‐chlorophenol removal at ultralow oxidant consumption. HEO@NC further maintained > 95% efficiency with negligible metal leaching during 20‐day treatment of real coal chemical wastewater, demonstrating the potential for sustainable and low‐chemical‐consumption remediation of industrial‐relevant wastewater.

## Graphical abstract

HEO‐regulated electronic structure drives nonradical PI activation, promoting ETP‐mediated polymerization of 4CP into high‐molecular‐weight products with enhanced surface enrichment. Concurrent dechlorination occurs via radical coupling and ^•^OH_ads_ attack, enabling efficient carbon retention and selective product evolution at the liquid–solid interface.

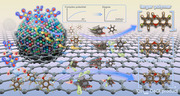

## 1 Introduction

Heterogeneous advanced oxidation processes (AOPs) based on periodate (PI) have attracted increasing attention owing to the high redox potential and versatility of PI‐derived reactive species (ROS). PI activation can proceed via either radical or nonradical pathways, enabling rapid degradation of diverse organic contaminants [1]. However, despite this promise, achieving efficient, controllable PI activation remains challenging, particularly in complex water matrices, where current strategies struggle to balance high reaction efficiency with long‐term system stability. Developing cost‐effective and robust catalysts is crucial for achieving efficient and sustainable water purification based on heterogeneous AOPs [2, 3]. However, many existing AOP catalysts suffer from critical limitations, including structural degradation and sluggish intra‐ and interfacial reaction kinetics toward target pollutants in the harsh and complex water environment [4, 5]. Engineering composite structures and interfacial properties of nanocatalysts has emerged as an effective strategy for boosting reaction kinetics and structural resilience, while simultaneously enabling the modulation of interfacial electronic structure to drive efficient oxidant activation [6, 7, 8]. Typical interface engineering methods include interfacial bridging and functionalization, phase engineering, vacancy creation, and the construction of heterogeneous interfaces [9, 10]. Nevertheless, the critical challenge of optimizing catalyst electronic structure remains, as it is essential for achieving efficient and controllable PI activation alongside long‐term operational stability under harsh conditions [11].

High‐entropy oxides (HEOs), composed of five or more principal metal elements in a well‐defined crystal structure, represent a promising class of materials with immense potential in catalysis, energy storage, and electrocatalysis [12, 13]. As accommodated in rock salt, perovskite, or spinel structures, the catalytic performance of HEOs is determined by the combination of surface energy, multimetal synergy, and lattice distortion, giving rise to tunable surface energy, flexible electronic structures, and intrinsic structural stability [14, 15, 16]. However, in AOP systems, nanoscale HEOs often suffer from sluggish interfacial electron transfer and metal leaching under acidic and strongly oxidative conditions, limiting their ability to regulate PI activation pathways in a controllable manner [17, 18, 19]. In this regard, constructing a protective armor via embedding the nanocatalyst under a carbon shell, such as coating with thickness‐controlled graphite or polydopamine, effectively secures structural stability in AOPs and ensures efficient, controllable PI activation [20, 21]. By in situ carbonizing and encapsulating metal nanoparticles within nitrogen‐doped graphite carbon shells, not only can the metal particles be anchored, but corrosion in heterogeneous catalysis can also be prevented [22]. Furthermore, the featured core‐shell structure will regulate interfacial charge redistribution, strengthens interfacial electronic coupling and transfer, and constructs spatially separated active sites to cooperatively drive multi‐reactant reactions, thereby enabling efficient and controllable PI activation and further enhancing catalytic stability and reaction controllability [23, 24, 25].

Herein, we report nitrogen‐doped carbon‐encapsulated high‐entropy oxide catalysts (HEO@NC) synthesized via in situ carbothermal reduction. HEO@NC effectively activates PI to drive oxidation of organic pollutants through an electron‐transfer pathway (ETP) and surface‐adsorbed hydroxyl radicals (^•^OH_ads_), outperforming nitrogen‐doped carbon (NC). The synergistic pathway enables selective removal of phenolic compounds over a wide pH range and in the presence of co‐existing ions. The HEO@NC‐PI system selectively polymerizes 4‐chlorophenol (4CP) into high‐molecular‐weight products. In the nanocomposites, HEOs optimize the electronic structures of interacting carbons, thereby enhancing the interfacial enrichment of target pollutants and polymeric intermediates. This selectivity originates from synergistic electronic interactions among the multiple metal species in the HEOs, which collectively tailor the density of states and optimize charge transfer at the carbon interface, thereby stabilizing surface‐bound organic radical intermediates to enable controlled polymer growth while simultaneously reducing PI consumption, achieving a PI utilization efficiency of up to 449.2%, corresponding to a 339.4% increase relative to the conventional mineralization pathway. Further density functional theory (DFT) and experimental analyses reveal insights into the individual roles of each metal species in HEOs, which collectively enhance the potential of activated PI complexes for sustained 4CP and intermediate polymerization. Notably, two dechlorination pathways are observed, including polymerization‐driven and radical‐based oxidative substitution. Conventional radical‐mediated dechlorination processes are primarily governed by oxidant potential and often suffer from nonselective attack, incomplete detoxification, and formation of hazardous intermediates (e.g., disinfection byproducts). In contrast, contaminant dechlorination is governed by intrinsic electronic properties (e.g., electronstatic potential and Fukui analysis), while ROS potential only affects oxidation efficiency. Fluorescence imaging using 7‐hydroxycoumarin confirms the formation of ^•^OH_ads_, which assist in dechlorination and the oxidative conversion of surface‐enriched species, offering a fundamentally important pathway for regulating product distribution and enhancing detoxification efficiency. To validate practical relevance, a 20‐day continuous‐flow test was conducted for treating simulated wastewater, and HEO@NC maintained > 95% removal efficiency and negligible metal leaching. This work showcases the promise of HEOs as robust and sustainable AOP catalysts for low‐chemical‐consumption and high‐efficiency wastewater treatment technologies featuring spontaneous pollutant upcycling and low emissions.

## 2 Results and Discussion

### 2.1 Synthesis and Characterization of HEO@NC

**FIGURE 1:**
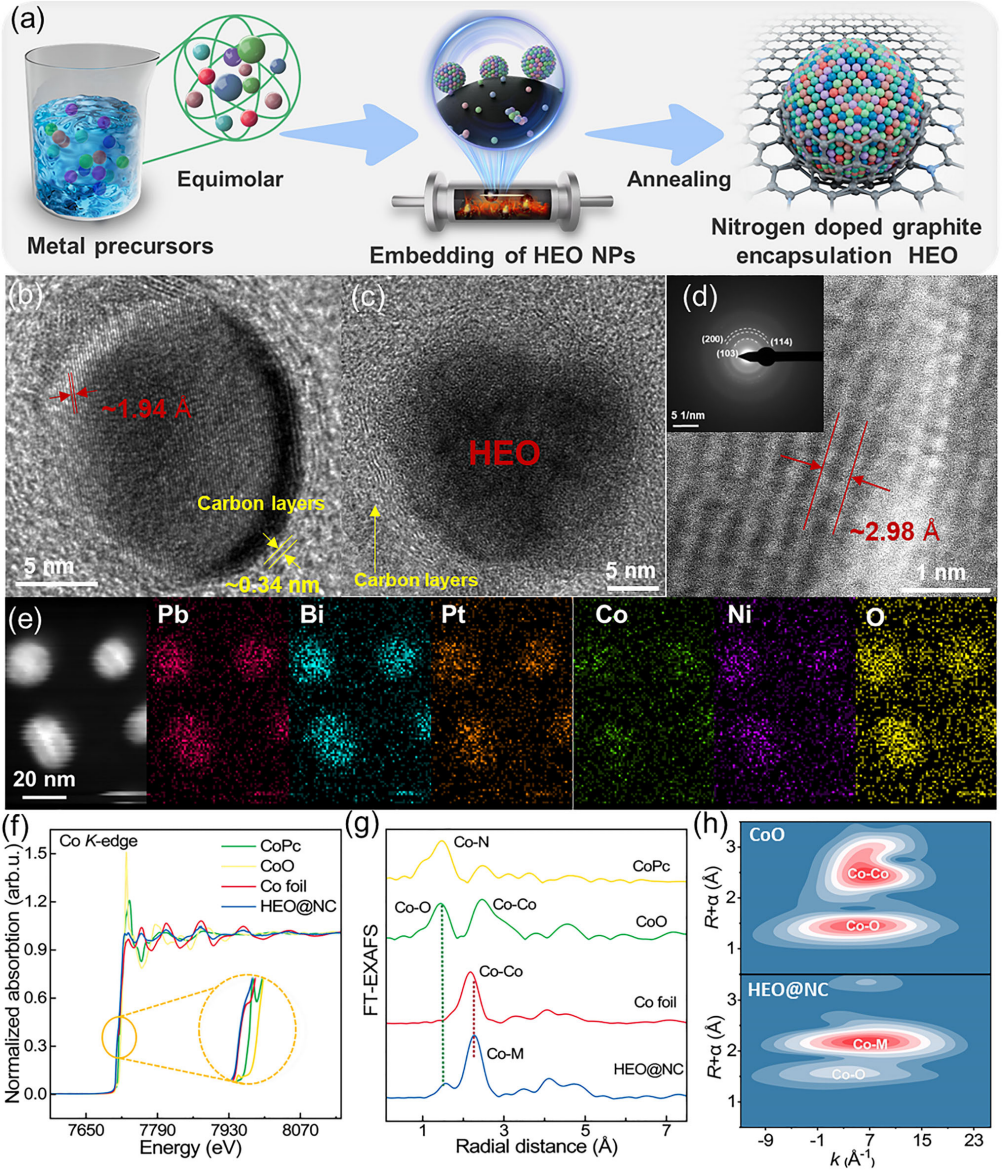
Synthesis and characterization of HEO@NC. (a) Synthesis process of HEO@NC and schematic diagram of the structure (b) TEM images of HEO@NC. (c) BF‐TEM images of HEO@NC reveal HEOs NPs encapsulated within carbon layers accompanied by defects. (d) HAADF‐STEM images and SAED pattern of HEO@NC. (e) Elemental mapping for HEO@NC. (f) XANES at Co L‐edge. (g) FT‐EXAFS spectra in R space. (h) WTs of EXAFS spectra of HEO@NC and CoO.

Figure 1a schematically illustrates the synthetic route, where a simple one‐pot oil‐phase synthesis involves heating five oxygen‐containing metal salt precursors (Pb, Bi, Pt, Co, and Ni) in air at 180°C for 5 h, followed by in situ encapsulation of the HEOs nanoparticles (NPs) through glucose‐assisted carbothermal reduction to obtain HEO@NC [26]. As depicted in the transmission electron microscopy (TEM) images (Figures S1 and S2), a significant number of nanoparticles, each with a diameter of approximately 12.5 ± 0.8 nm, are homogeneously distributed across the carbon surface. The powder x‐ray diffraction (XRD) pattern reveals diffraction peaks at 12.8°, 29.9°, 38.9°, 41.8°, and 46.5°, corresponding to the (002), (103), (006), (114), and (200) crystal planes of Pb_5_Bi_8_O_17_, respectively, which aligns with the XRD pattern of tetragonal Pb_5_Bi_8_O_17_ (PDF#53‐0652) (Figure S3). High‐resolution TEM (HRTEM) images reveal well‐defined lattice fringes with spacings of 1.94 Å, corresponding to the (200) plane of HEOs. The outer boundary region displays a layer spacing of 0.34 nm, corresponding to the graphitic carbon shells (Figure 1b and Figures S4–S6). Such self‐encapsulation with interfacial structural defects in the HEO@NC nanocomposites strengthens interfacial interactions and enhances structural stability of HEOs NPs under harsh reaction conditions, as evidenced by bright‐field TEM (BF‐TEM) imaging as shown in Figure 1c and Figure S6. The x‐ray diffraction results, indicating the successful synthesis of a single‐phase alloy oxide structure, which is further supported by the selected area electron diffraction (SAED) pattern, revealing well‐defined rings corresponding to the (103), (114), and (200) planes of the Pb_5_Bi_8_O_17_ phase (inset of Figure 1d). Additionally, the AC‐HAADF‐STEM image in Figure 1d, revealing a lattice spacing of 2.98 Å corresponding to the (103) facet, along with energy‐dispersive x‐ray spectroscopy (EDS) confirming the homogeneous distribution of the five metal elements and oxygen (Figure 1e and Figure S7), collectively verify the formation of HEOs. Moreover, BET analysis shows that the introduction of HEOs NPs did not significantly alter the surface area or pore size (Figure S8). The atomic ratio of metal elements in HEO@NC catalyst is determined to be Pb_1_Pt_1.2_Bi_1.5_Ni_0.3_Co_0.5_O_x_ by inductively coupled plasma‐optical emission spectrometry (ICP‐OES) (Table S1). These multiscale characterizations conclusively demonstrate that HEO@NC was successfully synthesized by the in situ glucose‐assisted carbothermal encapsulation strategy. The chemical composition and valence states of HEO@NC were characterized using x‐ray photoelectron spectroscopy (XPS). The wide scan spectrum indicates the presence of Pb, Bi, Pt, Co, Ni, and O elements in the synthesized HEO@NC, which is consistent with the EDS results (Figure S9) [27]. Compared to NC, the oxygen content has significantly increased, attributed to the loading of HEOs NPs (Table S2). The high‐resolution C 1s and N 1s spectra indicate that the embedded HEOs NPs in the carbon layers have minimal impact on carbon and nitrogen species. The high‐resolution spectra of Pt 4f, Pb 4f, Bi 4f, Co 2p, and Ni 2p show deconvoluted metal and oxidized states, indicating that all the metal elements primarily exist in their oxidation states (Figures S10 and S11).

However, electron spin resonance (ESR) analysis shows that polyenyl radicals generated during dehydrogenation convert into high‐density nitrogen defects with the addition of HEO particles, indicating that defect engineering could modulate electron transfer at the catalyst‐oxidant‐pollutant interface, thereby enhancing catalytic activity (Figure S12) [28]. The x‐ray absorption spectroscopy (XAS) was performed to investigate the chemical states and local coordination structures of the elements in HEO@NC. The x‐ray absorption near‐edge structure (XANES) results in Figure 1f show a higher white line intensity compared to the Co foil, indicating that the Co elements in HEO@NC are in an oxidized state, consistent with the XPS results [27, 29]. The Fourier‐transformed extended x‐ray absorption fine structure (FT‐EXAFS) of the Co K‐edge, shown in Figure 1g, is compared with those of standard CoO, Co foil, and CoPc. The R‐space spectrum reveals two dominant peaks: one at approximately 1.46 Å, corresponding to the Co‐O bond, and another at 2.19 Å, typically associated with Co‐Co interactions [30, 31]. This latter peak offset suggests the presence of Co‐M interactions (where M = Pb, Bi, Pt, Ni) within the HEO@NC, likely arising from mutual interactions among multiple elements within the high‐entropy framework. Besides, the wavelet transforms (WT) of the *k* ^3^‐weighted EXAFS spectra of HEO@NC also reveal two dominating peaks, maximum at ≈ 4–12 Å^−1^, ascribed to Co−O (1.46 Å) bonding and Co‐M (2.19 Å) interactions (Figure 1h). Strong Co−Co and Co−O coordination can be observed in the WT contour plots of Co foil and CoO respectively, which agrees with the results from EXAFS spectra (Figure S13).

### 2.2 Catalytic Performance of HEO@NC With PI Activation

**FIGURE 2:**
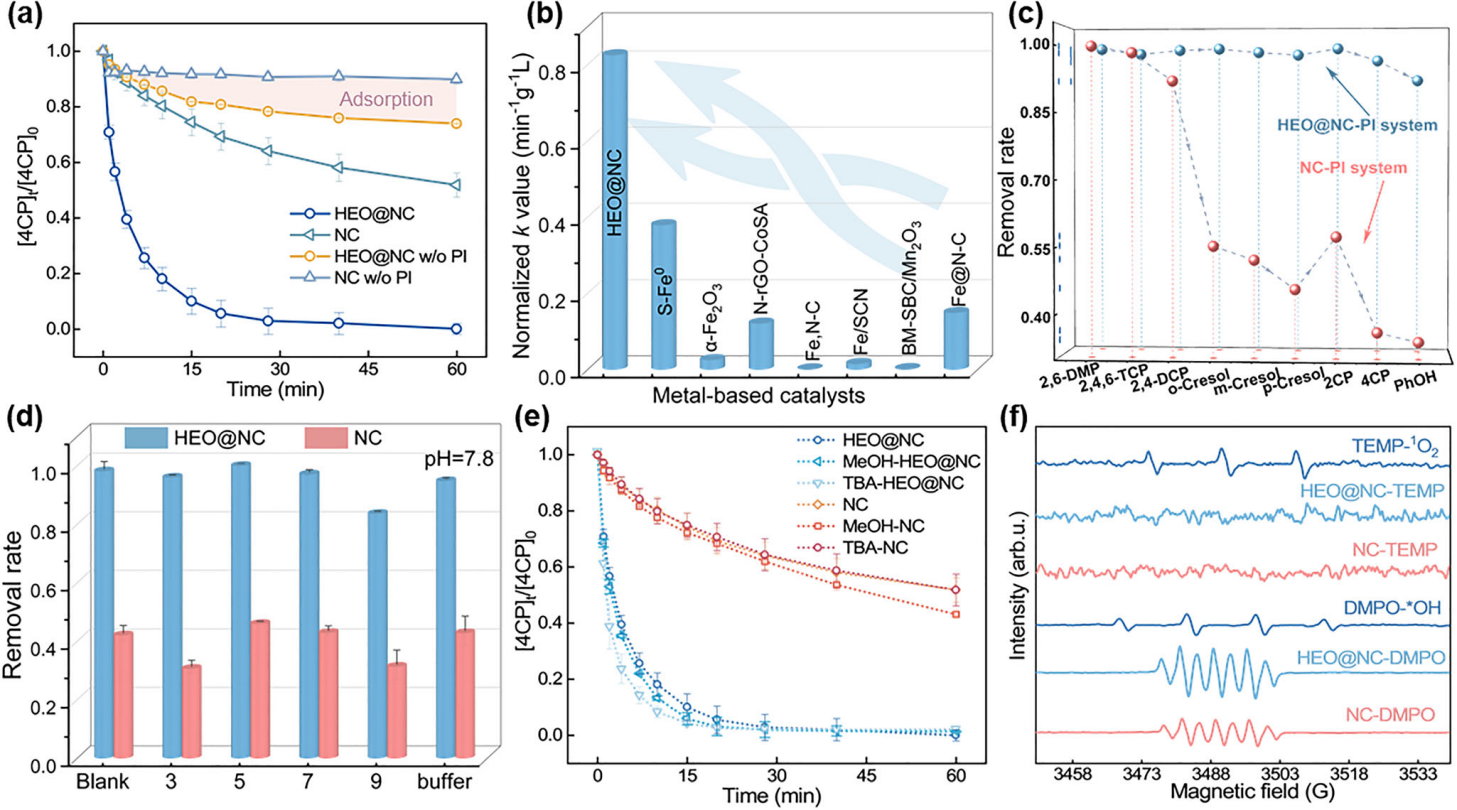
Catalytic performance of HEO@NC. (a) The removal rate against organic contaminants of HEO@NC and NC catalysts. (b) Recently reported performance comparisons of different catalysts with HEO@NC. (c) Evaluate the removal rates of different pollutants in the HEO@NC–PI system and the NC–PI system within 30 min. (d) HEO@NC and NC catalytic removal rate of 4CP under different pH and borate buffer environments. (e) Quenching effects by various scavengers. (f) ESR tests were conducted using DMPO and TEMP as trapping agents within the HEO@NC and NC system during PI activation. Dosage: PI (0.5 mM), scavengers (50 mM), reaction solution (50 mL), [pollutants]_0_ (0.1 mM), catalyst (0.1 g/L), reaction time (60 min).

The Fenton‐like activity of HEO@NC was evaluated using PI as the peroxide and 4CP as a model organic contaminant. As shown in Figure 2a, the catalyst‐alone systems exhibited minimal adsorption. NC/PI only attained less than 50% of 4CP removal in 60 min, while HEO@NC achieved complete 4CP degradation with PI within 25 min, with a degradation rate 18 times higher than NC (Figure S14). Thus, incorporating HEOs into NC greatly improved the catalytic activity for PI activation as well as the adsorption affinity toward organic substrates [32]. To mitigate the risk of secondary pollution from metal leaching in metal‐based catalysts, ICP analysis was conducted during heterogeneous Fenton‐like reactions, revealing that the release of Pb^2+^, Bi^2+^, Pt^2+^, Co^2+^, and Ni^2+^ from the HEO@NC catalyst was nearly negligible, and thus the metal leaching was significantly mitigated upon formation of the composite structure (Table S3). Furthermore, the HEO@NC catalyst exhibited exceptional activity, a superior normalized *k* value, and a high removal efficiency, underscoring its kinetic advantages and overall effectiveness in the system (Figure 2b and Table S4). Metal ions have demonstrated excellent catalytic performance in Fenton‐like reactions and are widely used for peroxide activation [33]. To investigate the superior performance of HEO@NC in removing 4CP, experiments were conducted using metal ions (Pb^2+^, Bi^2+^, Pt^2+^, Ni^2+^, and Co^2+^) as comparative catalysts with equimolar concentrations based on the metal content in HEO@NC, none of the metal ions exhibited removal performance (Figure S15 and Table S5). Similarly, introducing equivalent amounts of metallic Pb, Bi, and Ni NPs onto NC led to only moderate 4CP removal, whereas loading Pt and Co metals significantly improved 4CP oxidation efficiency to 93% and 98% within 60 min, indicating that Pt and Co are more effective for PI activation. These results further underscore the high intrinsic activity and low metal leaching of HEO@NC in AOPs.

Additionally, HEO@NC could effectively remove various substituted phenols. (Figure 2c and Figures S16 and S17). The substrate‐specific phenomenon was observed, where aromatic hydrocarbons with stronger electron‐donating groups, such as 2,4,6‐TCP, o‐cresol, and 2,6‐DMP, have lower oxidation potentials and are more easily oxidized by activated PI (Figure S18) [34]. To assess the stability and interference resistance of HEO@NC in practical water treatment applications, the decontamination performance of the HEO@NC–PI system was carefully evaluated across various pH conditions (Figure 2d). The pH range of 3.0–7.0 has minimal impact on the catalytic activity of HEO@NC, with reaction rates remaining unaffected when using borate buffer (Figure S19). Although slightly decreased activity is observed for HEO@NC at pH levels above 8.0, it is significantly less pronounced compared to NC, particularly under borate and strongly acidic conditions. This difference can be attributed to the pH‐induced shift in PI ionic species distribution (from IO_4_^−^ in an acidic environment to more stable H_2_I_2_O_10_^4−^ in an alkaline condition), which hinders activation and decomposition efficiency (Figure S20). In conclusion, the HEO@NC‐PI system significantly overcomes the limitations associated with a narrow pH range and exhibits superior performance compared to the NC‐PI system.

In traditional PI‐based AOPs, primary ROS like ^•^OH, O_2_^•−^, SO_4_^•−^, and IO_3_^•^ are considered potential active species [35, 36]. ROS often interact readily with various anions and pH levels, significantly impacting the degradation process [37]. Methanol (MeOH) and tert‐butyl alcohol (TBA) as quenchers for ^•^OH/SO_4_^•‒^ (9.7 × 10^8^ M/s, 2 × 10^7^ M/s) and ^•^OH (6.0 × 10^8^ M/s), respectively, to confirm their roles in 4CP oxidation. As shown in Figure 2e and Figure S21, both scavengers had minimal inhibitory impact on the HEO@NC‐PI and NC‐PI systems, excluding the generation of ^•^OH and SO_4_^•‒^. Similarly, using p‐benzoquinone as a quencher did not affect the removal efficiency, indicating that O_2_^•−^ is not involved in the process (Figure S22). Benzoic acid and nitro‐tetrazolium blue chloride (NBT) were subsequently used as probes to confirm the presence and contribution of ^•^OH and O_2_^•−^. The results showed minimal benzoic acid removal and no detectable production of monoformazan or diformazan, indicating that ^•^OH and O_2_^•−^ were not generated (Figures S23 and S24). Additionally, monitoring the consumption and transformation of PI reveals that the total concentration of IO_3_^−^ and IO_4_^−^ remains constant, indicating that no other iodine species are generated in the reaction system (Figure S25) [38]. This conclusion is further supported by experiments conducted at different PI concentrations (Figures S26 and S27). IO_3_^•^ is typically considered a potential active species in PI‐AOPs, while the direct evidence of its formation is still lacking [39]. Previous studies have shown that IO_3_^•^ is not capable of oxidizing 2,4,6‐trichlorophenol (2,4,6‐TCP), primarily due to its limited oxidative potential and the pronounced steric hindrance associated with the highly chlorinated structure of TCP [39]. As indicated in Figures S16 and S28, the HEO@NC‐PI and NC‐PI systems successfully removed 2,4,6‐TCP, implying that the primary reactive species was not IO_3_^•^.

Additionally, as depicted in Figure 2f, ESR did not detect any capture featured signals for ^•^OH, SO_4_^•‒^, and ^1^O_2_ in the presence of their specific spin trapping agents in either HEO@NC‐PI or NG‐PI systems [40]. However, a seven‐line spectrum corresponding to the oxidation product DMPOX was observed. The addition of MeOH and TBA had minimal impact on the DMPOX signal intensity, excluding the contribution of ^•^OH/SO_4_^•‒^ to DMPOX formation. This suggests that DMPOX is most likely generated through direct oxidation via ETP (Figure S29). Additionally, the addition of 2,2,6,6‐tetramethylpiperidine (TEMP) as a quencher for ^1^O_2_ had minimal impact on the decontamination performance of HEO@NC and NC systems, indicating that ^1^O_2_ made negligible contributions (Figure S30).

Moreover, experiments substituting deionized water with D_2_O did not accelerate the reaction kinetics, while using 9,10‐diphenylanthracene (DPA) as a selective ^1^O_2_ probe did not yield the featured DPAO_2_ products, both excluding the presence of ^1^O_2_ (Figures S31 and S32) in the HEO@NC‐PI and NC‐PI systems [41]. PMSO was used as a probe to clarify the role of high‐valence metal‐oxo (HVMO) species in the HEO@NC‐PI system, as HVMO can induce oxygen‐coupled two‐electron oxidation of PMSO to yield PMSO_2_, which is different from the products from radical oxidation [42]. As shown in Figures S33 and S34, PMSO did not convert to PMSO_2_, and DMSO, as the quenching agent for HVMO, showed no prohibitive effect, indicating that HVMO species are not generated in the HEO@NC‐PI system [33]. These findings collectively rule out the primary contributions of freely diffusing radicals (^•^OH, SO_4_^•‒^, O_2_^•‒^, IO_3_^•^), ^1^O_2_, and HVMO species, supporting the interfacial electron transfer mechanism as the main PI activation pathway in the HEO@NC‐PI system (Figure S35).

### 2.3 Insights Into the Mechanism of Electron Transfer

**FIGURE 3:**
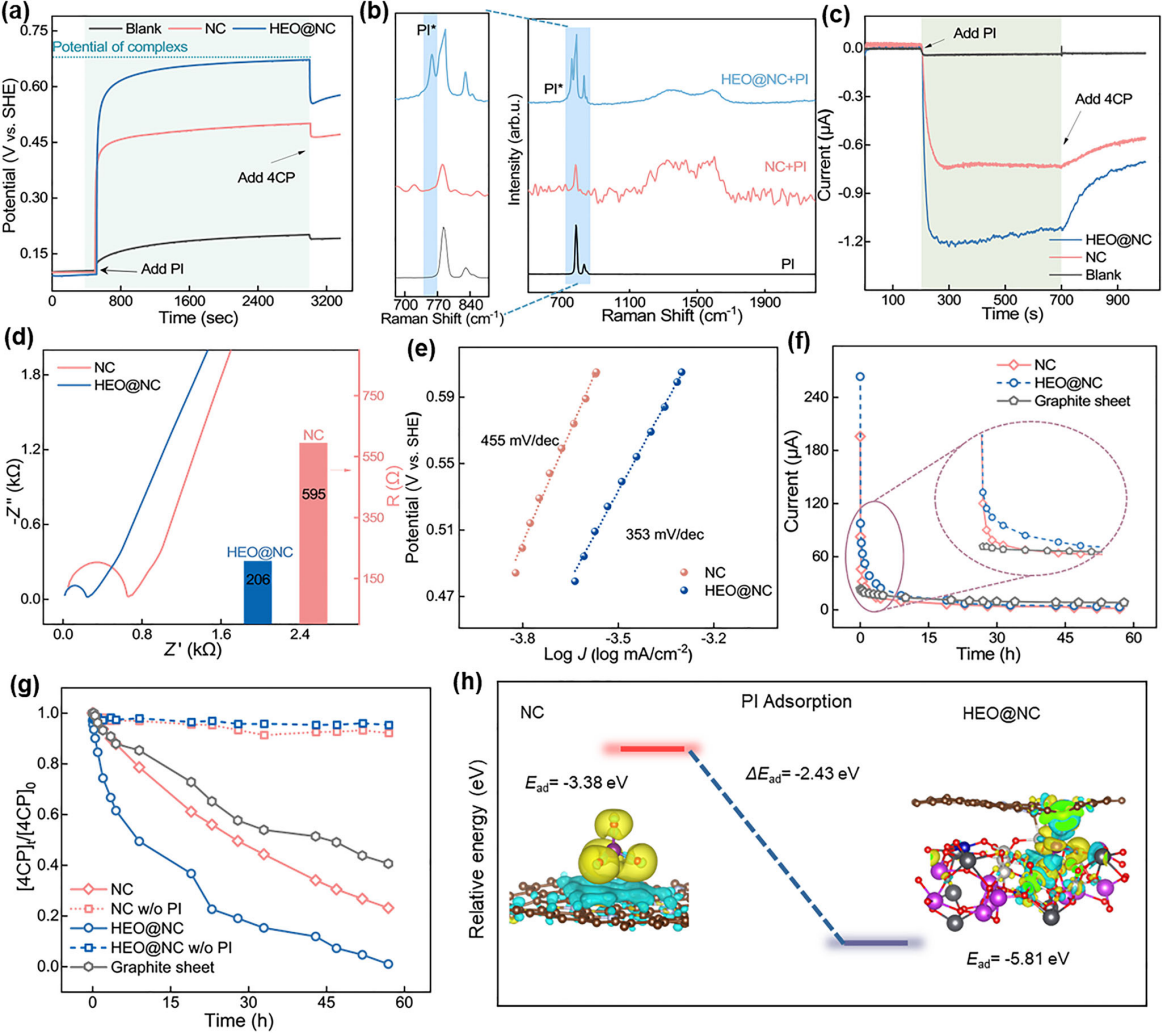
Identification of electronic synergy mechanism. (a) The open circuit potential on the HEO@NC−GCE and NC−GCE electrodes. (b) In situ Raman spectra of HEO@NC‐PI and NC‐PI in the liquid solution. (c) Chronoamperometry curve measurements on HEO@NC−GCE and NC−GCE electrodes. (d) Nyquist plots and fitting calculations of internal resistance for HEO@NC and NC. (e) Tafel slope on the HEO@NC and NC electrodes in 4CP solutions. (f) Current flows from the PI cell to the pollutants cell in a galvanic reactor. (g) Degradation rate of 4CP in the galvanic reactor anodic chamber. (h) The calculated electron density difference diagrams of HEO@NC and NC with PI adsorbed.

In order to figure out if the ETP existed in the HEO@NC‐PI and NC‐PI systems, various electrochemical techniques, such as OCPT, CA, EIS, LSV, and Tafel curve analyses, were conducted [37, 43]. As illustrated in Figure 3a, the injection of PI caused a rapid rise in OCPT for both HEO@NC‐GCE and NC‐GCE, indicating that the PI complex (PI*) formed on their surfaces. Notably, HEO@NC‐PI* showed a higher potential than NC‐PI*, suggesting a stronger oxidation capability [44]. With the addition of 4CP, a decrease in OCPT was observed, indicating that electron transfer occurred from 4CP to HEO@NC/NC‐PI*. Moreover, in situ Raman spectroscopy in Figure 3b directly evidenced the emergence of the PI* complex on HEO@NC and NC [45]. Further evidence from in situ synchrotron radiation‐based Fourier‐transform infrared (SR‐FTIR) analysis (Figure S36) reveals that in the HEO@NC and PI mixed suspension, the 1750 cm^−1^ stretching band gradually intensified over time. This observation indicates the formation of a PI* complex structure on the HEO@NC surface and confirms electron transfer between PI and HEO@NC. The proposition was also supported by the CA measurement (Figure 3c). The CV experiment showed that the current density for 4CP oxidation on HEO@NC was significantly higher than on NC, confirming the superior electron transfer capability of HEO@NC from 4CP to PI (Figure S37). The conductivity and electrochemical kinetics were further estimated using EIS and the *Tafel* analyses to elucidate the charge‐transfer characteristics and identify the rate‐limiting step. The equivalent circuit fittings indicated that the impedance of HEO@NC (206 Ω) was significantly lower than that of NC (595 Ω) due to the presence of HEOs NPs that promote interfacial electronic coupling and facilitate charge migration processes across the catalyst‐support interface (Figure 3d and Figure S38). By fitting LSV data to the Tafel curve, Tafel slopes for HEO@NC and NC were calculated as 353 and 455 mV/dec, respectively (Figure 3e and Figure S39). This difference indicates that HEO@NC achieves faster oxidation kinetics and more efficient electron transfer at elevated potentials. Galvanic reactor experiments were performed to analyze the mechanism of PI activation by HEO@NC/NC (Figure 3f and Figure S40). The current variations recorded during galvanic reactions revealed that HEO@NC generated a much higher current than NC, highlighting its superior 4CP oxidation kinetics and enhanced ETP activity [41]. In this process, 4CP undergoes proton‐coupled electron abstraction and oxidation, transferring electrons from the organic cell to the PI* complex on HEO@NC to fulfill the oxidation process (Figure 3g).

To elucidate the enhanced electron transfer mechanism of HEO@NC, we performed DFT calculations [21]. The density of states (DOS) analysis (Figure S41) reveals pronounced electronic states near the Fermi level (*E*_f_) in the Ni 3d, Co 3d, and Pt 5d orbitals, confirming their primary roles as electron donors. The Pt 5d orbitals, with their continuous distribution of unoccupied and partially occupied states, create a low‐energy‐barrier electron transport pathway that governs charge migration. In contrast, the d orbitals of Ni and Co contribute to surface adsorption through localized electron interactions. The Bi *d* and Pb *d* orbitals synergistically regulate the electronic structure by coupling with transition metal orbitals, enhancing charge delocalization and promoting charge transport among different metal species. The 2s and 2p orbitals of surface oxygen on HEOs exhibit distinct electron density peaks corresponding to their bonding roles: the 2s orbitals stabilize the HEOs structure, while the 2p orbitals strengthen the surface stability of HEOs and facilitate charge transfer via *p*–*d* hybridization with surrounding metal d orbitals. The XPS analysis presented in Figure S42 further validates this conclusion. The cooperative effects among multiple metals enhance catalytic activity by modulating the DOS distribution near the E_f_ (Ni/Co/Pt), promoting orbital hybridization (Bi/Pb), and optimizing oxygen bonding through O 2p–d interactions, collectively lowering the energy barriers for interfacial charge migration from the pollutant to the oxidant.

To quantify interfacial interactions, we systematically evaluated adsorption energies (*E*_ads_) between HEO@NC/NC and target molecules [46]. As shown in Figure 3h, the *E*_ads_ value for PI adsorption on the HEO@NC site (−5.81 eV) was substantially more negative than that on the NC site (−3.38 eV), indicating significantly stronger and more stable PI chemisorption upon the introduction of HEOs. Also, HEO@NC exhibited an improved chemisorption affinity for 4CP, further demonstrating its enhanced interaction with organic molecules (Figure S43 and Table S6). Further mechanistic insight emerges from electron density difference analysis; the charge transfer amount (*Q*_e_) from the active site to PI was determined using electron density difference data. The charge‐density‐difference analysis revealed that PI adsorption on HEO@NC induced more pronounced interfacial electron redistribution than on NC. The corresponding Bader charge transfer from HEO@NC to PI reached 1.53 e^−^, which is significantly higher than that of NC (0.93 e^−^), indicating its more efficient PI activation on HEO@NC (Figures S44–S45 and Table S7). Moreover, as shown in Figure S46, PDOS analysis reveals that the shift in orbital coupling from I 5p to 5s and enhanced interaction with O_(PI)_ atoms stabilize PI on the HEO@NC surface, promoting adsorption and charge transfer reactions. Additionally, the 3d orbitals of Ni, closer to the E_f_, interact more easily with the O atom of PI, further stabilizing the activated PI complex and enhancing pollutant oxidation [47].

### 2.4 Polymerization Dechlorination Mechanistic Investigation

To elucidate the mechanistic basis of the HEO@NC‐PI system's superior decontamination performance, multimodal analytical techniques were strategically employed [45]. Monitoring of the PI consumption rate indicates that the HEO@NC system exhibits significantly enhanced PI activation capacity and utilization efficiency, achieving a decomposition rate 8 times higher than that of NC (Figure S25). Moreover, the total organic carbon (TOC) removal analysis of HEO@NC‐PI and NC‐PI systems revealed a mismatch between theoretical (1.136 mM) and actual (0.253 mM) PI consumption equivalents during 4CP mineralization (Figure S47 and Table S8). This gap arises from the distinct electron demand of polymerization and mineralization pathways. Complete mineralization of 4CP to CO_2_ requires 26‐electron oxidation per 4CP molecule, corresponding to a theoretical PI:4CP ratio of 13:1 when IO_4_^−^ is reduced to IO_3_^−^ through a two‐electron process. In contrast, the polymerization pathway is initiated by single‐electron oxidation of 4CP to para‐chlorophenoxy radicals, where one PI molecule can accept electrons from two 4CP molecules, giving a theoretical PI:4CP ratio of 1:2. Subsequent radical coupling preserves the aromatic carbon skeleton within high‐molecular‐weight polymeric products rather than fully oxidizing it to CO_2_. Consequently, the HEO@NC‐PI system achieved a PI utilization efficiency of 449.2%, markedly reducing oxidant demand compared with conventional mineralization. Together with the proposed ETP mechanism, these results support a PI‐induced polymerization pathway that enables efficient 4CP removal under low‐stoichiometric oxidant consumption.

**FIGURE 4:**
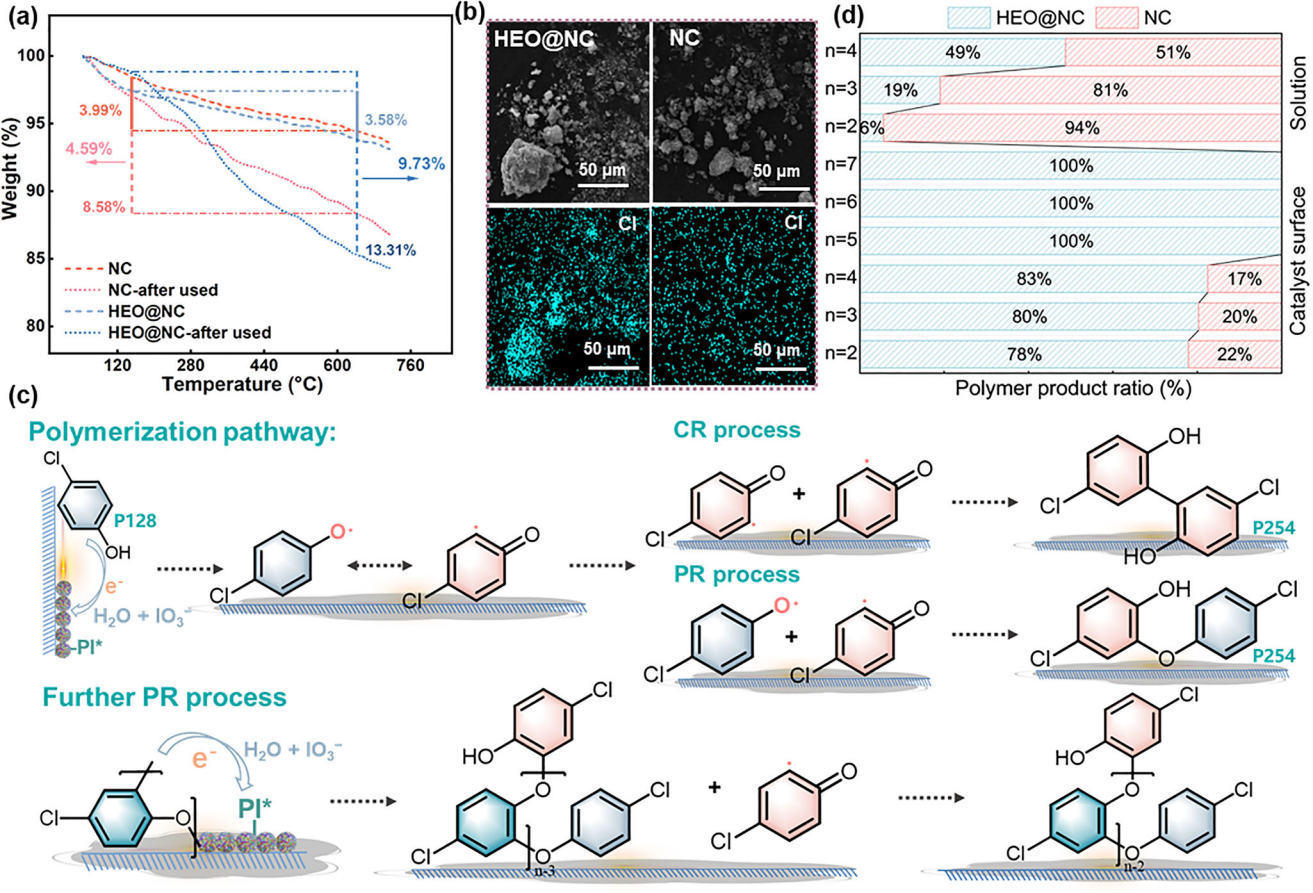
Characteristics of the polymerization mechanism. (a) TGA curves of HEO@NC and NC before and after the reaction. (b) Detected accumulated Cl elements in reacted HEO@NC and NC. (c) Proposed reaction pathways of 4CP oxidative coupling and polymerization on the catalyst surface. (d) Comparison of polymeric product distributions with different unit polymerization degrees in the NC‐PI and HEO@NC‐PI systems. Dosage: [4CP]_0_: 0.1 mM, PI: 0.5 mM, reaction solution: 50 mL, catalyst: 0.1 g/L.

Furthermore, thermogravimetric analysis (TGA) was performed to investigate the surface products of HEO@NC and NC. The TGA results indicated significantly greater decomposition of polymeric products for HEO@NC after AOP treatment, showing a mass loss of 13.31%, compared to 9.73% for the pristine HEO@NC and 4.59% for NC (Figure 4a). The results indicate that pollutants are not fully decomposed but instead accumulate on the catalyst surface through polymerization, with HEO@NC showing a stronger ability to drive this transformation [48]. As further verified in Figure 4b and Figure S48, energy‐dispersive spectroscopy (EDS) elemental mapping shows a notably stronger Cl signal on the surface of HEO@NC compared to NC. The same conclusion is also supported by the XPS spectra and BET analysis results (Figures S49–S51).

Previous studies suggest that FA can revert phenoxy radicals to their redox‐inactive phenol precursors, serving as a specific scavenger of phenoxy radicals, thus blocking the polymerization pathway [31, 49]. The results in Figures S52 and S53 indicate that both NC‐PI and HEO@NC‐PI are polymerization‐based systems. Compared to NC‐PI, the HEO@NC‐PI system exhibits faster polymerization kinetics, as reflected by the more pronounced inhibition effect with increasing FA concentration. Similarly, 0.05 mM FA fully inhibited 4CP removal in the NC‐PI system, whereas in the HEO@NC‐PI system, the inhibition increased with FA concentration, achieving complete suppression above 0.1 mM. These results confirm that FA effectively quenches para‐chlorophenoxy radicals and also highlight the faster polymerization kinetics of the HEO@NC‐PI system compared to NC‐PI.

To clarify the polymeric deposition mechanisms in the HEO@NC‐PI system, post‐reaction catalytic surfaces were subjected to product elution followed by ultrahigh performance liquid chromatography‐mass spectrometery (UPLC‐QTOF‐MS) characterization [31, 41]. This suggests that 4CP in the solution initially loses an electron from the PI* complex, triggering the electron‐coupled hydrogen atom abstraction (HAA) of 4CP and producing para‐chlorophenoxy radicals, which can stabilize through various resonance structures (Figure 4c and Table S9). In the radical chain reaction process, para‐chlorophenoxy radicals serve as key intermediates for the formation of oligomeric products. These dimers are most likely generated through C–C (tail‐to‐tail) coupling or C–O (head‐to‐tail) crosslinking of monomer units with the same m/z = 253 (Figure S54). Dimers and larger products may further undergo one electron and hydrogen loss to yield their corresponding organic radicals, leading to the formation of higher molecular weight polymers that deposit on the catalyst surface.

As evidenced by Figures S55–S68, comparative analysis of HEO@NC‐PI and NC‐PI systems reveals that higher‐degree polymers (m/z = 884, *n* = 7) were detected on HEO@NC, while only low‐molecular‐weight polymers (m/z = 506, *n* = 4) appeared on NC. Further analysis of the extracted and concentrated bulk solution from the HEO@NC‐PI and NC‐PI systems revealed the presence of dimer‐to‐tetramer products (*n* = 2–4) in the solution phase, indicating the release of low‐polymerization‐degree species. The analysis of polymerization degree distribution reveals that in the NC system (Figure 4d and Figure S69), most polymerized products migrate to the solution phase, whereas the HEO@NC system preferentially enriches high‐polymer products on the catalyst surface. This demonstrates that HEO incorporation optimizes the electronic structure of carbon planes by enhancing multimetal orbital hybridization, thereby promoting organic radical adsorption. Strong p–d interactions of O 2p and metal d orbitals stabilize the surface and enable efficient charge transfer, while the enrichment of polymeric intermediates further facilitates interfacial coupling and polymerization (Figure S41 and Table S6). To further support this mechanism, we performed thermodynamic free‐energy analyses of the polymerization pathways of 4CP molecules on the HEO@NC and NC surfaces. The results show that the surface‐mediated polymerization proceeds with relatively low activation barriers and is thermodynamically spontaneous, allowing the reaction to proceed under ambient conditions. Notably, compared with NC, the HEO@NC system exhibits lower free‐energy changes and reduced activation barriers in the key rate‐determining step, demonstrating the energetic advantage of HEO@NC for promoting the polymerization pathway (Figure S70). Concurrently, enhanced PI adsorption by the HEO@NC–PI system increases the PI complexation potential, thereby accelerating the kinetics of para‐chlorophenoxy radical generation. Moreover, strengthened 4CP and intermediate adsorption facilitate the continuous organic radical generation and conversion into high‐molecular‐weight polymeric products. This promotes surface enrichment of polymerization products, which critically enhances the system's pollutant removal efficiency.

**FIGURE 5:**
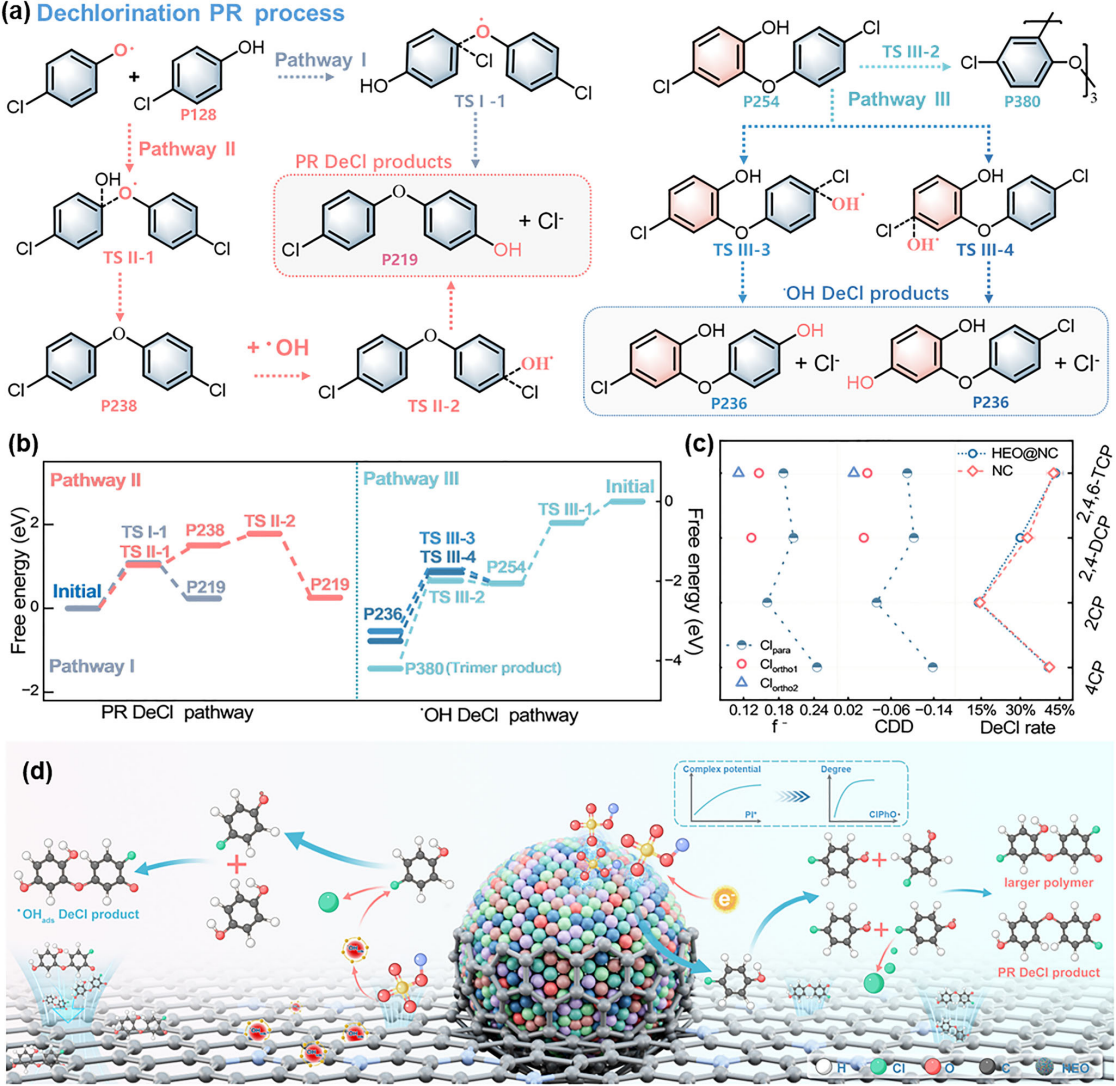
Mechanistic characteristics of polymerization dechlorination. (a) Proposed dechlorination pathways involved in 4CP polymerization on the catalyst surface. (b) Reaction energy barriers of the different dechlorination pathways. (c) f^−^ and CDD of different chlorophenols with their dechlorination degrees. (d) Schematic diagram of the polymerization dechlorination mechanism. Dosage: [4CP]_0_: 0.1 mM, PI: 0.5 mM, reaction solution: 50 mL, catalyst: 0.1 g/L.

Notably, product analysis revealed partial dechlorination products were identified, with ion chromatography detecting Cl^−^ in both NC‐PI and HEO@NC‐PI systems (Figure S71). Methanol quenching experiments revealed no impact on dechlorination efficiency, ruling out the involvement of reductive species (e.g., atomic hydrogen), suggesting that the observed dehydrochlorination and hydroxyl‐mediated dechlorination pathways, as shown in Figure 5a, may originate from polymerization dechlorination and ^•^OH_ads_‐induced hydroxylation processes, respectively [50]. The presence of surface‐confined ^•^OH_ads_ was further confirmed by coumarin probe experiments combined with fluorescence microscopy imaging (Figures S72 and S73). Unlike freely diffusing _•_OH in the bulk solution, ^•^OH_ads_ is generated and stabilized at the catalyst interface, where its limited mobility and short lifetime make it difficult to detect or quench using conventional homogeneous radical probes. The observed fluorescence signal from 7‐hydroxycoumarin formation provides direct evidence for interfacial ^•^OH_ads_ species distinct from bulk ^•^OH. As shown in Figure 5b and Figures S74 and S75, quantum chemical calculations further confirm the thermodynamic feasibility of the proposed dechlorination pathways. The comparable reaction free energies allow the coexistence of partially chlorinated intermediates and dechlorinated polymeric products. UPLC–QTOF–MS analysis was used here only for semiquantitative comparison of relative signal intensities of detected oligomeric products, rather than for absolute quantification. The results show that, compared with NC, the HEO@NC system favors the formation and surface enrichment of higher‐polymerization‐degree products, with only a minor fraction detected in the solution phase (Figures S76 and S77). In contrast, the NC system exhibited low 4CP removal efficiency alongside substantial diffusion of chlorinated polymeric products into the solution phase, consistent with aforementioned findings. This distribution pattern confirms that the incorporation of HEOs optimizes the electronic structure of the catalyst, thereby enhancing adsorption and surface enrichment of polymeric intermediates. Such targeted accumulation not only facilitates efficient dechlorination and generation of high‐molecular‐weight products but also minimizes the release of hazardous chlorinated byproducts into the aqueous phase. Notably, increasing the reaction temperature suppressed the surface enrichment of polymeric products and promoted their diffusion into the bulk solution, further underscoring the critical role of interfacial enrichment in governing polymer chain growth and reaction selectivity (Figures S78–S87). Furthermore, the proportion of dechlorination products was found to be largely consistent between the NC and HEO@NC systems. This indicates that the dechlorination behavior is not directly related to the high potential of the PI* complex but is primarily determined by the intrinsic electronic properties of the contaminants themselves. Building on this insight, we further conducted a comparative analysis of the dechlorination behaviors across different chlorophenolic compounds.

As shown in Figure S88, chlorophenols with different chlorine substitution positions and numbers exhibited distinct dechlorination proportions. For each chlorophenol, the final dechlorination proportion remained nearly identical in the NC‐PI and HEO@NC‐PI systems, indicating that the dechlorination selectivity is primarily governed by the intrinsic molecular structure of the pollutant. In contrast, HEO@NC accelerated chlorophenol removal and Cl^−^ release through a more efficient electron‐transfer pathway, without changing the final dechlorination trend. These findings further support the aforementioned conclusion that dechlorination behavior is governed solely by the intrinsic electronic properties of the contaminant itself. Ionization potential (IP), a critical indicator of the redox capacity, governs the electron‐donating ability and consequent oxidation behavior of contaminants with distinct IP values [34]. Analysis of the IP values of four chlorophenols revealed that the higher dechlorination efficiency of 4CP is closely related to its higher IP value. The ^•^OH_ads_ tend to oxidize contaminants with higher IP values, which coincides well with the experimental observations (Figure S89) [51]. Furthermore, electrostatic potential (ESP) mapping on the van der Waals surfaces of various chlorophenols (Figure S90) reveals that negative ESP regions correspond to electron‐rich sites, while positive ESP values denote electron‐deficient zones. Critical point analysis of the ESP distribution further confirms preferential formation of para‐chlorophenoxy radicals, thus providing strong evidence for polymerization‐induced dechlorination.

To further quantitatively elucidate the reactivity of individual atoms and the intrinsic differences in active sites and polymerization behaviors among various chlorophenols, we calculated the electrophilic Fukui index (f^−^) representing susceptibility to electrophilic attack, along with condensed dual descriptors (CDD) and local electrophilicity/nucleophilicity indices (Figures S91–S94 and Tables S10–S13). The results reveal that the para‐chlorine atom possesses the highest f^−^ and CDD values, suggesting its high reactivity toward electrophilic attack by ^•^OH_ads_ and organic radicals. Within the benzene ring, the hydroxyl group (–OH) functions as an electron‐donating substituent, increasing electron density at the para‐position and thereby facilitating ^•^OH_ads_‐induced chlorine substitution. As shown in Figure 5c, this electronic modulation accounts for the experimentally observed dechlorination trend: 2CP < DCP < TCP ≈ 4CP. Consistently, when peroxydisulfate (PDS) was used as an alternative oxidant (Figure S95), the dechlorination efficiencies of different chlorophenols followed the same order. This result further supports that the dechlorination behavior is primarily governed by the intrinsic electronic properties of the pollutant molecules rather than by the specific oxidant. Notably, the similar dechlorination efficiency in both NC and HEO@NC systems reveals that ^•^OH_ads_ primarily originates from surface activation of the carbon support, thereby initiating oxidative substitution‐based dechlorination of surface‐enriched chlorinated pollutants and polymeric intermediates. Such dechlorination pathways significantly enhance pollutant removal efficiency and substantially reduce toxicity via eliminating disinfection/chlorinated byproducts, which is also beneficial for treating organic wastewater containing high salinity.

Based on the aforementioned results, we provided a molecular‐level elucidation of the polymerization–coupled dechlorination effect (Figure 5d). HEOs regulate the electronic structure of the heterostructure and construct spatially separated but electronically coupled interfacial sites. PI is preferentially coordinated and activated at HEO‐regulated metal/oxygen sites, whereas 4CP and its polymeric intermediates are mainly enriched on the N‐doped carbon shell. This cooperative interface promotes nonradical electron transfer from adsorbed 4CP to the activated PI* complex, thereby driving selective polymerization into high‐molecular‐weight products while improving PI utilization efficiency. This adsorption‐enhanced surface enrichment promotes polymerization and regulates product distribution at the liquid–solid interface. The dechlorination behavior during polymerization is governed by the intrinsic electronic properties of the pollutants. Besides dechlorination through direct radical coupling, surface‐confined ^•^OH_ads_ provides an additional interfacial oxidative pathway for C‐Cl bond cleavage, with preferential attack at the para‐position. Thus, ETP‐driven polymerization and ^•^OH_ads_‐assisted dechlorination co‐exist at the catalyst surface, jointly enabling dechlorination‐coupled polymer growth.

### 2.5 Biotoxicity Risks and Practical Application Stability Test

**FIGURE 6:**
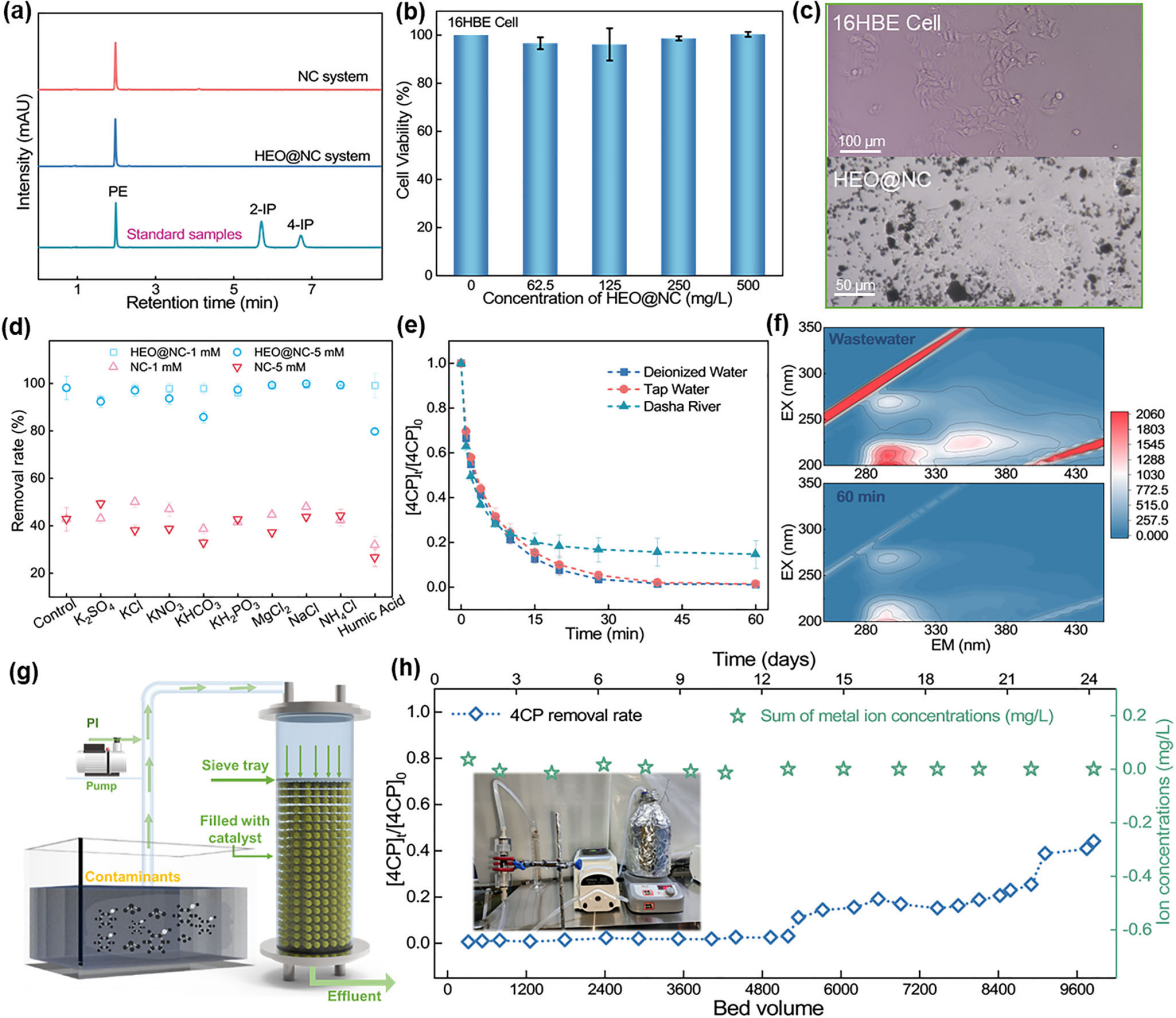
Evaluation of the adaptability and long‐term stability of HEO@NC. (a) Detection of HOI generated in HEO@NC‐PI system using phenol as the trapping agent. (b) Cell viability of 16HBE under different concentrations of HEO@NC. Cell culture time: 48 h. (c) Cells in the culture medium were observed under a microscope. (d) HEO@NC and NC catalytic degradation rates of 4CP with the interference of 1 and 5 mM salts and 1 and 5 mg/L humic acid. (e) Treatment of 4CP in real water matrices. (f) EEM spectra of collected coal chemical industry wastewater before and after treatment by HEO@NC–PI system. (g) Schematic of the fixed‐bed column continuous flow system. (h) The efficiency of 4CP removal and the metal leakage of HEO@NC in a continuous flow system.

In PI‐AOP systems, the generation of reactive iodine species like I_2_, I_3_^−^, and HOI may raise concerns about the formation of iodinated byproducts, while IO_3_^−^ is regarded as a safe and non‐toxic iodine species, even suitable for use as a food additive. Therefore, the products from PI decomposition were analyzed. As shown in Figure 6a and Figure S96, with phenol as a trapping agent in the HEO@NC‐PI system, 4‐iodophenol (4‐IP) and 2‐iodophenol (2‐IP) were not detected, indicating that HOI was not formed. Also, UPLC‐QTOF‐MS (Figure S97) reveals negligible formation of iodinated byproducts in the solution of the HEO@NC‐PI system. Additionally, the starch colorimetric test (Figure S98) did not indicate the presence of I_2_ or I_3_^−^ in the system [52]. Further investigation was conducted on the transformation pathway of IO_4_^−^. As shown in Figure S25, the yield of IO_3_^−^ was approximately 100% (the molar ratio of IO_3_^−^ produced to IO_4_^−^ consumed), indicating that almost all IO_4_^−^ was converted into IO_3_^−^, implying that IO_3_^−^ does not participate in further reactions with HEO@NC (Figure S99) [35].

We conducted in vitro cytotoxicity tests on human bronchial epithelial cells (16HBE) to examine their biotoxicity. As shown in Figure 6b, the CTL assay results indicated that varying concentrations of HEO@NC exhibited minimal cytotoxicity toward 16HBE cells. In contrast, Co^2+^ used as a control group demonstrated significant dose‐dependent toxicity (Figure S100). Microscopic observations revealed that HEO@NC‐treated cells were well‐spread with intact membranes and high confluency, whereas metal ion‐treated cells appeared shrunken with damaged membranes and reduced numbers, thereby underscoring the superior biocompatibility of HEO@NC (Figure 6c and Figure S101). Moreover, the potential biological impact of trace polymeric products remaining in treated water was evaluated using zebrafish embryo assays (Figure S102). Effluents treated by the HEO@NC system showed no adverse effects on embryo survival, hatching, or morphology, indicating negligible toxicity of residual soluble products. In contrast, effluents from the NC system induce pronounced malformations, likely due to residual 4CP and partially oxidized intermediates. These results highlight the critical role of polymerization‐driven micropollutant enrichment in mitigating ecological risk. Furthermore, the impacts of the co‐existing dissolved organic matter (DOM, represented by humic acid) and typical anion species (e.g., SO_4_^2−^, Cl^−^, NO_3_^−^, HCO_3_^−^, and H_2_PO_4_^−^) on 4CP removal by HEO@NC and NC were further investigated [53]. As shown in Figure 6d, the presence of representative anions and cations, including Cl^−^, SO_4_^2−^, NO_3_^−^, Na^+^, Mg^2+^, and NH_4_^+^, caused negligible inhibition of 4CP removal at both 1 and 5 mM concentrations, with detailed results shown in Figure S103. These findings demonstrate the strong tolerance of the HEO@NC–PI system toward common inorganic ions in realistic water matrices.

UPLC‐QTOF‐MS analysis was further used to evaluate the effects of pH and representative co‐existing ions on the oligomeric product distribution. Because authentic standards for oligomeric products are unavailable, the detected MS signal intensities were used only for semiquantitative comparison of relative product distributions rather than absolute polymer yields. As shown in Figure S104, co‐existing Cl^−^ ions caused only minor changes in the detected oligomer profiles, indicating that the polymerization pathway is tolerant to chloride interference. In contrast, the initial pH exerted a more pronounced influence on polymerization behavior. Acidic conditions affected polymerization initiation and propagation by modulating proton‐coupled electron transfer and subsequent phenoxy radical formation, whereas alkaline conditions altered PI speciation and activation efficiency, thereby influencing the degree of polymerization and final product distribution.

To evaluate the practicality of the HEO@NC‐PI system, secondary wastewater from the coal chemical industry was tested. In experiments with real water matrices, the HEO@NC‐PI system showed robust resistance to interference, even in more complex water conditions (Figure 6e). As shown in Figure 6f and Table S14, the excitation‐emission matrix (EEM) spectra revealed that soluble microbial byproduct‐like substances and phenolic humic acid‐like compounds in the coal‐chemical wastewater were effectively removed, as evidenced by the marked decrease in fluorescence intensity at the corresponding peaks (Figure S105). In contrast, long‐term continuous‐flow experiments offered a more thorough assessment of the catalyst efficiency in a fixed‐bed reactor for the degradation of organic pollutants (Figure 6g and Figure S106) [54]. Results from the 25‐day 4CP degradation test showed that the HEO@NC‐PI system maintained its high degradation efficiency for a total of 5000 bed volumes (Figure 6h). Furthermore, the metal ion leaching was negligible (Table S15), confirming the exceptional structural stability of HEO@NC under prolonged polymerization conditions [55]. Moreover, the catalytic activity could be effectively restored by moderate thermal annealing, indicating that surface deactivation is largely reversible and mainly arises from removable organic fouling rather than irreversible structural damage (Figure S107). Complementary TG and FTIR analyses further confirmed the accumulation of thermally stable polymeric products on the used catalyst, highlighting both the durability and regenerability of HEO@NC under long‐term operation (Figure S108).

## 3 Conclusion

This work successfully synthesized nitrogen‐doped carbon‐encapsulated high‐entropy oxide catalysts (HEO@NC) via in situ carbothermal synthesis and applied them for the first time in the elimination of pollutants using PI‐activated Fenton‐like AOPs. The unique electronic structure of HEO@NC enables superior PI activation efficiency compared to NC, driving a coupled mechanism of ETP and ^•^OH_ads_ that effectively removes phenolic pollutants across a wide pH‐operating window with strong resistance to ionic interference. Notably, the HEO@NC‐PI system facilitates selective polymerization of 4CP into high‐molecular‐weight products, with enhanced adsorption‐driven enrichment of intermediates, resulting in unprecedented PI utilization efficiency (449.2%), corresponding to a 339.4% increase relative to the conventional mineralization pathway. Synergistic effects of multimetal components were revealed, including Co/Ni as PI coupling sites, Pt for low‐barrier electron transfer, Bi/Pb for charge delocalization, and oxygen for stabilization of radical intermediate and surface enrichment. Experimental results reveal dual dechlorination mechanisms: polymerization‐based dechlorination and surface radical‐mediated dechlorination. Dechlorination pathways are governed by intrinsic electronic properties of pollutants for both NC and HEO@NC, while oxidant potentials influence the overall oxidation kinetics. Practical validation of HEO@NC in coal chemical wastewater treatment confirmed its long‐term stability (> 95% efficiency over 20 days) and negligible metal leaching. This work pioneers HEO@NC as innovative catalysts for catalytic wastewater remediation, featured for their minimal oxidant demand, pollutant upcycling, water detoxication, and reduced carbon footprint.

## Author Contributions

**Ziwei Yao**: conceptualization, investigation, data curation, formal analysis, writing – original draft. **Yidi Chen**: conceptualization, funding acquisition, supervision, formal analysis, project administration, writing – review and editing. **Penghui Shao**: formal analysis, supervision. **Jian Liu**: investigation. **Xiaodan Wang**: formal analysis. **Kunsheng Hu**: formal analysis, investigation. **Xubiao Luo**: writing – review and editing, formal analysis. **Nanqi Ren**: writing – review and editing, formal analysis. **Xiaoguang Duan**: conceptualization, investigation, supervision, writing – review and editing, resources, formal analysis.

## Conflicts of Interest

The authors declare no conflicts of interest.

## Supporting information




**Supporting File 1**: anie73021‐sup‐0001‐SuppMat.docx.

## Data Availability

The data that support the findings of this study are available from the corresponding author upon reasonable request.
