# Supplementary material for: Upcycling Water Pollutants Into Long‐Chain Polymers via Synergistic Interfacial Dechlorination and Organic Radical Stabilization
Source: Angew Chem Int Ed Engl. 2026 Jun 11;65(32):e3215238. doi: 10.1002/anie.3215238 (PMC13427219; doi:10.1002/anie.3215238)
Supplement: Supplementary file 1 — Supporting File 1: anie73021‐sup‐0001‐SuppMat.docx. [file ANIE-65-e3215238-s001.docx]

*Supporting Information*

***Upcycling Water Pollutants into Long-Chain Polymers via Synergistic Interfacial Dechlorination and Organic Radical Stabilization***

*Ziwei Yao^a^, Yidi Chen^a,b^ *, Penghui Shao^c^, Jian Liu^d^, Xiaodan Wang^a^, Kunsheng Hu^b^, Xubiao Luo^c^, Nanqi Ren^a^, Xiaoguang Duan^b^**

*^a^* *State Key Laboratory of Urban-rural Water Resources and Environment, National Engineering Research Center for Safe Disposal and Resources Recovery of Sludge, School of Ecology and Environment, Harbin Institute of Technology, Shenzhen, Shenzhen, 518055, P. R. China*

*^b^ School of Chemical Engineering, Adelaide University, Adelaide, SA 5005, Australia*

*^c^ National-Local Joint Engineering Research Center of Heavy Metals Pollutants Control and Resource Utilization, Nanchang Hangkong University, Nanchang 330063, P. R. China*

*^d^ School of Environment and Energy, Guangdong Provincial Key Laboratory of Solid Wastes Pollution Control and Recycling, South China University of Technology, Guangzhou, Guangdong 510006, China*

*Yidi Chen*, Xiaoguang Duan**

**To whom correspondence may be addressed.*

***Email:*** *chenyidi@hit.edu.cn, xiaoguang.duan@ adelaide.edu.au*

**This PDF file includes:**

Supporting Methods

Supporting Figures 1 to 108

Supporting Table 1 to 15

Supporting References

**Supporting** **Methods**

**Reagents and materials.** Bismuth subcarbonate ((BiO)_2_CO_3_), 90%), lead(II) acetylacetonate (Pb(acac)_2_, 95%), nickel(II) carbonate basic (NiCO_3_·2Ni(OH)_2_, 98%), cobalt(II) carbonate basic (2CoCO_3_·3Co(OH)_2_, 98%) and 9,10-Diphenylanthracene (DPA, 98%) were purchased from Aladdin. Oleylamine (OAm, >70%) was obtained from Sigma-Aldrich. ethanol (99.5%) and sodium periodate (NaIO_4_, 99.8%) were purchased from Macklin. Ascorbic acid (AA, 99%) was purchased from J&K Scientific. Platinum(II) acetylacetonate (Pt(acac)_2_, 98%), glucose (98%),1-Octadecene(ODE, 98%), Urea(CH_4_N_2_O, 99%) were supplied by Bidepharm (Shanghai, China). 2,2,6,6-Tetramethylpiperidine (TEMP, 98%) and 5,5-Dimethyl-1-pyrroline N-oxide (DMPO, 97%) were sourced from Dojindo Laboratories (Kumamoto, Japan). Other reagents not specified were purchased from Aladdin Chemical Reagent Company. Water samples from local bodies (tap water and Dasha River) in Shenzhen, Guangdong Province, were collected separately. Unless indicated otherwise for the actual water matrix test, all experiments were conducted using deionized water.

**Catalyst Characterization.** The TEM and HRTEM were tested by using a Thermo Scientific Talos F200X G2 microscope at 200 kV. High-angle annular dark-field scanning transmission electron microscopy (HAADF-STEM) using a JEOL JEM-ARM200F was employed to investigate the morphology and structure of the catalysts. XPS analysis was performed on a Thermo Scientific K-Alpha spectrometer with monochromatic Al-Kα radiation (1486.6 eV), using the C 1s peak at 284.8 eV for charge calibration. To ensure detectable metal content, the metal amount in the characterization samples was increased tenfold without changing other conditions. The crystal phases of the catalysts were characterized by XRD patterns with CuK-α radiation (SmartLab 3kw, Rigaku Co.). FTIR spectroscopy was obtained on a Nicolet 6700 Thermofisher in a range of 400−4000 cm^−1^. The ESR measurements employing 2,2,6,6-Tetramethyl-4-piperidone hydrochloride (TEMP) and 5,5-dimethyl-1-pyrrolineN-oxide (DMPO) for radical detection were conducted on an ESR5000 spectrometer (Bruker Co.). ICP-OES experiments were performed using an Avio 550 Max (Perkin Elmer Co.) to determine the content of metals in catalysts and leakage of metal ions during degradation. The rate of 4CP mineralization was assessed using a total organic carbon (TOC) analyzer (Multi N/C 2100 S, Analytik Jena), while the polymerization products were identified through UPLC-QTOF-MS (Waters Xevo G2-XS QTOF). XAS measurements encompassing metal K-edge XANES and EXAFS spectra were conducted in fluorescence mode at the Australian Synchrotron. All spectra were collected under ambient conditions and processed using standard protocols, with energy calibration, background correction, and edge-step normalization performed through the ATHENA module of the IFEFFIT software package.

**Preparation of Catalysts.** HEO@NC was synthesized via a wet-chemical method followed by high-temperature annealing. Briefly, Pt(acac)_2_, (BiO)_2_CO_3_, Pb(acac)_2_, NiCO_3_·2Ni(OH)_2_, and 2CoCO_3_·3Co(OH)_2_ were dispersed in a mixed solvent of OAm and ODE, with AA serving as the reducing agent. The mixture was sonicated and subsequently heated at 180 °C for 5 h under ambient air. After cooling, the obtained product was collected, washed, and dispersed in cyclohexane, followed by the addition of C_3_N_4_. After stirring, the composite was isolated, washed, and dried. The dried solid was then mixed with an equal amount of glucose and annealed at 800 °C under an inert atmosphere to obtain HEO@NC. After annealing, it was quickly removed and allowed to cool to room temperature, yielding HEO@NC. For the synthesis of NC, the same conditions were used as for HEO@NC, except that the black colloidal product was omitted during the annealing process.

**Catalytic Effect Test.** Typically, 5 mg of catalyst was added to 50 mL of 4CP solution. NaOH or H_2_SO_4_ was then used to adjust the pH as needed, and inorganic salts were added if required. The appropriate concentration of PI was introduced at the start of the experiment. Samples were collected at various time points, filtered through a filter head, and the 4CP concentration was analyzed using UPLC.

**Analysis methods of iodine species.** IO_4_^-^, IO_3_^-^ and I^-^ were analyzed using UPLC with an ZORBAX Eclipse XDB-C18, 4.6 x 150 mm, 5um column. The eluent composition is 0.1% phosphoric acid and acetonitrile (80:20 v/v) with λ = 228 nm. Quantification was performed by establishing the calibration curves using standard solutions of NaIO_4_, NaIO_3_ and KI, respectively.

**HOI Detection:** HOI reacts rapidly with phenol, forming iodinated phenols such as 2-iodophenol and 4-iodophenol, which can be readily quantified using UPLC. In this study, phenol (1 mM final concentration) was added to the HEO@NC-PI systems prior to initiating the reaction. The HOI produced were analyzed by UPLC using calibration curves from 2-iodophenol and 4-iodophenol standards. The HOI concentration was determined as the combined total of 2-iodophenol and 4-iodophenol.

**Analysis of Chloride Ions:** Chloride ion (Cl⁻) concentrations were determined by ion chromatography (IC-10, Prin-Cen, China). The calibration curve was prepared by serial dilution of a commercial chloride standard stock solution (1 mg/mL) to appropriate concentrations. The chromatographic separation was achieved under isocratic conditions with a 15 mM KOH eluent at a flow rate of 1.0 mL/min. The column compartment and detector cell were maintained at 30 °C and 35 °C, respectively, with a total run time of 10 min. All samples were filtered through 0.22 μm syringe filters prior to injection.

**I**_2_ **Detection:** I_2_ was detected using the starch colorimetric method. To prepare the starch stock solution (10 g/L), 1 g of soluble starch was first mixed thoroughly with 10 mL of distilled water, then added to 100 mL of boiling water. The mixture was boiled for an additional 1 minute under vigorous stirring, and the resulting solution was allowed to cool before use. For I_2_ detection, aliquots of the starch stock solution were added to the samples to achieve a final starch concentration of 1 g/L. The appearance of a blue color indicated the presence of I_2_.

**Fluorescence Spectra:** Fluorescence spectra were recorded using an F-7100 fluorescence spectrophotometer with an emission wavelength range of 350–600 nm. The formation of surface-adsorbed hydroxyl radicals (**^∙^**OH_ads_) was indirectly monitored by measuring the fluorescence emission of 7-hydroxycoumarin at 460 nm (λ_ex_ = 332 nm). Instrument parameters: PMT voltage = 400 V, scan speed = 2400 nm/min. To visualize where **^∙^**OH_ads_ was produced and accumulated, fluorescence microscopy was performed using a Nikon C2 confocal microscope, with 7-hydroxycoumarin serving as the **^∙^**OH_ads_ probe.

***In Situ* Raman spectroscopy.** *In situ* Raman analysis was done with a confocal Raman microscope (CRM) (Alpha300R, WITec GmbH, Germany) equipped with a TEM single-frequency laser (λ=532nm, laser power = 40 mW, WITec GmbH, Germany). The laser light was focused through a 100x oil immersion objective (numerical aperture = 0.9) (Carl Zeiss, Germany) onto the sample and the backscattered Raman signal directed through an optic multifibre (50 um diameter) to a spectrometer (UHTS 300 WITec, Germany) (300 g.mm-1 grating) and detected by the CCD camera (Andor DU401 BV, Belfast, North Ireland). Initially, 1 mg of catalyst was added to a glass groove containing 100 μL of PI. A semiconductor TEM single-frequency laser with a wavelength of 532 nm was used as the excitation light source, providing a laser energy of 6 mW and utilizing a grating with a density of 600 g/mm. It was focused by 20 times long focal lens to vertically illuminate the surface of the catalyst and recorded the spectrum in the range of 400 to 2,200 cm^−1^ was recorded with an integration time of 45s for six times. The data were processed by simple cosmic ray subtraction and weighted average method.

**XAFS Measurements and EXAFS Analysis.** Chemical speciations of Co were determined by K-edge XANES. XANES spectra of Co in HEO@NC powder were obtained in the fluorescence model using the beamline of MEX-1 in the Australian Synchrotron Radiation Facility. Co foil, CoO, and CoPc were selected as references. The corresponding reference samples were mixed with cellulose and measured in transmission mode. Co foil was employed for the calibration. The acquired EXAFS data were processed following standard procedures using the ATHENA (version 0.9.26) module implemented in IFEFFIT software packages for background, pre-edge line, and post-edge line calibrations. Subsequently, the *k^3^*-weighted χ(k) data in the R range, spanning from 0 to 4 Å, were Fourier transformed to real (R) space using the Morlet function parameters kappaMorlet=10 and sigmaMorlet=2, with the assistance of hama_fortran. This transformation was carried out to distinguish the EXAFS contributions from various coordination shells.

**Electrochemical Characterization.** The electrochemical properties of the filter electrodes were evaluated using a CHI 760E electrochemical workstation in a three-electrode configuration. The working electrode was prepared as follows: 10 mg of catalyst was dispersed in a solution containing 5 mL of ethanol and 200 μL of 5% Nafion (as a binder), followed by stirring overnight. Using the drop-casting method, 10 μL of the catalyst ink was applied onto a polished glassy carbon electrode (GCE) and dried at 60°C for 10 minutes. This process was repeated until the catalyst fully covered the GCE surface. All reported current densities were normalized to the geometric surface area.

1. Open circuit potential method (OCPT). Firstly, the prepared HEO@NC-GCE and NC-GCE are soaked in the reaction solution (20 mM Na_2_SO_4_) overnight to maintain a stable potential. Then the open circuit potential of HEO@NC-GCE or NC-GCE was monitored by open circuit potential analysis using a saturated silver chloride electrode as the reference electrode, and all the potential values were normalized to the reversible hydrogen electrode (RHE) before testing. After stabilizing the open circuit potential of the system, PI and 4CP were sequentially added to the solution to monitor the resulting changes in potential. The experiment was conducted for a duration of 3500 seconds with a sampling interval of 0.1 seconds.
2. Cyclic voltammetry (CV). CV was performed between -0.25 V and 1.1 V at a scan rate of 10 mV/s using an electrochemical workstation. The experiments were conducted in the 4CP solution, prepared by 20 mM Na_2_SO_4_ solution, with a three-electrode cell configuration including a working electrode (20×20×1 mm graphite plate electrode), a counter electrode (platinum electrode), and a reference electrode (saturated silver chloride electrode), and all the potential values were normalized to the reversible hydrogen electrode (RHE) before testing.
3. Linear sweep voltammetry (LSV). LSV was conducted using a standard three-electrode setup, consisting of a platinum counter electrode, an Ag/AgCl/KCl reference electrode, and a graphite plate as the working electrode. The electrolyte used was a 20 mM Na₂SO₄ solution containing 4CP. The electrochemical testing parameters included an initial potential of −0.25 V, a final potential of 1.1 V, and a sweep rate of 10 mV/s. The resulting data were analyzed using the Tafel equation to determine the Tafel slopes for evaluating the electron transfer rates.
4. Electrochemical impedance spectroscopy (EIS). To verify the difference in internal resistance of catalysts, EIS was conducted with an alternating current over 10^6^–10^−2^ Hz at a 5 mV increment.
5. Galvanic cell oxidation test. The catalyst was sprayed onto graphite plates to create cathode and anode electrodes. the galvanic experiment took place in an H-type double-chamber electrolytic cell. The cathode chamber was supplied with PI, and the anode chamber was supplemented with 4CP. Both chambers were separated by a cation exchange membrane, with the anode and cathode connected through an ammeter.

**Exposure of zebrafish embryos to NC/HEO@NC and determination of ROS levels.** The AB strain zebrafish were obtained from the National Zebrafish Center (Wuhan, China). Zebrafish were maintained in a recirculating water system at 28.0 ± 1.0 degrees Celsius, under a 14-h light/10-h dark photoperiod, and fed live brine shrimp twice daily. Well-developed embryos were collected at 6 h post-fertilization (hpf) by breeding adult zebrafish in spawning cages. A total of 15 healthy embryos were selected and placed in 6-well plates (8 mL) and exposed to the NC and HEO@NC- treated systems solutions, respectively. These solutions were stored in a refrigerator at 4 degrees Celsius. Before experimentation, they were diluted with deionized water to the desired concentrations. To avoid contamination, dead embryos were removed, and half of the exposure solution was replaced daily for 6 consecutive days.

The embryos exposed to NC and HEO@NC systems solution were first analyzed for ROS levels. As a positive control, zebrafish embryos were pre-treated with hydrogen peroxide (0.1 mM) for 2 h before the assay. Zebrafish embryos without treatment were used as the control group. ROS levels in AB strain zebrafish were detected using 2′,7′-dichlorodihydrofluorescein diacetate (DCFH-DA) (Sigma, USA). DCFH-DA was diluted to a working solution of 10 μmol/L with deionized water. Embryos were incubated in a well plate containing 10 μmol/L DCFH-DA for 30 min in the dark and then rinsed three times with deionized water for ROS detection. Images were captured using a confocal microscope.

**Measurement of oxidation products.**

In the analysis of reaction products for 4CP, the initial concentrations of 4CP and PI were set at 0.1 mM and 0.5 mM, respectively, with a catalyst loading of 0.1 g/L. To investigate products accumulated in the solid phase, solid samples were separated by filtration and eluted with organic solvents (tetrahydrofuran and toluene) to extract transformation products. Liquid samples were concentrated using a C18 extraction column and eluted with methanol to isolate the conversion products. Oxidation products of 4CP were identified using ultra-high performance liquid chromatography coupled with quadrupole time-of-flight mass spectrometry (UPLC-QTOF-MS). The UPLC conditions were as follows: a ZORBAX Eclipse Plus C18 column (2.1 × 100 mm, 1.8 μm) was used with water (mobile phase A) and methanol (mobile phase B), both containing 5 mM ammonium acetate. The flow rate was set at 0.3 mL/min with detection at 280 nm. The gradient elution program was as follows: A:B= 95%:5% at 0 min, maintained at 95%:5% until 1 min, then shifted to 5%:95% from 1 to 6 min, held at 5%:95% from 6 to 8 min, and returned to 95%:5% from 8 to 10 min. The injection volume was set to 1.5 μL, with a chamber volume of 200 μL, and the column temperature held constant at 40 ℃. Mass spectrometry operating conditions: desolventizing gas was nitrogen at a flow rate of 600 L/h, desolventizing gas temperature was 450 °C; ion source was ESI in negative ion mode; temperature was 100 °C and the collision voltage was set to 2.5 kV.

**Long-term continuous flow Treatment using HEO@NC.** A long-term degradation of 4CP test was conducted to evaluate the practical application performance of the HEO@NC. The custom-designed fixed-bed column (approximately 10 mm in diameter) was packed with 300 mg of HEO@NC powder along with polypropylene fiber cotton, achieving a bed depth of 9 mm. To prevent the escape of HEO@NC powder during continuous-flow operation, custom-made polyethylene sieve plates (10.1 mm diameter, 1.6 mm height, 20 µm pore size) were placed at both the upper and lower sections of the fixed-bed column to securely contain and compact the HEO@NC. Additionally, polyester fibers were used for secondary containment and to ensure stable water flow throughout the system. The experimental solution was prepared by spiking tap water with 4CP to simulate wastewater conditions. A feed solution containing 0.1 mM 4CP and 1 mM PI was introduced into the flow-through reactor at a flow rate of 1.48 mL/min. Upon collecting 50 mL of effluent, the 4CP concentration was analyzed using UPLC to evaluate the treatment efficiency of the system.

**Cell Culture and Cell Viability Assay.** The 16HBE cell lines were cultivated in RPMI-1640 medium, a formulation developed by the Roswell Park Memorial Institute, supplemented with 10% fetal bovine serum (FBS) and 1% penicillin/streptomycin (P/S). This carefully balanced composition aims to foster robust cell growth while ensuring the maintenance of a sterile culture environment. Cells were maintained at 37 °C in a humidified incubator with a 95% air and 5% CO_2_ atmosphere. A total of 2×10^4^ cells per well were seeded into a 96-well plate and cultured until reaching the desired growth phase, after which they were exposed to various concentrations of the test samples. For HEO@NC, the tested concentrations were 0, 62.5, 125, 250, and 500 ppm. For Co^2+^, the concentrations were 0, 2.5, 5, 10, and 20 ppm. After a 48-hour incubation, the culture plate was gently removed. Then, 100 μL of the CellTiter-Lumi^TM^ Luminescent Cell Viability Assay Kit (CTL) was added to each well. The plate was shaken gently for 2 minutes to ensure thorough mixing, followed by a 10-minute incubation. Next, 70 μL of the reaction mixture from each well was transferred to a new 96-well plate. The luminescence intensity was measured using a microplate reader with a luminometer, providing an accurate assessment of cell viability. All experiments were conducted in triplicate.

**Theoretical Calculation Studies.** Density Functional Theory (DFT) analyses were conducted using the Vienna ab initio Simulation Package (VASP), applying the projector augmented-wave (PAW) method for precision. The Perdew, Burke, and Ernzerhof (PBE) model within the generalized gradient approximation (GGA) was selected to effectively depict the exchange-correlation dynamics. A 2 × 2 × 1 K-point grid in the Brillouin zone with a raised energy barrier of 650 eV was used to ensure thorough surface examination. Optimization processes continued rigorously until the energy and mechanical forces reached minuscule levels of 1 × 10^–5^ eV and 0.02 eV/Å, respectively. A substantial vacuum boundary of 15 Å was incorporated to segregate the periodic surface arrays, minimizing external influences. The advanced DFT-D3 methodology with Grimme's zero damping configuration was employed for the Van der Waals (vdW) interactions. The pollutant underwent molecular structure optimization at the DFT/B3LYP/6-311G+(d,p) level using Gaussian 16. Subsequent wavefunction analysis was conducted via Multiwfn software^1,2^. Hirshfeld charges were employed to derive electron densities for cationic, neutral, and anionic states, enabling calculation of Fukui functions. Visualization of ESP, average local ionization energy (ALIE), and Fukui function, computed by Multiwfn, was implemented in VMD 1.9.

**Quantum chemical computational method.** All quantum chemical computations were carried out with the GUASSIAN-16 program package. All structures of reactants, transition states, and products in the reactions involved in this study were fully optimized using M062X-D3 functional in conjunction with the def2-TZVP basis set, along with the implicit SMD solvent model. The vibrational frequencies were calculated at the same level of theory to characterize the nature of the stationary points, and all wave functions were checked to be stable. The minimum energy path (MEP) was obtained using intrinsic reaction coordinate (IRC) calculations to confirm the connection of the transition state with the corresponding reactant and product.


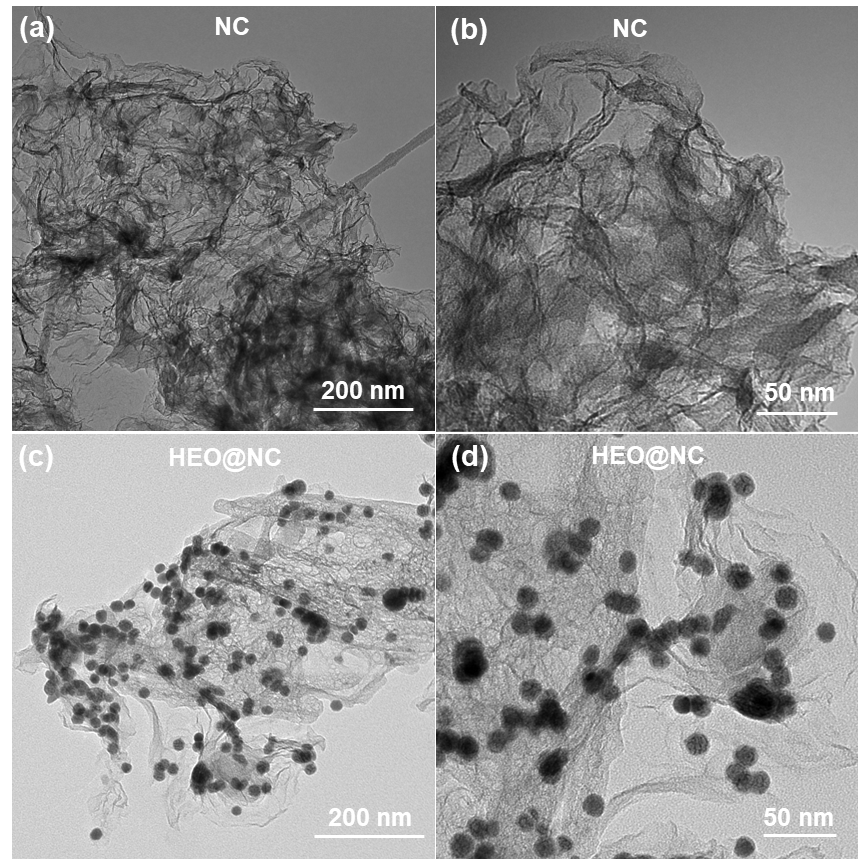


**Fig. S1 |** TEM images of NC and HEO@NC.


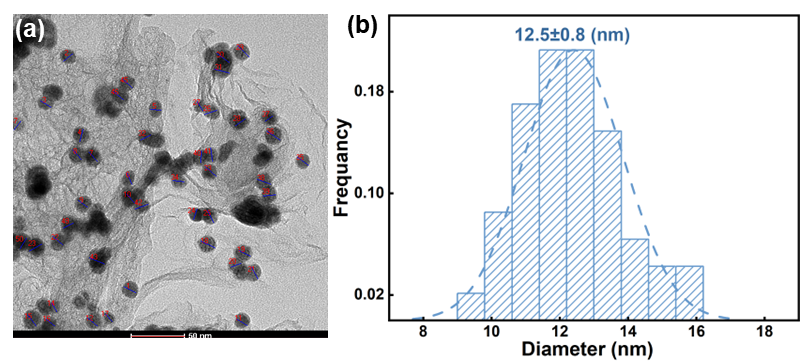


**Fig. S2 |** The size distribution of HEOs.


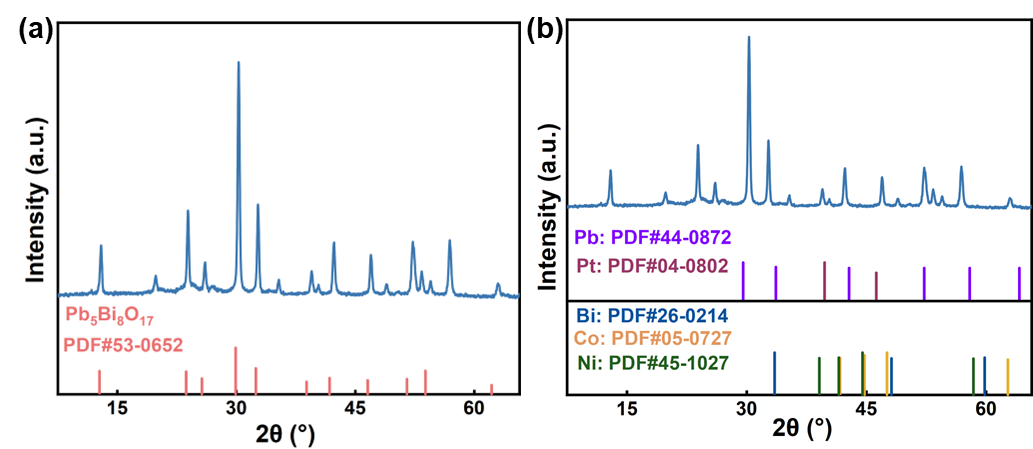


**Fig. S3 |** XRD pattern HEOs and corresponding standard PDF cards.


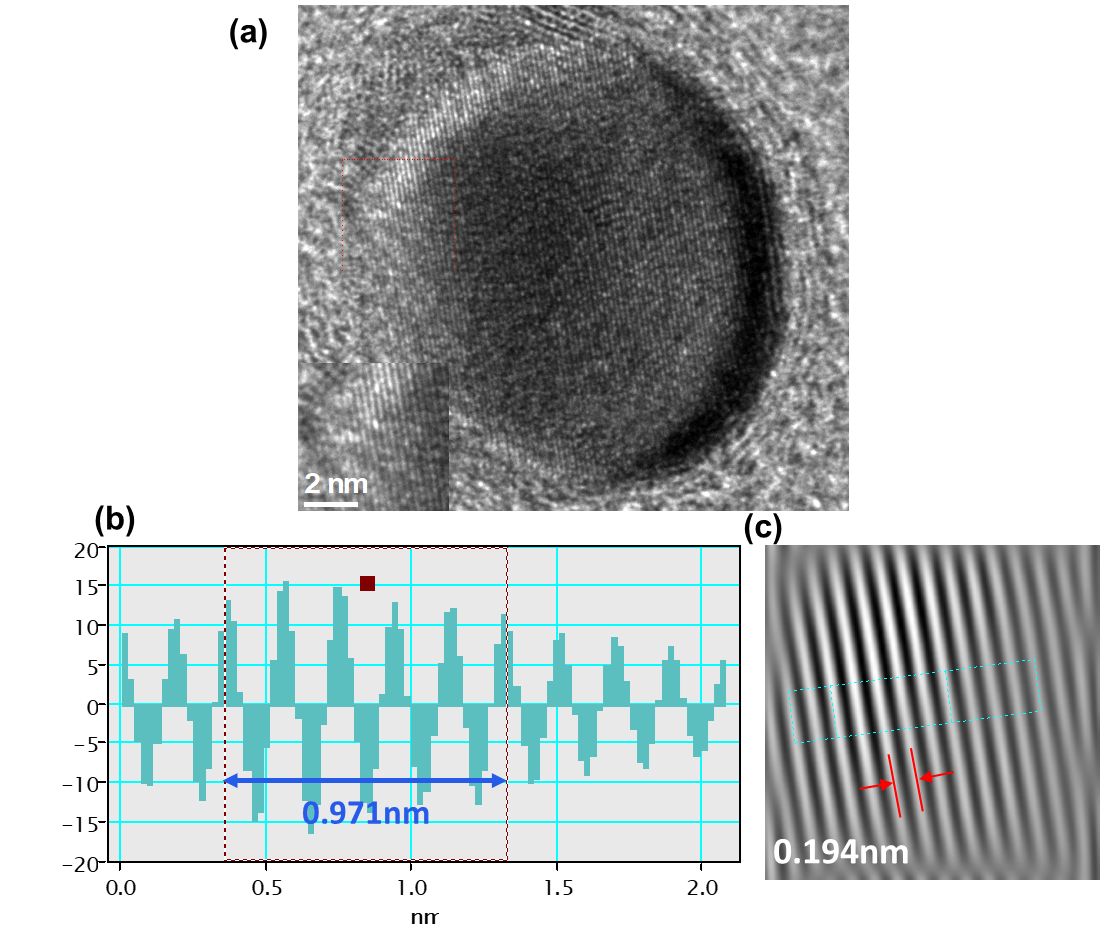


**Fig. S4 |** (a) HRTEM image (scale bar, 5 nm), (b) the corresponding inverse FFT lattice fringe pattern of HEOs, and (c) the interplanar spacing diagram corresponding to the (2 0 0) plane.


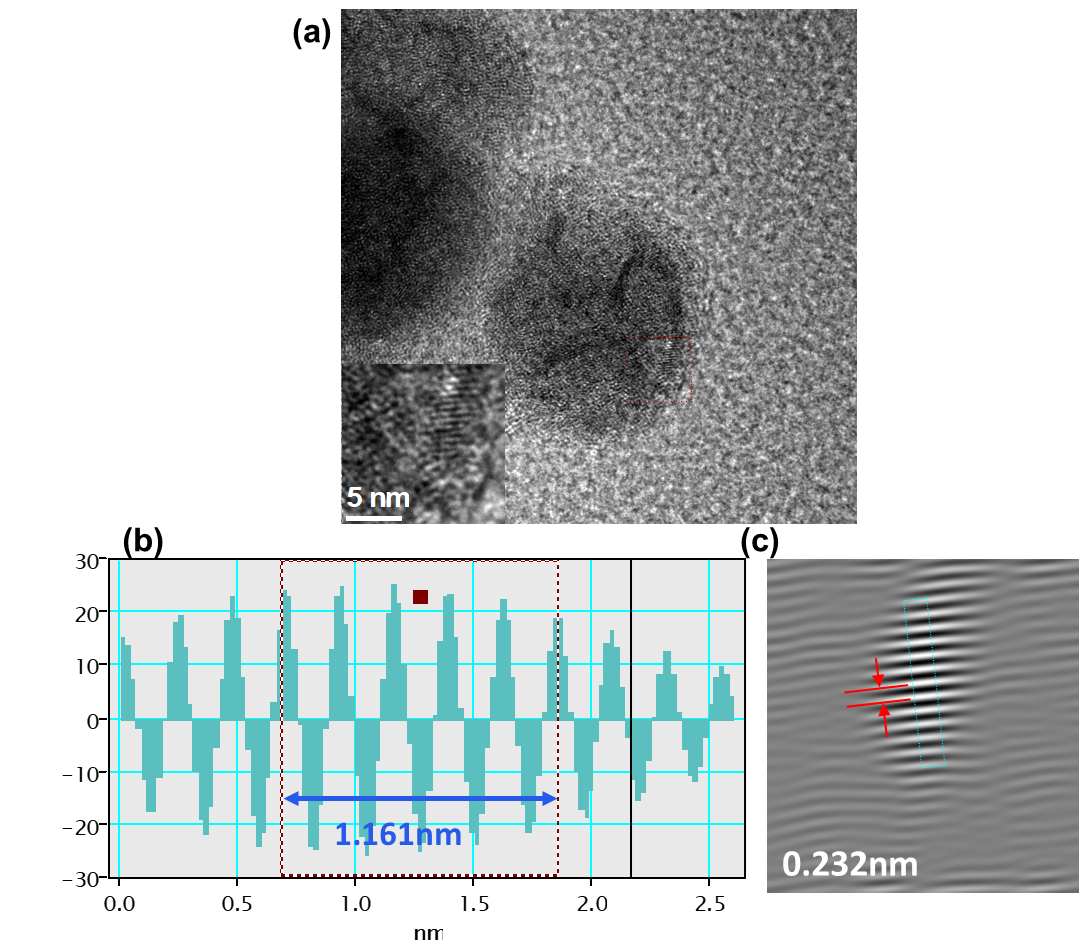


**Fig. S5 |** (a) HRTEM image (scale bar, 5 nm), (b) the corresponding inverse FFT lattice fringe pattern of HEOs, and (c) the interplanar spacing diagram corresponding to the (0 0 6) plane.


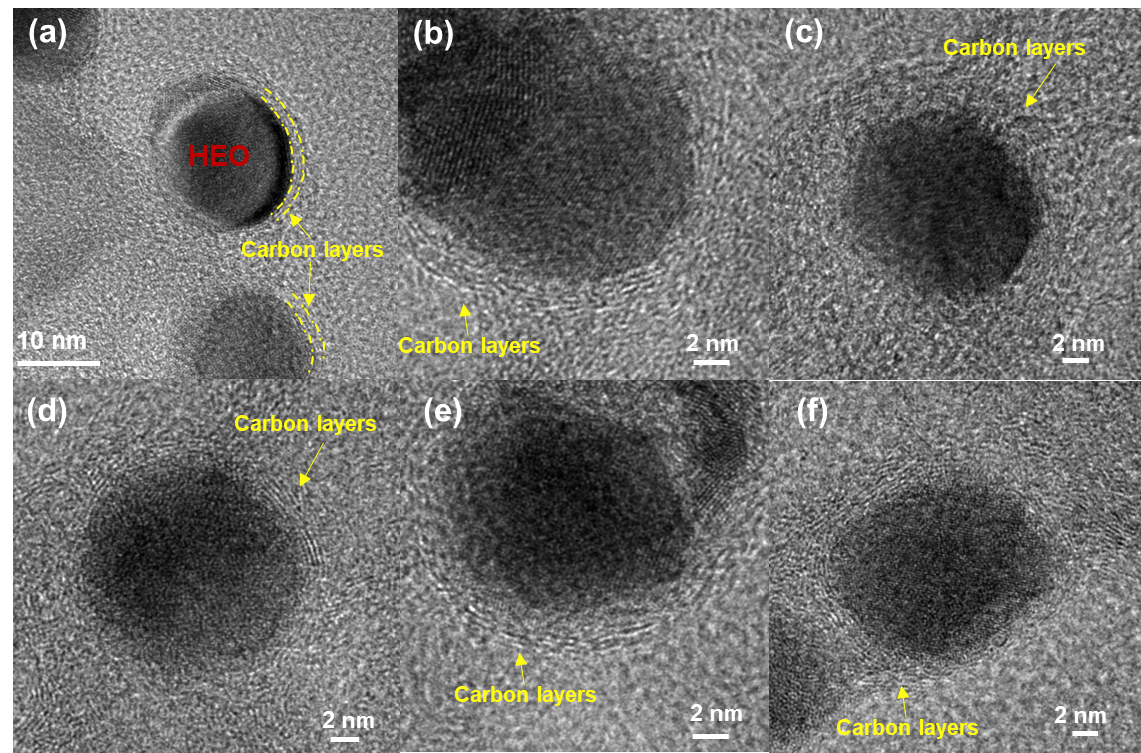


**Fig. S6 |** HRTEM images of HEO@NC present the HEOs encapsulated by the carbon layers.

Through systematic bright-field transmission electron microscopy (BF-TEM) characterization, pronounced self-encapsulation structures were clearly observed in multiple HEOs, accompanied by interfacial structural defects in carbon shells. Such synergistic self-encapsulation–defect features strengthen interfacial interactions and thereby markedly enhance the structural stability of HEOs under harsh reaction conditions.


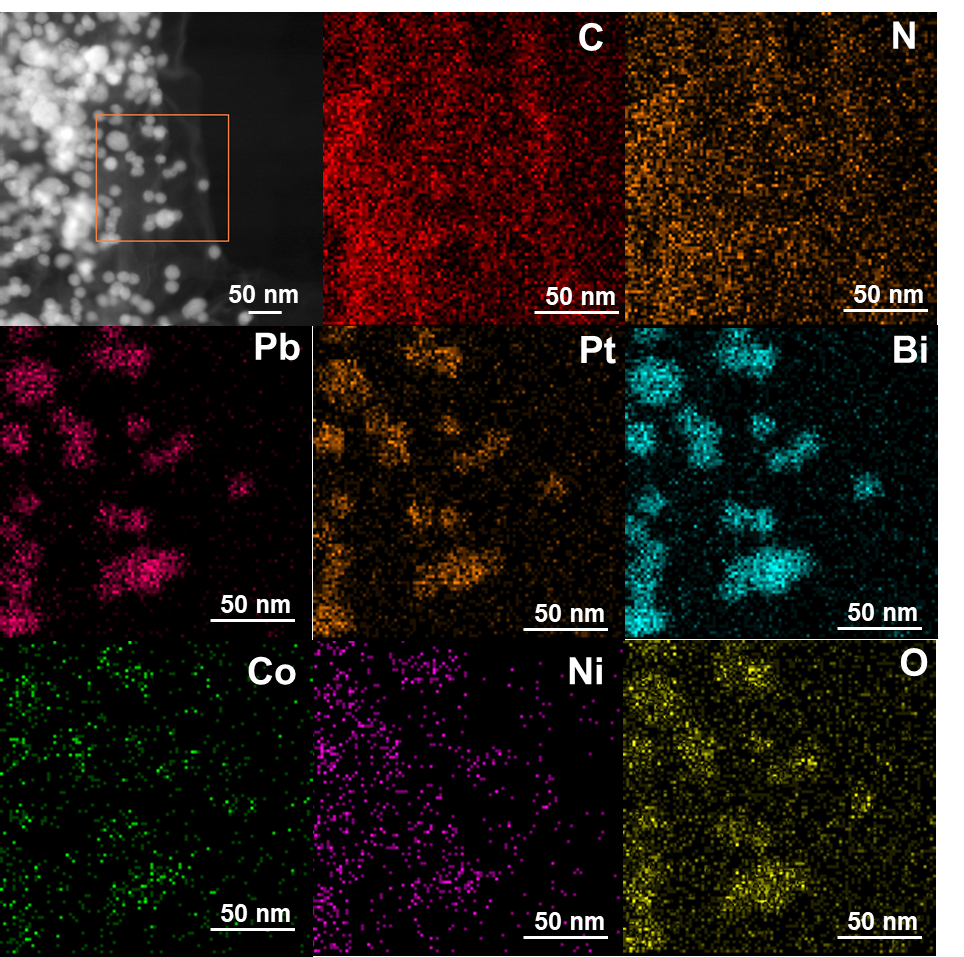


**Fig. S7 |** EDS mapping images show the uniform dispersion of C, N, Pb, Pb, Bi, Ni, Co, and O elements in a HEO@NC.


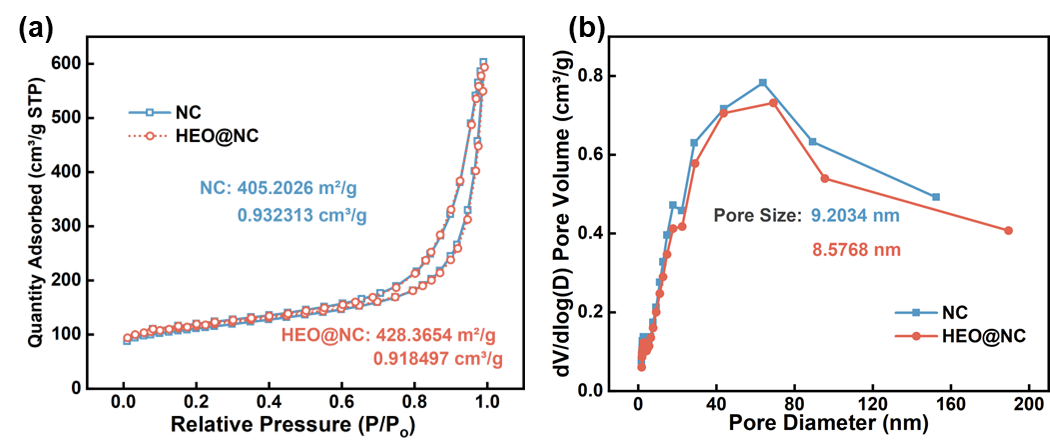


**Fig. S8 |** (a) N_2_ sorption isotherms curve and (b) pore size distribution of HEO@NC and NC.


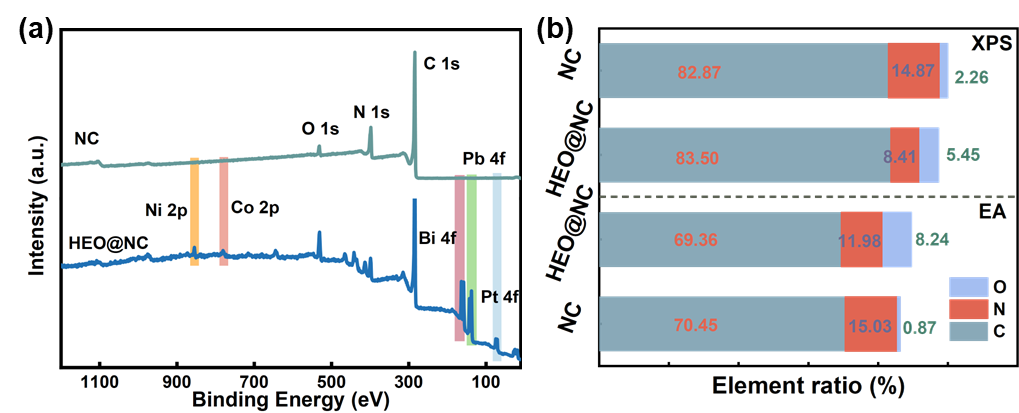


**Fig. S9 |** (a) The wide-scanning XPS spectrum of HEO@NC. (b) Element proportion of C N O in NC and HEO@NC obtained by elemental analysis and XPS analysis.


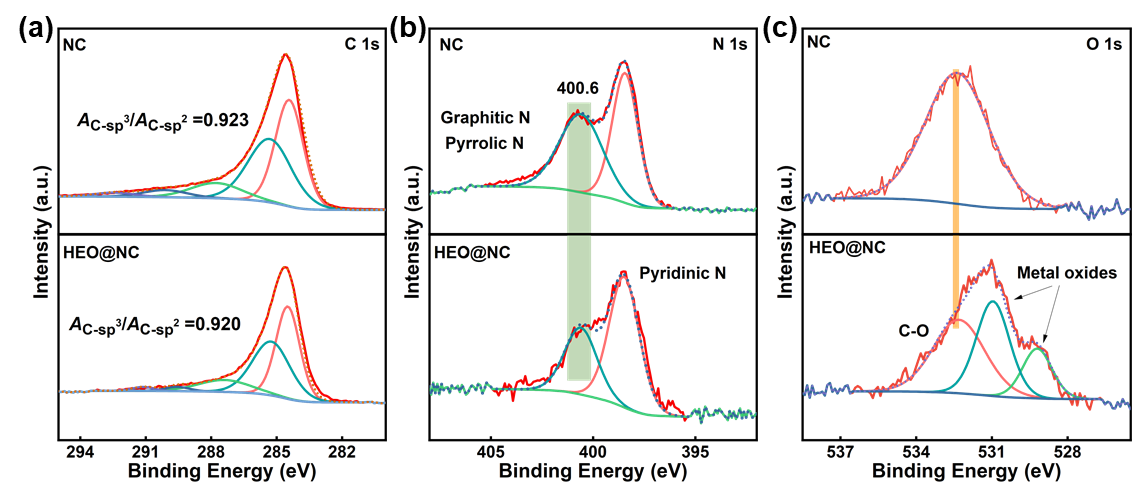


**Fig. S10 |** High-resolution spectra of HEO@NC: (a) C 1s, (b) N 1s, and (c) O 1s.

The high-resolution C 1s spectra show that the ratio of C-sp^3^ to C-sp^2^ peak intensities changes very little after the loading of HEOs particles on the carbon-nitrogen support, indicating that there is not much difference in the content of disordered lattice carbon and graphitic degree. Similarly, the N 1s spectrum exhibits consistent characteristics, indicating that the thermal treatment did not alter the nitrogen species after loading HEOs particles. The O 1s spectrum shows a distinct metal oxide peak, further confirming the successful loading of HEO particles.


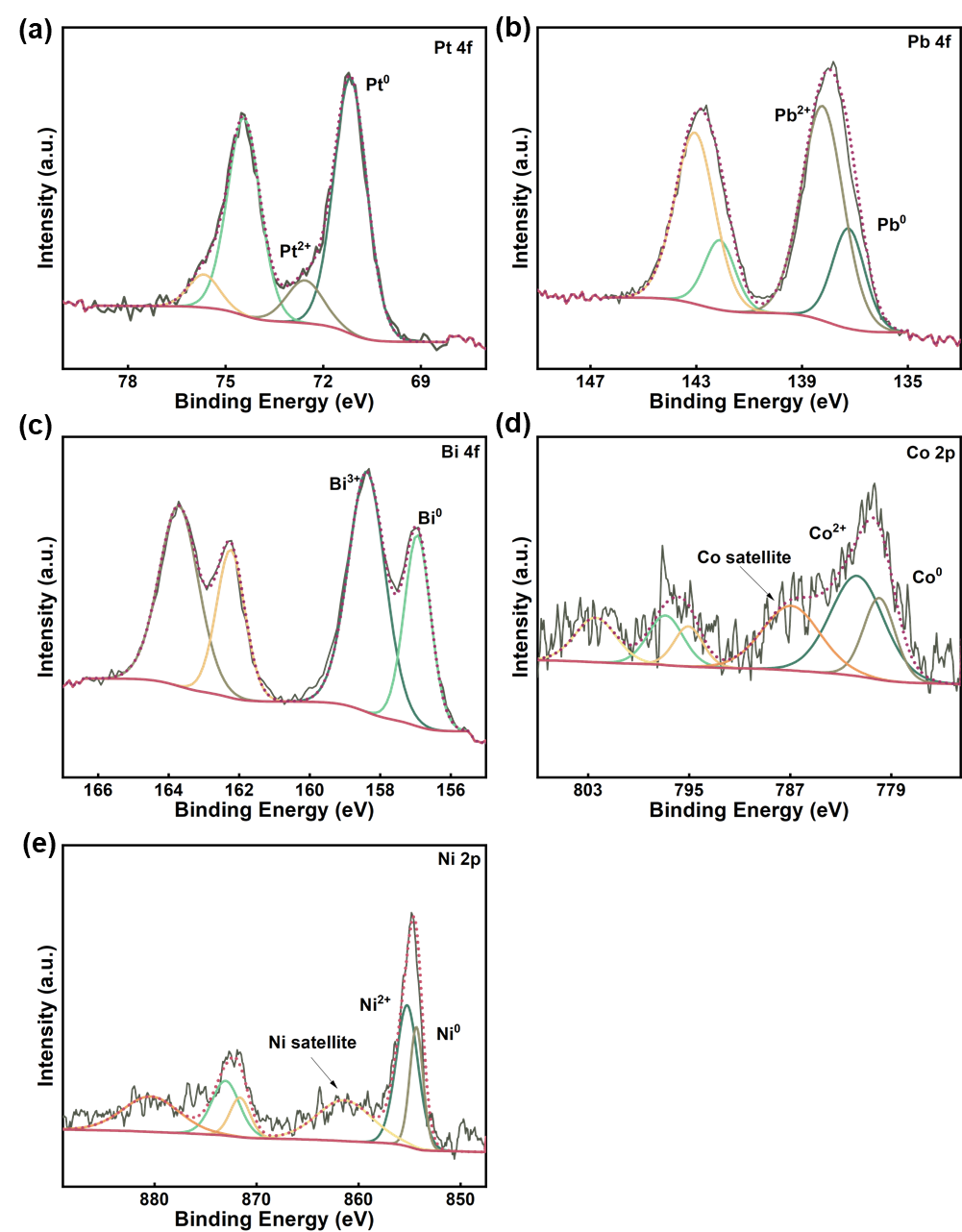


**Fig. S11 |** The high-resolution spectra of HEO@NC, (a) Pt 4f, (b) Pb 4f, (c) Bi 4f, (d) Co 2p, and (e) Ni 2p.

The high-resolution XPS of Pt 4f displays two peaks at 71.16 and 74.44 eV, corresponding to Pt^0^ and Pt^2+^, respectively. The Pb 4f XPS reveals two peaks at 137.2 and 138.2 eV, indicating the presence of Pb^0^ and Pb^2+^. Similarly, the Bi 4f XPS shows peaks at 156.9 and 158.4 eV, associated with Bi^0^ and Bi ^3+^, respectively. The Co 2p XPS is deconvoluted into three peaks: the first two at 779.9 and 781.7 eV correspond to Co^0^ and Co^2+^, while the third peak at 786.9 eV is attributed to the satellite of Co^2+^. The Ni 2p XPS is also deconvoluted into three peaks, with those at 854.3 and 855.2 eV corresponding to Ni^0^ and Ni^2+^, and the third peak at 861.4 eV representing the satellite of Ni^2+^.


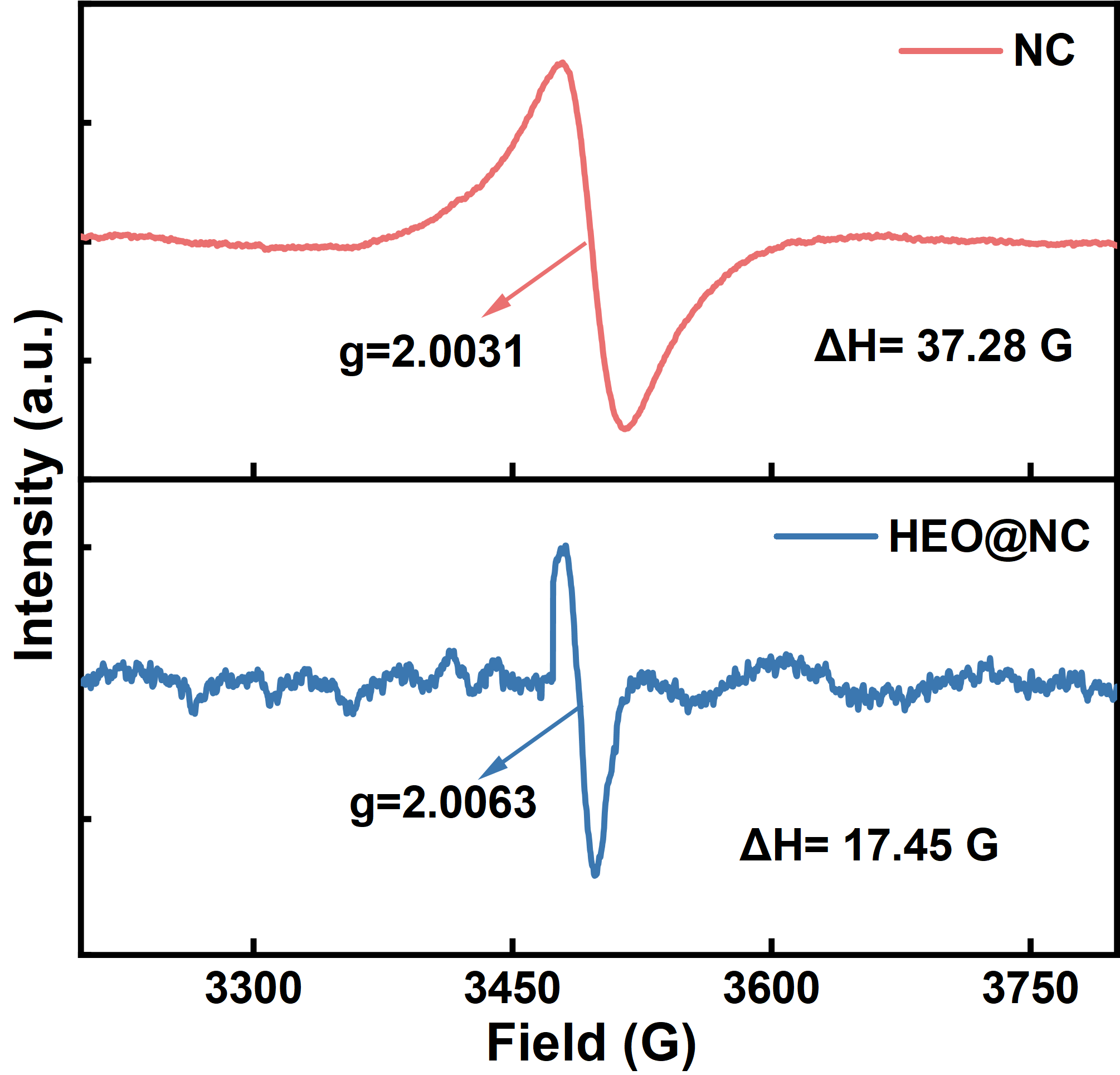


**Fig. S12 |** ESR spectra of the NC and HEO@NC.


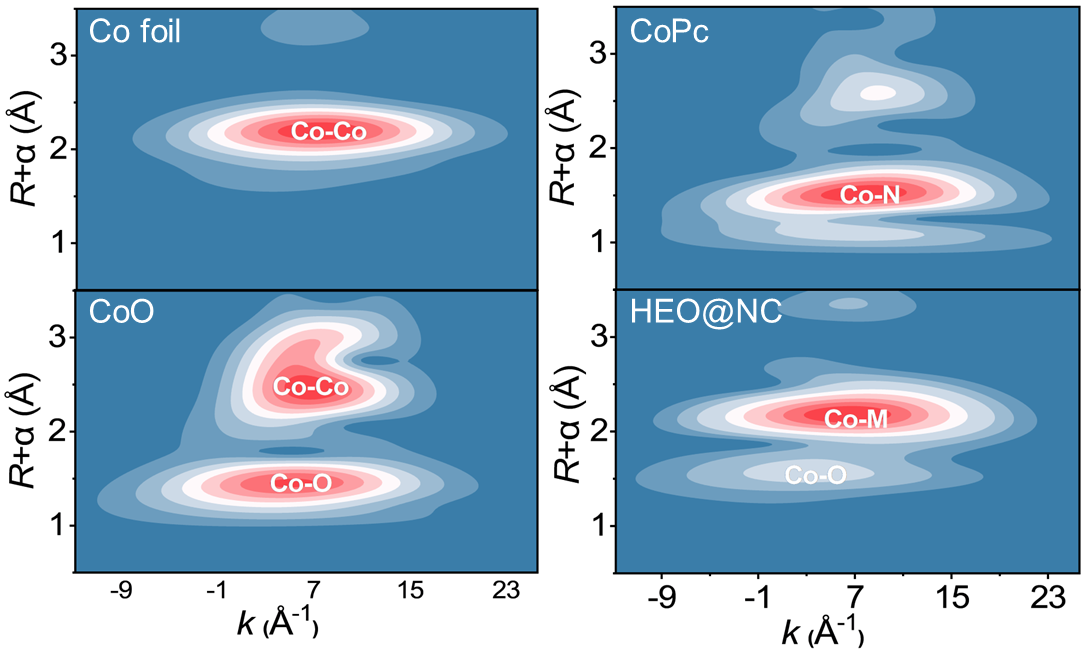


**Fig. S13 |** WT of EXAFS spectra of HEO@NC, Co foil, CoPc, and CoO.


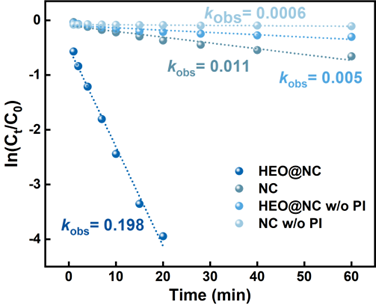


**Fig. S14 |** Comparison between the apparent rate constants of HEO@NC，NC, and the absence of PI.


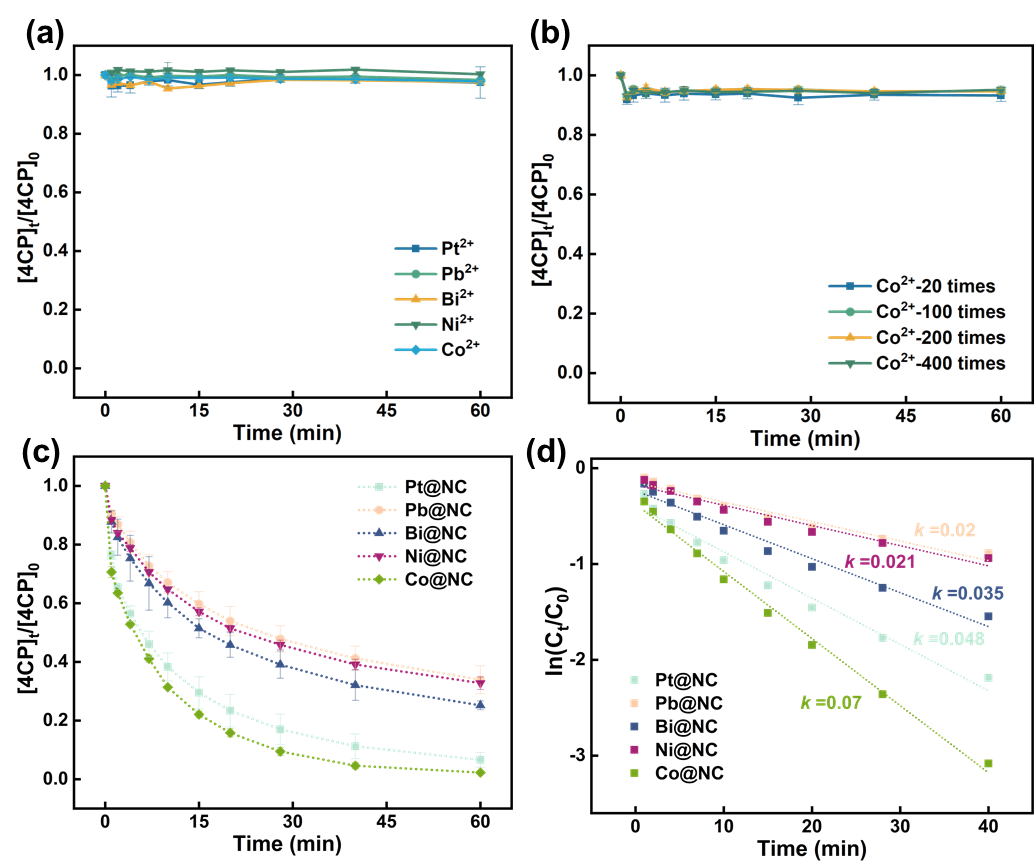


**Fig. S15 |** Degradation activity of (a) ions quantified by their loading on HEO@NC surfaces and (b) different equivalents of Co^2+^ is individually employed as catalysts. (c) Degradation rates and (d) apparent kinetic constants for 4CP using catalysts synthesized from various single metals compositions. Dosage: [4CP]_0_: 0.1 mM, PI: 0.5 mM, reaction solution: 50 mL, catalyst: 0.1 g/L, reaction time: 60 min.

To elucidate the superior performance of HEO@NC in 4-CP removal, a series of control experiments were conducted using individual metal ions (Pb^2+^, Bi^2+^, Pt^2+^, Ni^2+^, and Co^2+^) as comparative catalysts at equimolar concentrations corresponding to the metal content in HEO@NC. At a PI concentration of 0.5 mM, none of the metal ions exhibited significant removal performance. To further assess the role of cobalt, additional degradation experiments were carried out at higher concentrations, including both free Co^2+^ ions and cobalt species loaded onto the NC surface. Remarkably, even with a 400-fold increase in Co^2+^ concentration, no observable degradation of 4-CP occurred These results highlight the unique synergistic effect and structural advantages of HEO@NC that cannot be replicated by individual metal ions or simple metal loading.


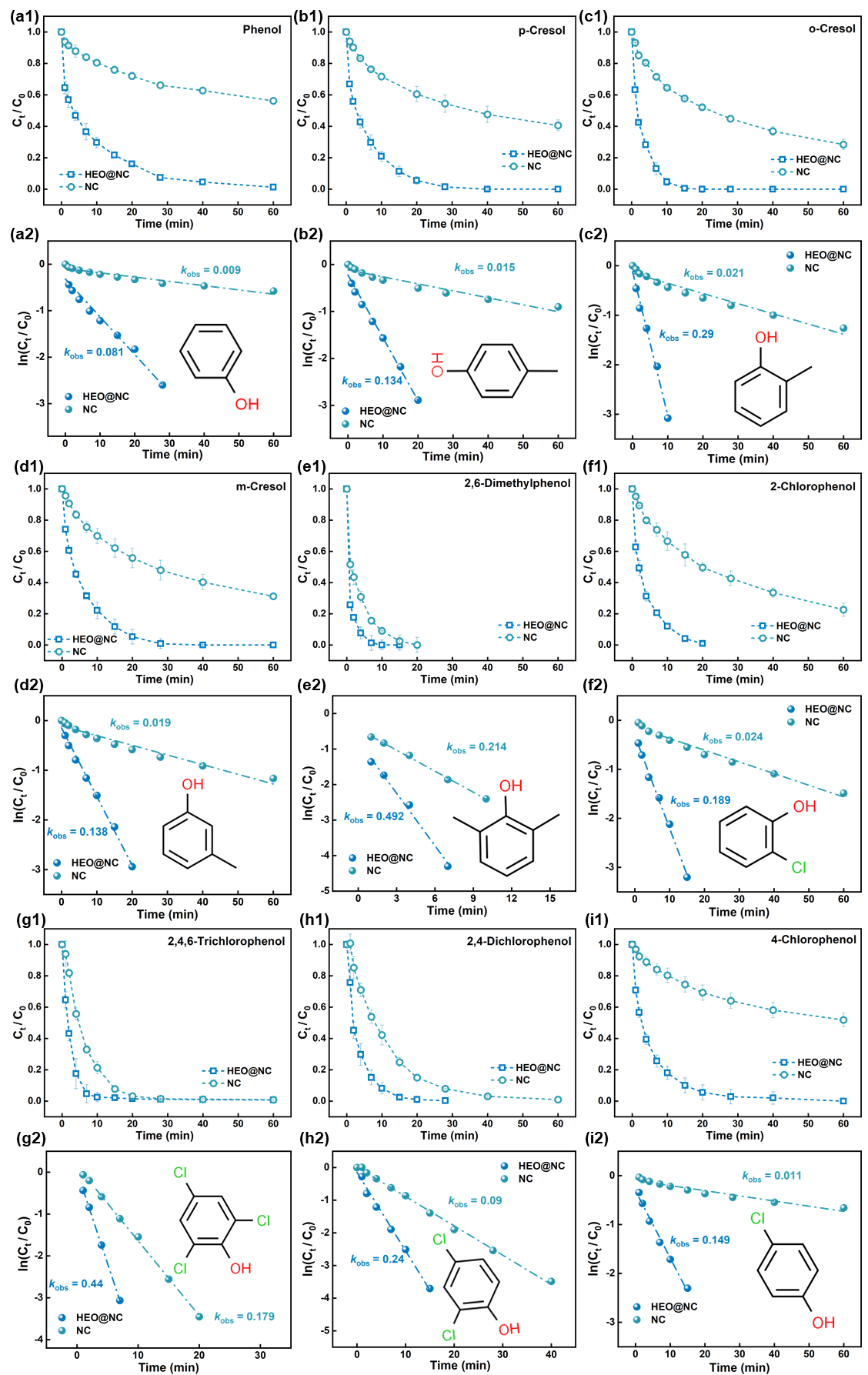


**Fig. S16 |** Removal rates of different pollutants in the HEO@NC–PI system and the NC–PI system. (a) Phenol; (b) *p*-Cresol; (c) *o*-Cresol; (d) *m*-Cresol; (e) 2,6-Dimethylphenol; (f) 2-Chlorophenol; (g) 2,4,6-Trichlorophenol; (h) 2,4-Dichlorophenol; (i) 4-Chlorophenol. Dosage: [Pollutants]_0_: 0.1 mM, PI: 0.5 mM, reaction solution: 50 mL, catalyst: 0.1 g/L.


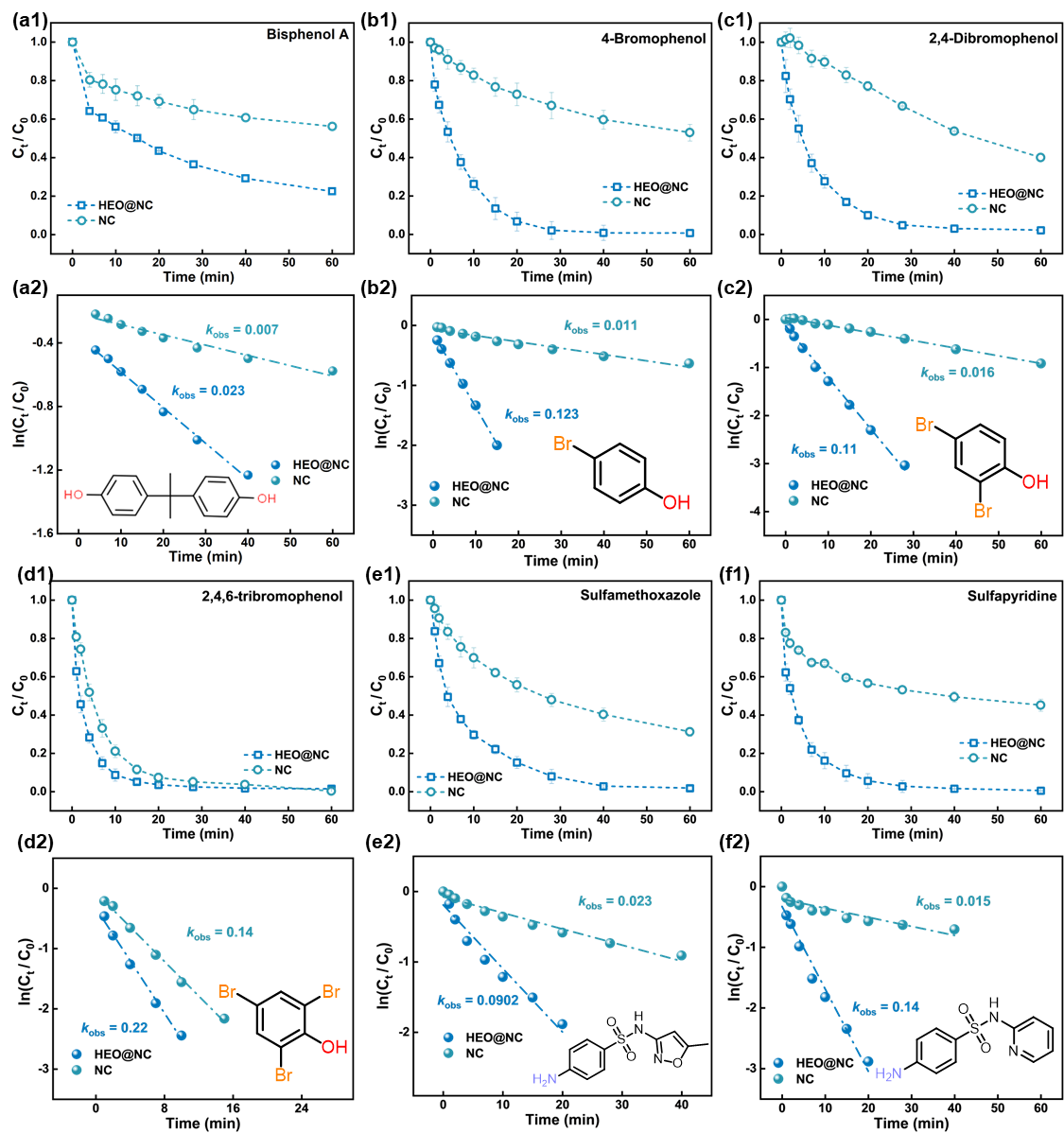


**Fig. S17 |** Removal performances of different pollutants in the HEO@NC–PI system and the NC–PI system. (a) Bisphenol A; (b) 4-Bromophenol; (c) 2,4-Dibromophenol; (d) 2,4,6-Tribromophenol; (e) Sulfamethoxazole; (f) Sulfapyridine. Dosage: [Pollutants]_0_: 0.1 mM, PI: 0.5 mM, reaction solution: 50 mL, catalyst: 0.1 g/L.

For phenolic compounds and their halogenated derivatives (e.g., chlorophenols and bromophenols) featuring a single benzene ring as the basic structural unit and pronounced π-conjugation, the system exhibits excellent removal efficiency and rapid oxidative polymerization kinetics, demonstrating broad applicability toward aromatic pollutants with relatively simple structures and favorable electronic properties. In contrast, for pollutants with polycyclic structures or more complex molecular frameworks, although effective removal can still be achieved, the polymerization rate is significantly reduced. This behavior mainly arises from enhanced steric hindrance associated with larger molecular sizes and restricted mass transfer toward interfacial active sites. Consequently, in such systems, the overall reaction process is more predominantly governed by diffusion effects and molecular structural accessibility rather than interfacial electronic coupling.


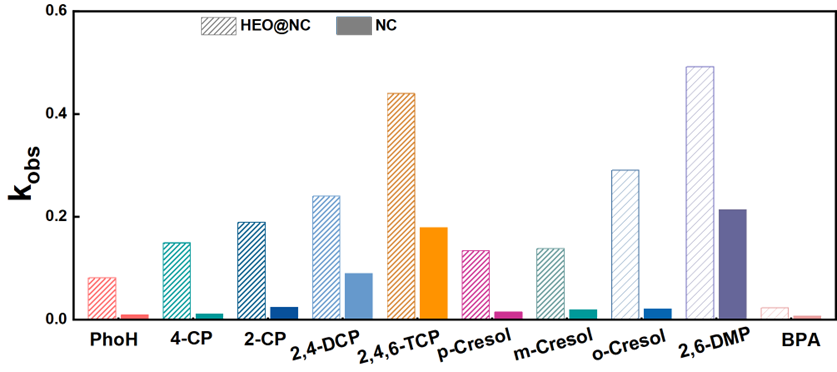


**Fig. S18 |** Apparent kinetic constants of HEO@NC and NC removal different pollutants.


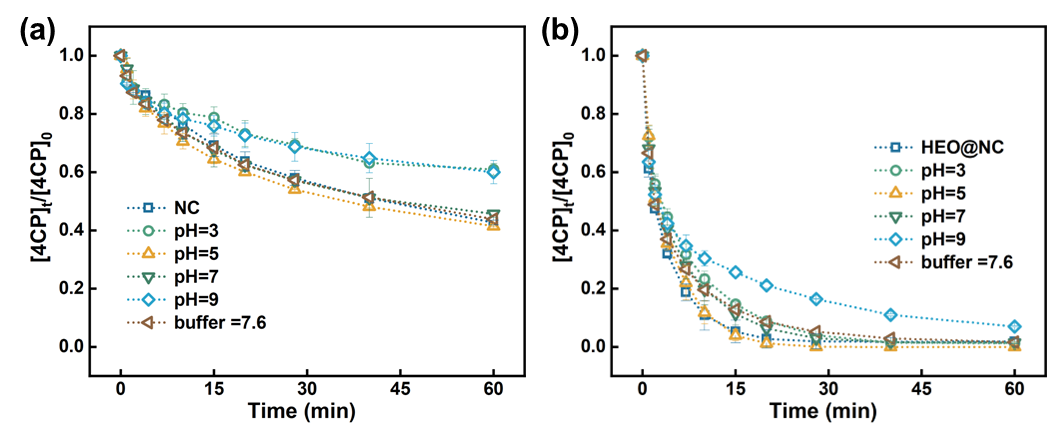


**Fig. S19 |** Degradation activity of HEO@NC and NC under different pH and borate buffer environments. Dosage: [4CP]_0_: 0.1 mM, PI: 0.5 mM, reaction solution: 50 mL, catalyst: 0.1 g/L, reaction time: 60 min.

In systems involving periodate activation, Bokare et al^3^. observed that raising the pH above 8.5 unexpectedly reduced the oxidation efficiency of organic contaminants. This reduction was attributed to the transformation of periodate from IO_4_^−^ to the less reactive H_2_I_2_O_10_^4−^. Similarly, Chadi and Kim^4,5^ reported that the removal efficiency of organic contaminants decreased when using H_2_O_2_ to activate periodate as the initial pH increased from 2 to 11. This suggests that in the HEO-PI system, IO_4_^−^ is the dominant periodate species at pH levels below 8, but it converts to the less reactive H_2_I_2_O_10_^4−^ at higher pH levels.


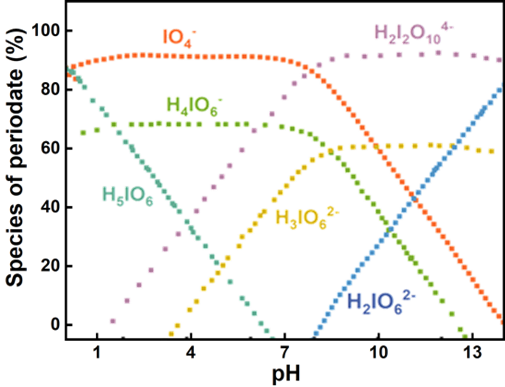


**Fig. S20 |** pH-dependent species of periodate, which was re-plotted according to Environmental Science & Technology, 2022, 56(14): 10372–10380 and J. Photochem. Photobiol. A 2004, 165, 35-41.

The meta-ortho transformation can occur during the dissolution of solid metaperiodate (e.g., NaIO_4_), leading to the formation of orthoperiodic acid (H_5_IO_6_), which may further deprotonate into H_4_IO_6_^−^, H_3_IO_6_^2−^, and H_2_IO_6_^3−^, depending on the pH. Chen et al^6^. found that increasing the initial pH from 2 to 11 significantly accelerates the decay rates of organic contaminants but decrease the amounts of organic contaminant removal. While H_4_IO_6_^−^ and H_2_I_2_O_10_^4−^ can serve as precursors for hydroxyl radicals (HO·), the HEO@NC system does not exhibit accelerated degradation rates, implying the absence of ^•^OH activity in this system.


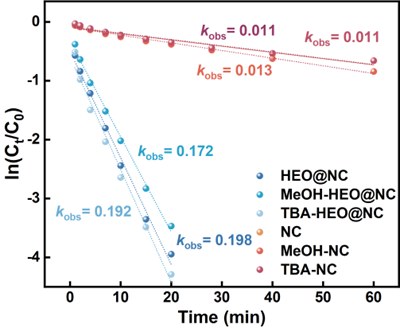


**Fig. S21 |** The apparent rate constants for Quenching effects by various scavengers. Dosage: [4CP]_0_: 0.1 mM, scavengers/PI=100, reaction solution: 50 mL, catalyst: 0.1 g/L, reaction time: 60 min.


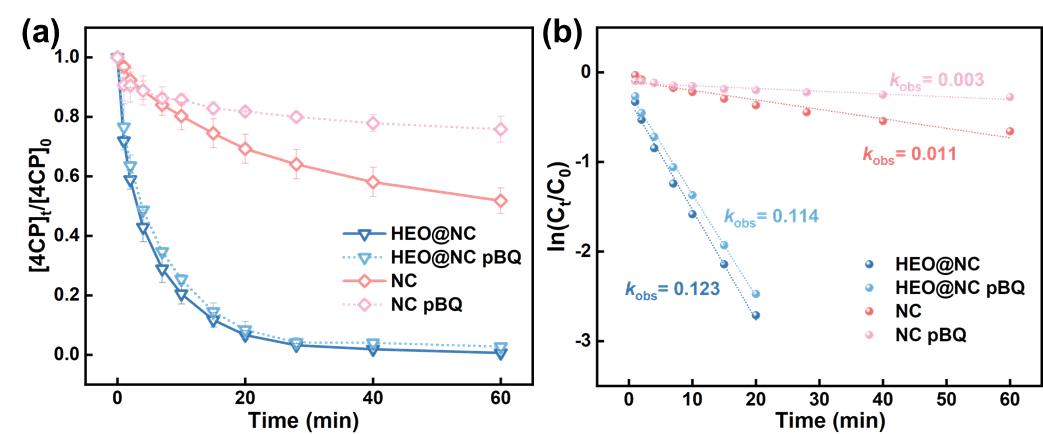


**Fig. S22 |** Quenching effects by pBQ. Dosage: [4CP]_0_: 0.1 mM, PI: 0.5 mM, [pBQ]_0_: 2.5 mM, reaction solution: 50 mL, catalyst: 0.1 g/L.


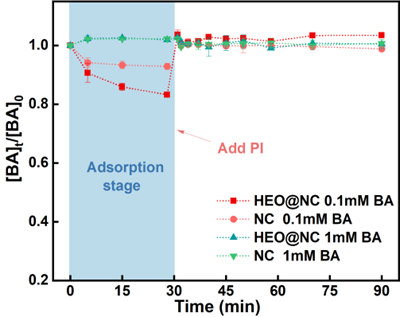


**Fig. S23 |** Degradation activity of HEO@NC and NC with BA as probe. Dosage: [BA]_0_: 0.1 mM/1mM, PI: 0.5 mM, reaction solution: 50 mL, catalyst: 0.1 g/L, reaction time: 60 min.


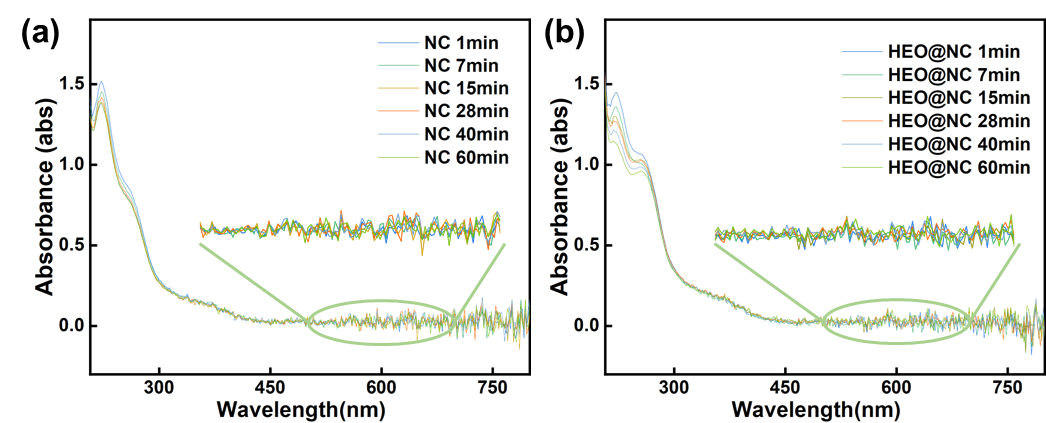


**Fig. S24 |** The absorption spectra of NBT as a probe in NC-PI and HEO@NC-PI systems. Dosage: [NBT]_0_: 0.1 mM, PI: 0.5 mM, reaction solution: 50 mL, catalyst: 0.1 g/L, reaction time: 60 min.

There was no discernible generation of monoformazan and diformazan, as evidenced by the absence of absorption peaks between 500 and 700 nm in the UV−vis spectrum.


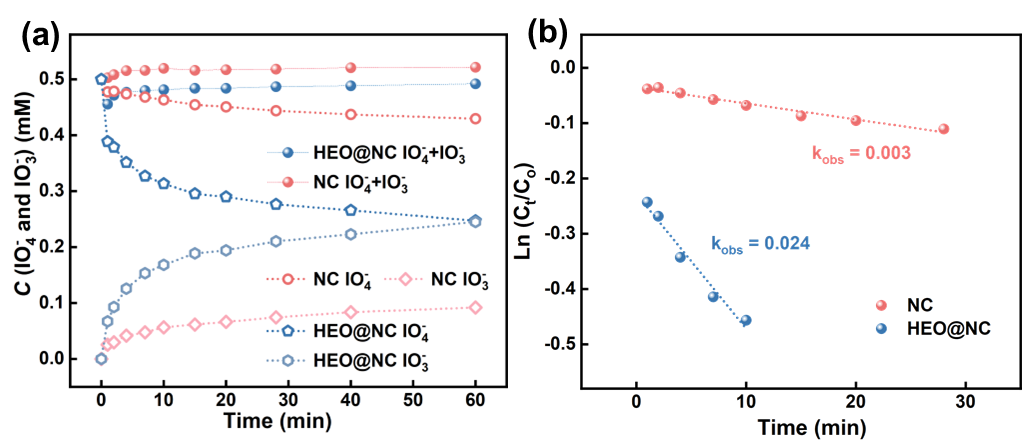


**Fig. S25 |** Decomposition rate (a) and kinetic constants (b) of PI with the catalytic effect of HEO@NC and NC.

Furthermore, monitoring the PI consumption rate indicates that the superior performance of the HEO@NC catalyst is likely attributed to its significantly enhanced ability to activate PI molecules on the surface of HEOs, resulting in a decomposition rate that is approximately 8 times higher than that observed for the NC system. This accelerated activation not only improves the overall reaction kinetics but also contributes to more efficient utilization of the oxidant. In parallel, analysis of iodine-containing species revealed that the total concentrations of IO_3_^−^ and IO_4_^−^ remained constant throughout the entire reaction process, indicating that no additional or unexpected iodine species were generated. This observation rules out side reactions involving iodine transformation and further supports the specificity and stability of the oxidative pathway.


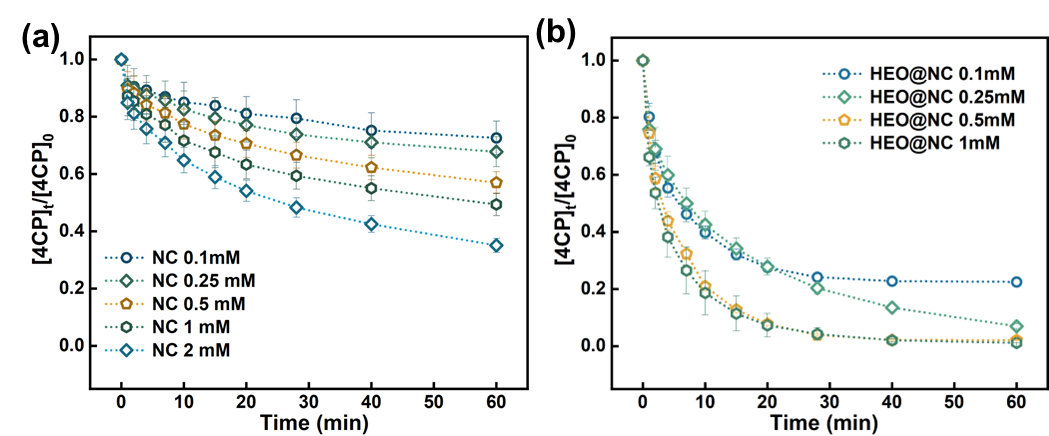


**Fig. S26 |** Degradation activities of HEO@NC (a) and NC (b) at different PI concentration.


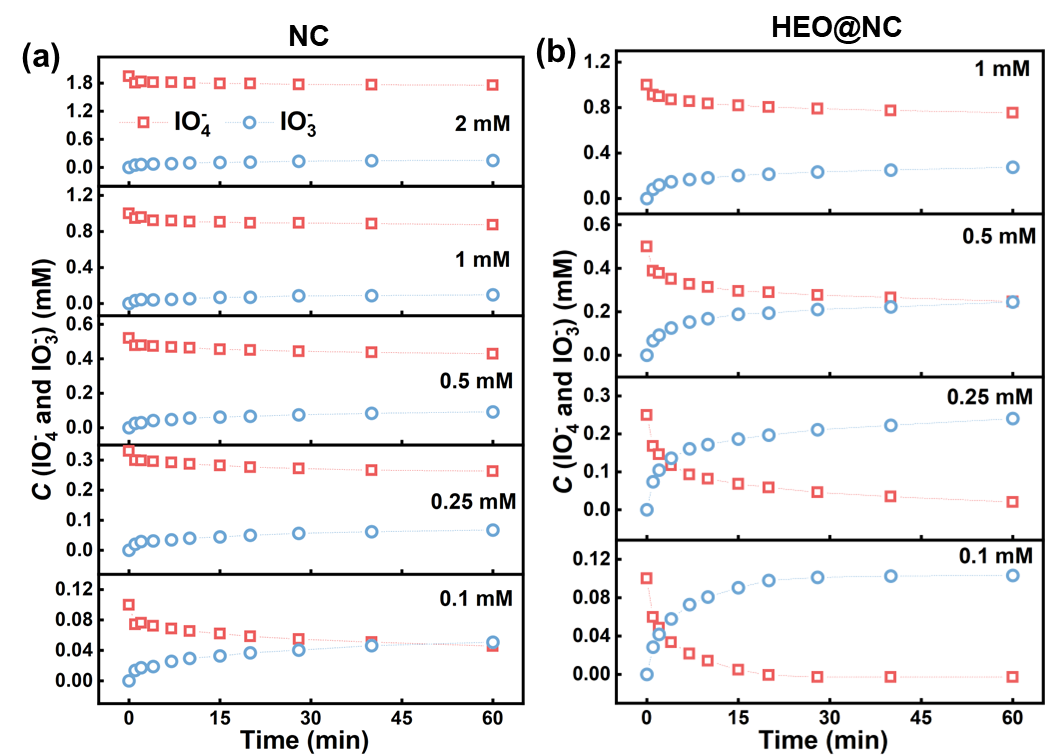


**Fig. S27 |** Decomposition rate of PI and generation of IO_3_^-^ with the catalytic effect of NC (a) and HEO@NC (b).


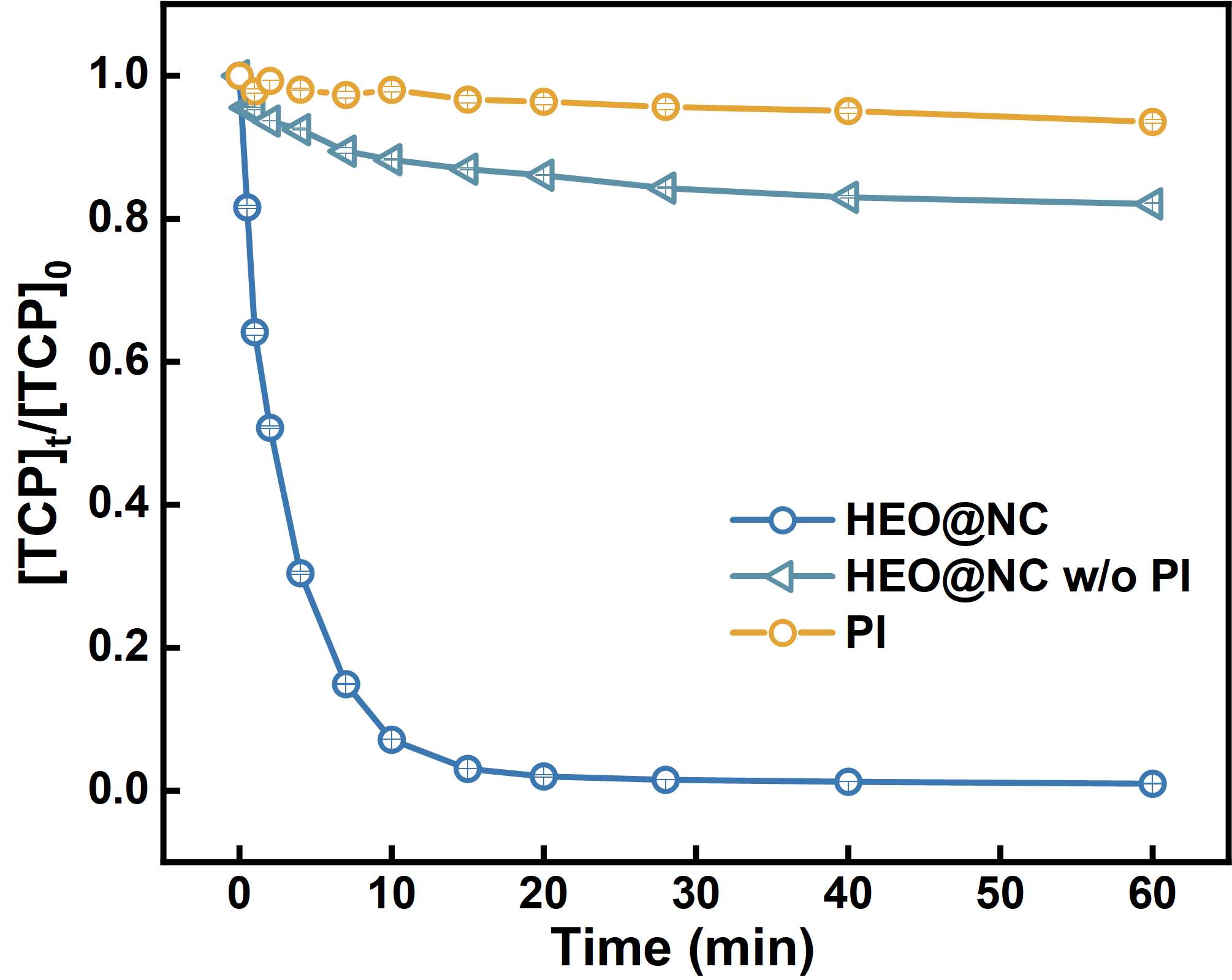


**Fig. S28 |** Removal of 2,4,6-TCP in various reaction system. Dosage: [TCP]_0_: 0.1 mM, reaction solution: 50 mL, catalyst: 0.1 g/L, reaction time: 60 min.


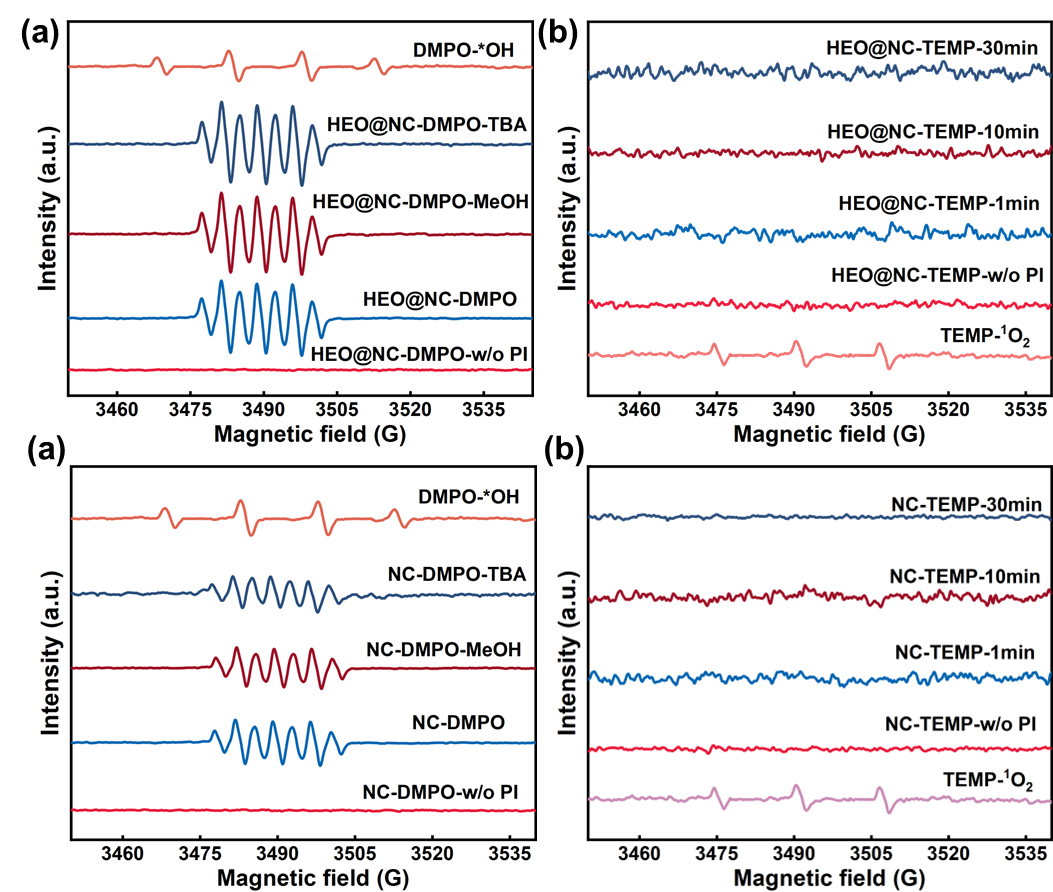


**Fig. S29 |** Influence of scavengers on the ESR signal of DMPO and TEMP adducts in the HEO@NC and NC system.

Electron spin resonance (ESR) failed to detect capture signals for ^•^OH, SO_4_^•‒^, and ^1^O_2_ in both the HEO@NC-PI and NG-PI systems. However, a seven-line spectrum corresponding to the oxidation product of DMPO, known as DMPOX, was observed. The addition of methanol (MeOH) or tert-butyl alcohol (TBA) as quenchers had minimal impact on the DMPOX signal intensity, indicating that ^•^OH/SO_4_^•‒^ plays a negligible role in the formation of DMPOX.


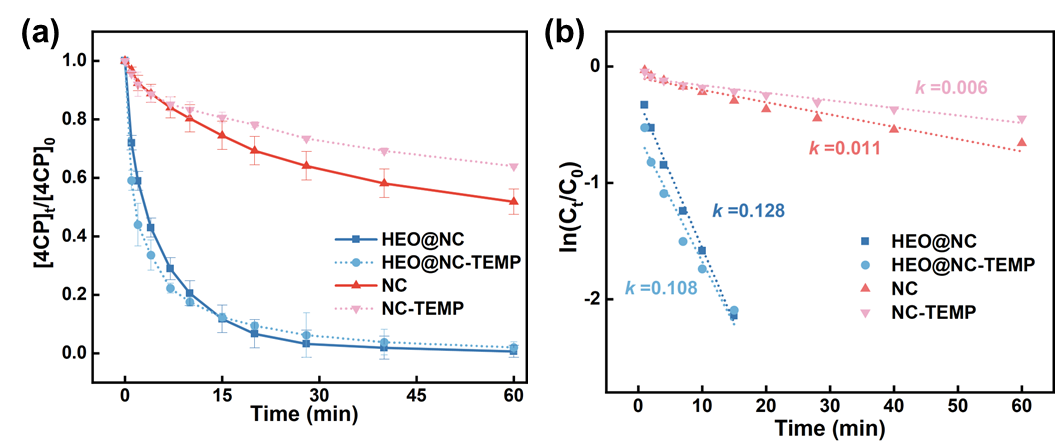


**Fig. S30 |** Quenching effects by TEMP. Dosage: [4CP]_0_: 0.1 mM, PI: 0.5 mM, [TEMP]_0_: 0.5 mM, [pH]_0_: 3.0, reaction solution: 50 mL, catalyst: 0.1 g/L.

The addition of 2,2,6,6-tetramethylpiperidine (TEMP) as a quencher for ^1^O_2_ had minimal impact on the decontamination performance of the HEO@NC and NC systems. Although there was some inhibition observed in the NC system, likely due to pH changes introduced by TEMP, these results still indicate that ^1^O_2_ has a negligible effect on the catalytic activity of both the HEO@NC and NC systems.


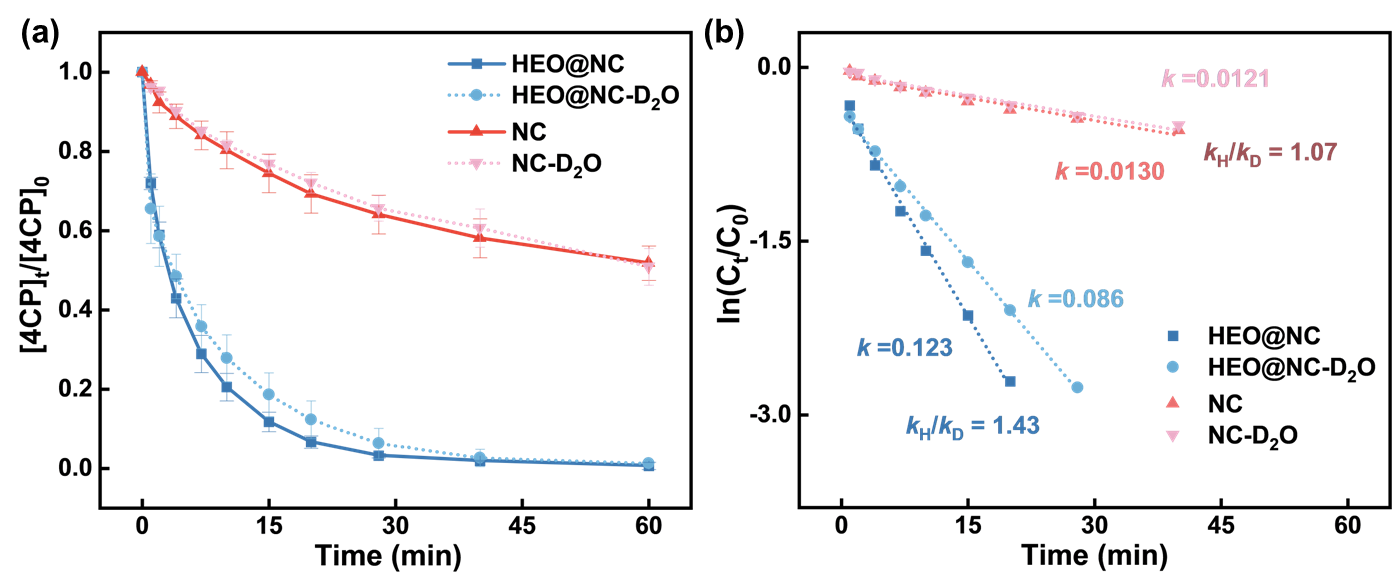


**Fig. S31 |** Degradation activity of HEO@NC and NC under H_2_O and D_2_O environments. Dosage: [4CP]_0_: 0.1 mM, PI: 0.5 mM, reaction solution: 50 mL, catalyst: 0.1 g/L, reaction time: 60 min.

Kinetic isotope effect (KIE) analysis further reveals that the HEO@NC–PI system exhibits a pronounced KIE value of approximately 1.43 (k_H_/k_D_ = 0.123/0.086), whereas the KIE value of the NC–PI system is only about 1.07 (k_H_/k_D_ = 0.013/0.0121). These results confirm the involvement of oxidative generation of organic radical intermediates in the rate-determining step and highlight the superior electron-transfer-induced bond activation capability of the HEO@NC system.


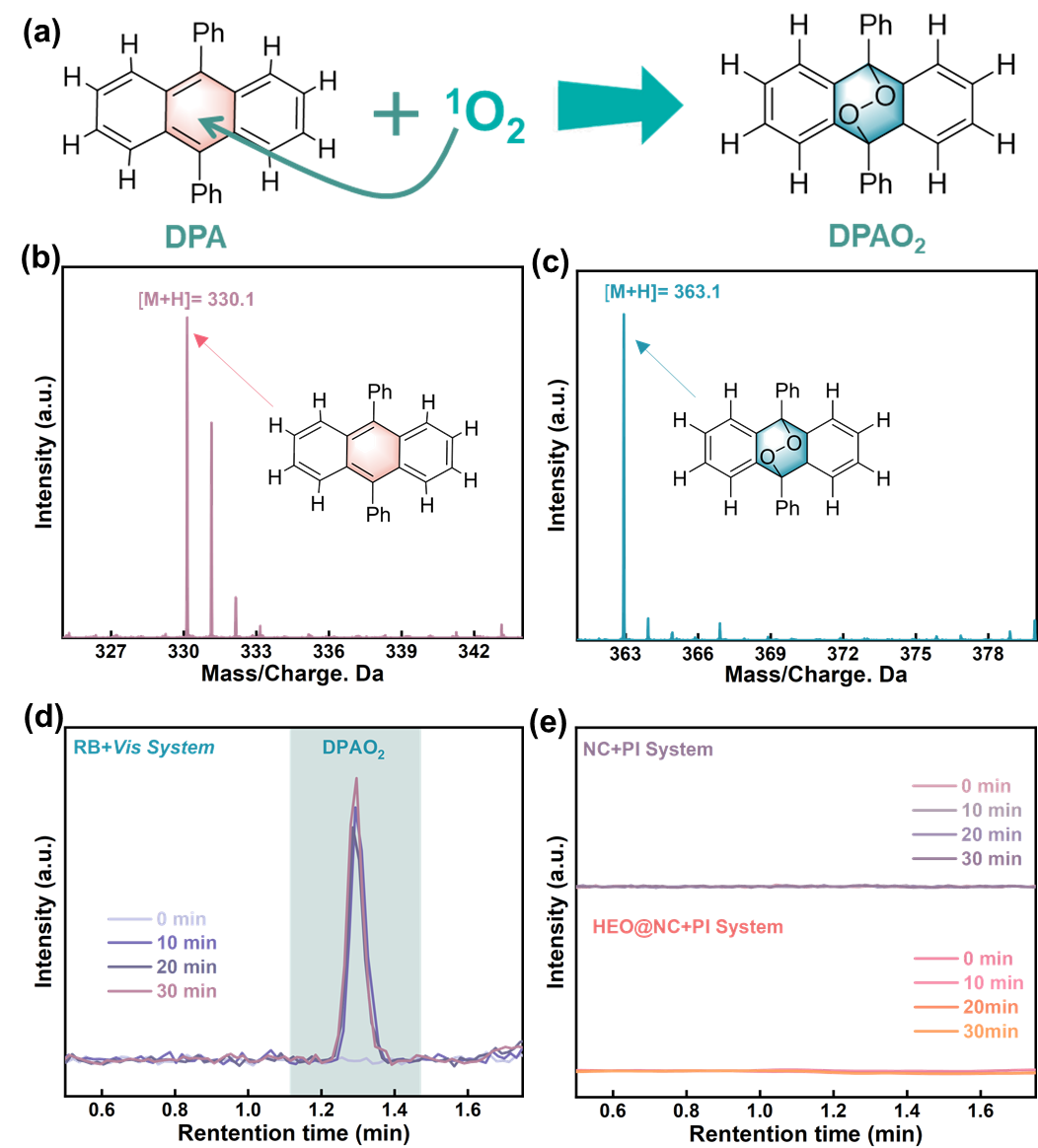


# Fig. S32 | (a) Schematic diagram of the principle of DPA probe and singlet oxygen generation for the target product DPAO_2_. the mass spectra of DPA (b) and DPAO_2_ (c). (d) the signal intensity of DPAO_2_ detected in the RB+*Vis* standard system. (e) the signal intensity of DPAO_2_ in HEO@NC-PI or NG-PI systems. Dosage: [DPA]_0_: 0.05mM, PI: 0.5 mM, reaction solution: 50 mL, catalyst: 0.1 g/L, reaction time: 30min.


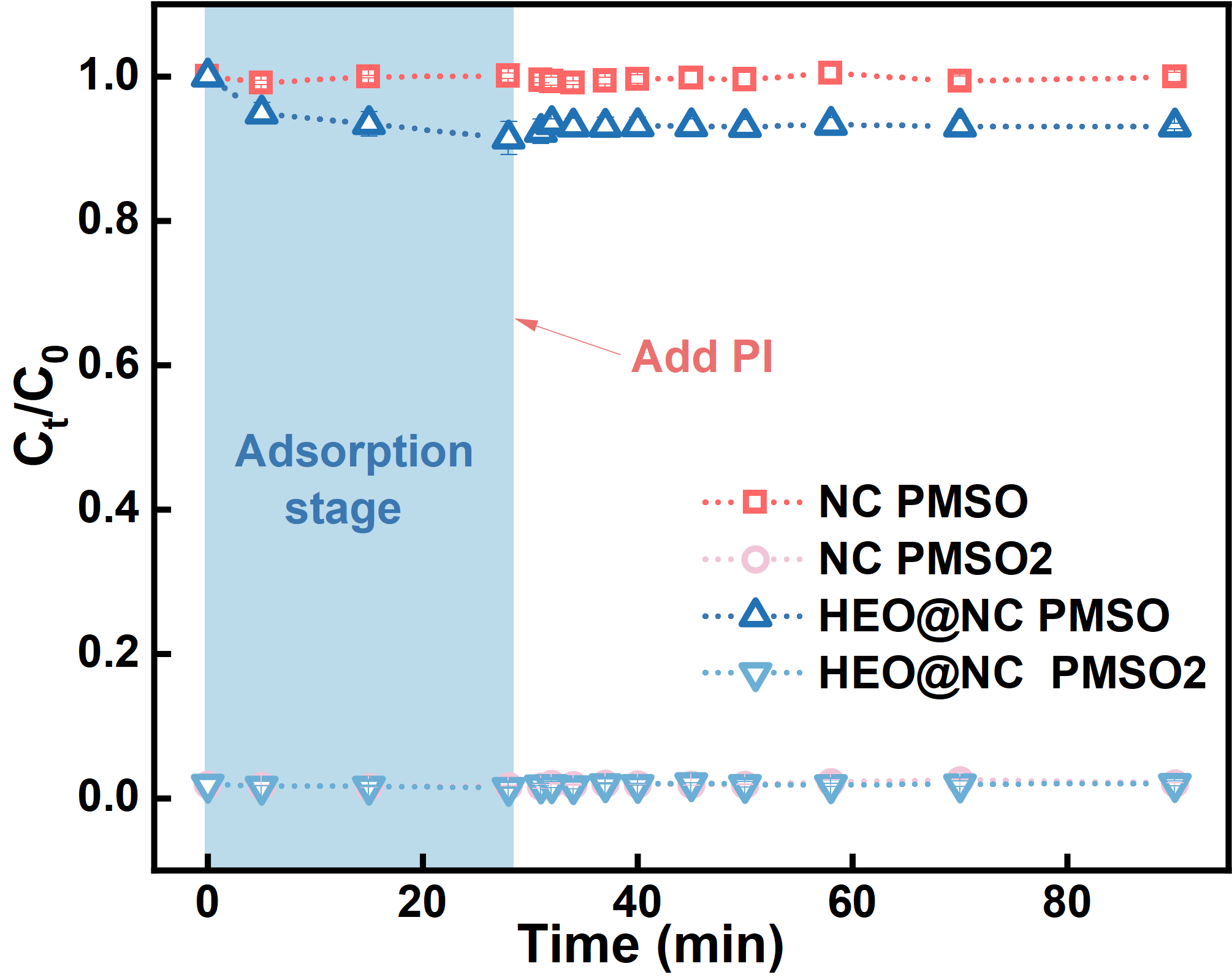


**Fig. S33 |** PMSO loss and PMSO₂ production in the HEO@NC/PI system. Dosage: [PMSO]_0_: 0.1 mM, PI: 0.5 mM, reaction solution: 50 mL, catalyst: 0.1 g/L.


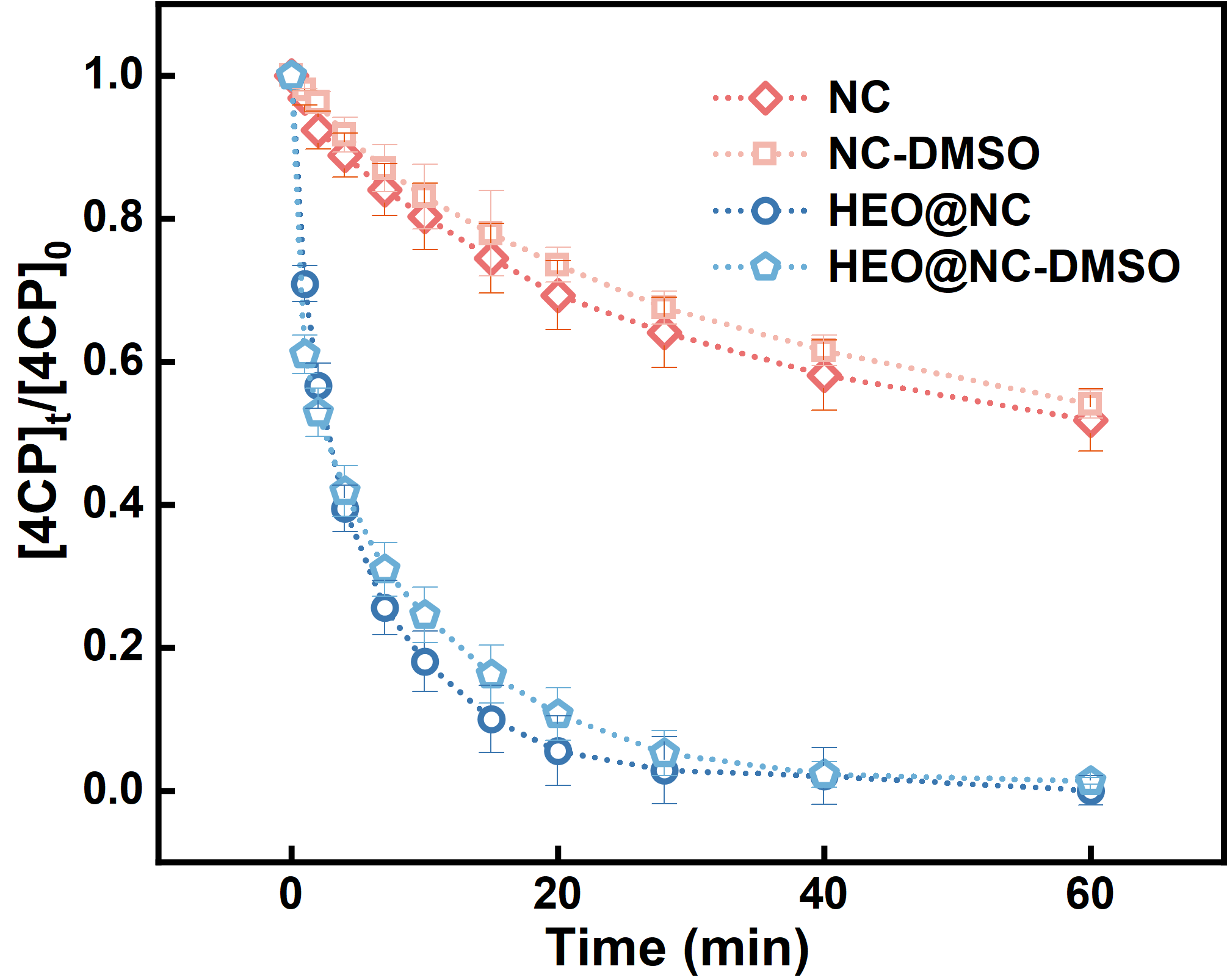


**Fig. S34 |** Quenching effects by DMSO. Dosage: [4CP]_0_: 0.1 mM, PI: 0.5 mM, [DMSO]_0_: 2.5 mM, reaction solution: 50 mL, catalyst: 0.1 g/L.


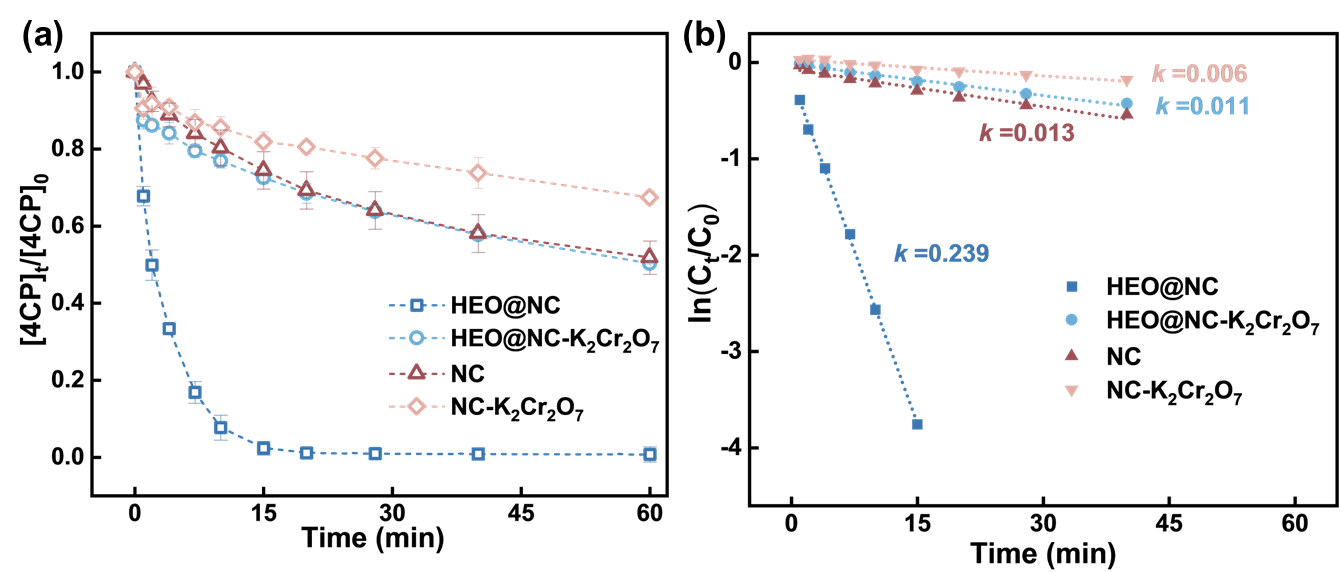


**Fig. S35 |** Quenching effects by K_2_Cr_2_O_7_. Dosage: [4CP]_0_: 0.1 mM, PI: 0.5 mM, [K_2_Cr_2_O_7_]_0_: 10 mM, reaction solution: 50 mL, catalyst: 0.1 g/L.

The addition of K_2_Cr_2_O_7_ as an electron transfer quencher led to pronounced suppression of the reaction in the HEO@NC-PI system, whereas only a modest inhibitory effect was observed in the NC-PI reference system. These results provide direct evidence for the dominant role of ETP and further demonstrate the enhanced interfacial electron-transfer capacity imparted by HEOs NPs.


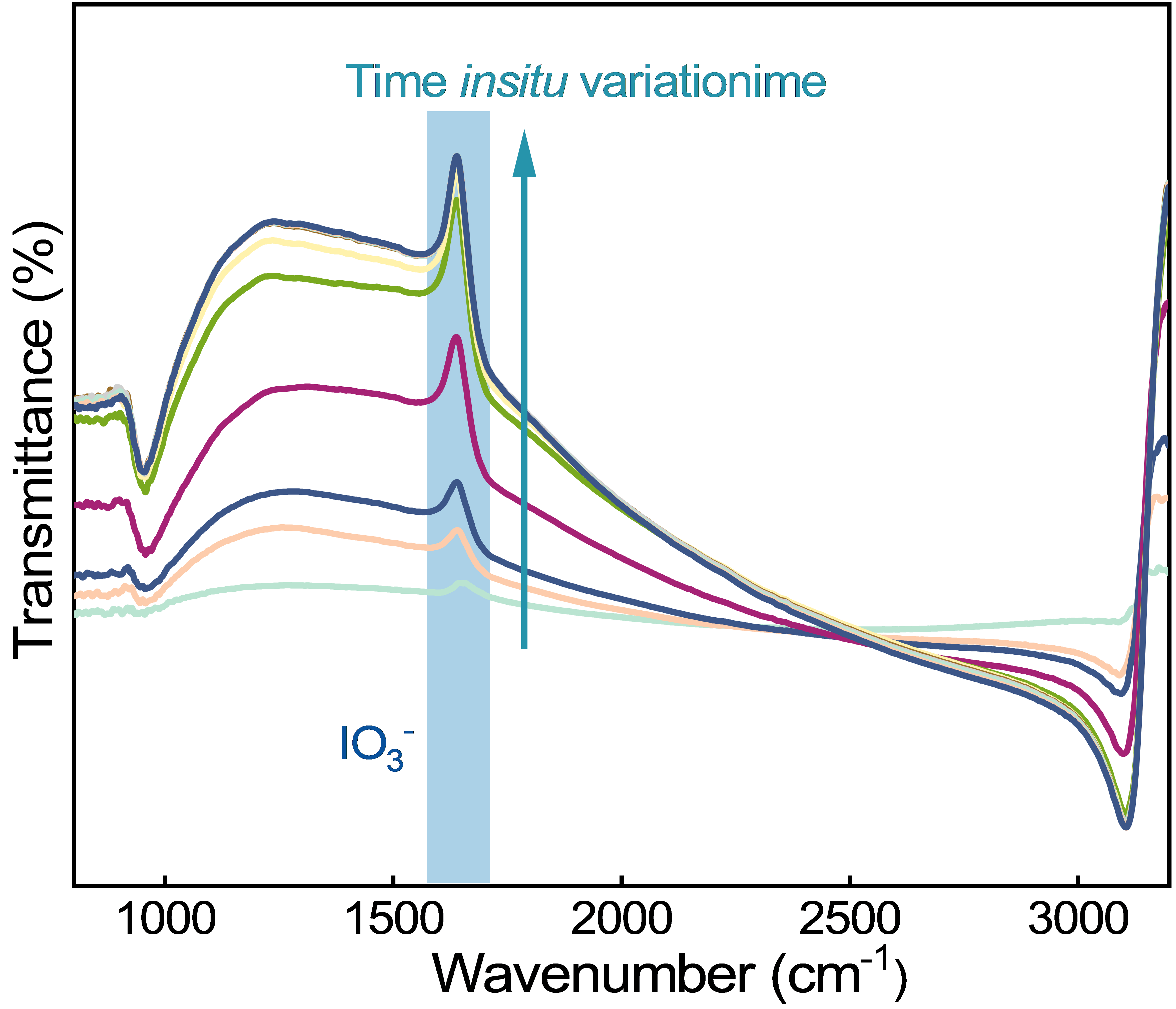


**Fig. S36 |** *In situ* SR-FTIR spectra of HEO@NC-PI system.

Further evidence comes from *in situ* synchrotron radiation Fourier-transform infrared (SR-FTIR) spectroscopy. Analysis of the HEO@NC and PI mixed suspension showed a gradual intensification of the stretching band at 1750 cm^-1^ over time. This observation indicates the formation of a PI* complex structure on the HEO@NC surface and confirms electron transfer between PI and HEO@NC. This mechanism is concurrently supported by CA and OCPT measurements, etc.


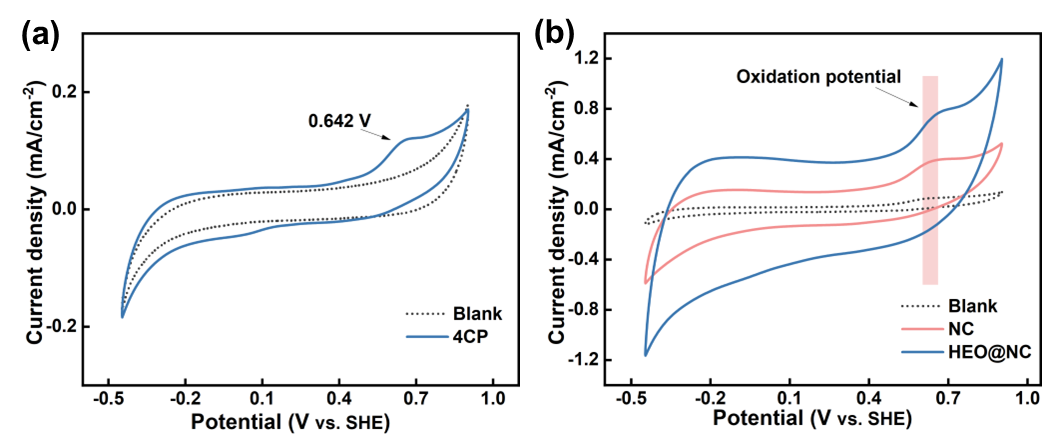


**Fig. S37 |** (a) The redox potential of 4CP in CV. (b) CV curve measurements on HEO@NC−GCE and NC−GCE electrode.

The oxidation peak of approximately 0.64 V observed for 4CP in the CV experiment corresponds to the complex potential of HEO@NC-PI* in the OCPT experiment, further confirming the electron transfer pathway between 4CP (electron donor) and PI (electron acceptor), mediated by HEO@NC and NC.

In the CV experiment, the current density generated by HEO@NC for the oxidation of 4CP is significantly higher than that of NC, once again demonstrating that HEO@NC exhibit a stronger electron transfer capability than NC during the oxidation of 4CP.


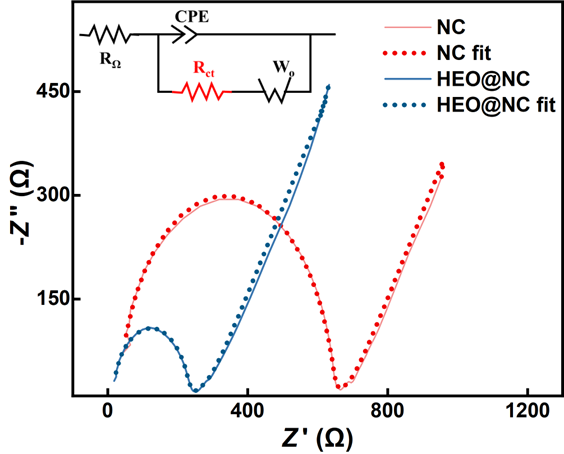


**Fig. S38 |** Nyquist plots and fitting calculations of internal resistance for HEO@NC and NC.


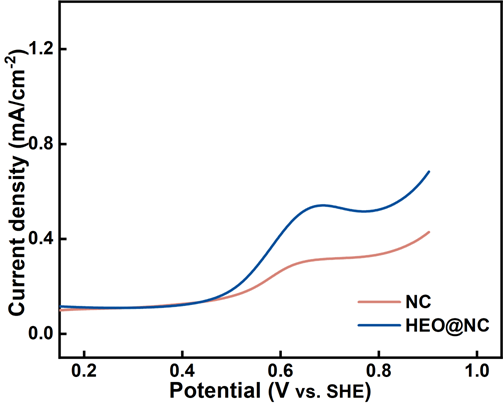


**Fig. S39 |** LSV on the HEO@NC and NC electrodes in 4CP solutions.


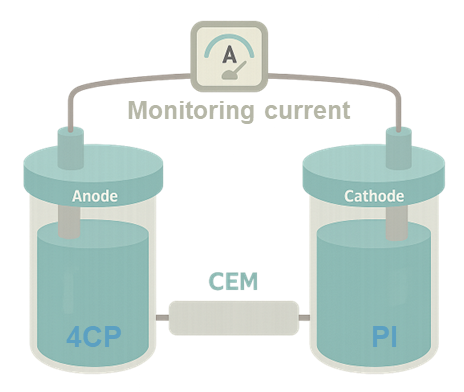


**Fig. S40 |** The schematic diagram of the galvanic reactor.

The anode and cathode chambers are separated by a cation exchange membrane (CEM), with electrodes on both sides connected through a high-precision ammeter to monitor current changes. The electrode potential within the PI chamber was notably higher than that in the 4CP chamber, indicating that the PI chamber served as the cathode chamber, while the 4CP solution acted as the anode chamber.


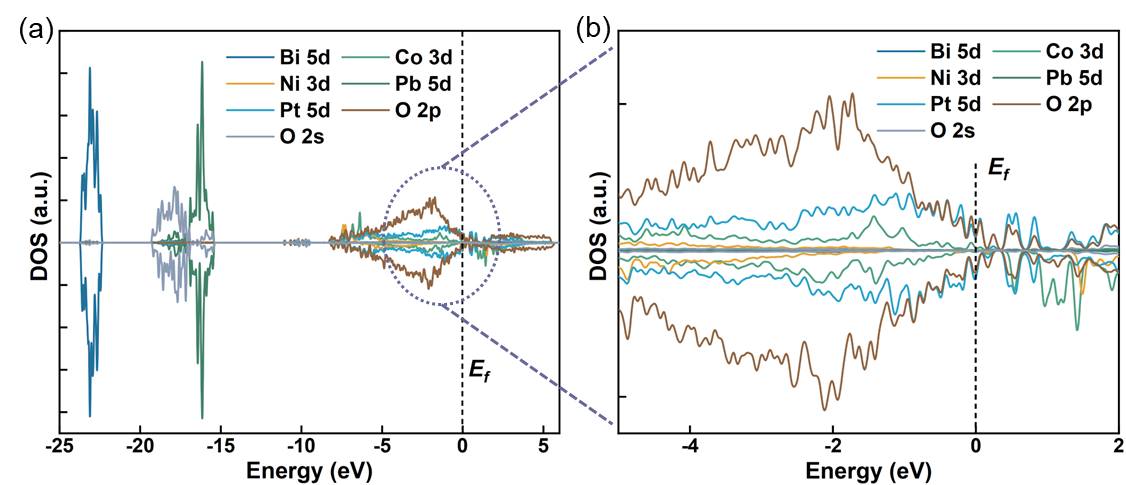


**Fig. S41 |** The PDOS of HEO@NC.

The Ni 3d, Co 3d, and Pt 5d orbitals exhibit high electronic density of states (DOS) near the Fermi level (E_f_), indicating their roles as primary electron donors within the system. Among them, the Pt 5d orbitals, with a continuous distribution of unoccupied and partially occupied states, create low-barrier electron transport channels that dominate the charge migration process, while the Ni/Co d orbitals contribute to surface adsorption through localized electrons. The d orbitals of Bi and Pb undergo orbital hybridization under multi-metal synergy, broadening the energy band range, promoting charge delocalization, and optimizing charge transport pathways. The oxygen 2s orbitals (–18 eV to –16 eV) and 2p orbitals (–5 eV to –1 eV) show pronounced DOS peaks, reflecting their distinct bonding characteristics: the 2s orbitals help stabilize the coordination of oxygen, whereas the 2p orbitals bridge metal d orbitals via p–d hybridization, thereby enhancing surface stability and facilitating charge transfer. In summary, the synergistic interactions among multiple metal components regulate electronic state distribution, optimize charge transport kinetics, and enhance surface stabilization, ultimately contributing to the overall improvement of catalytic activity.


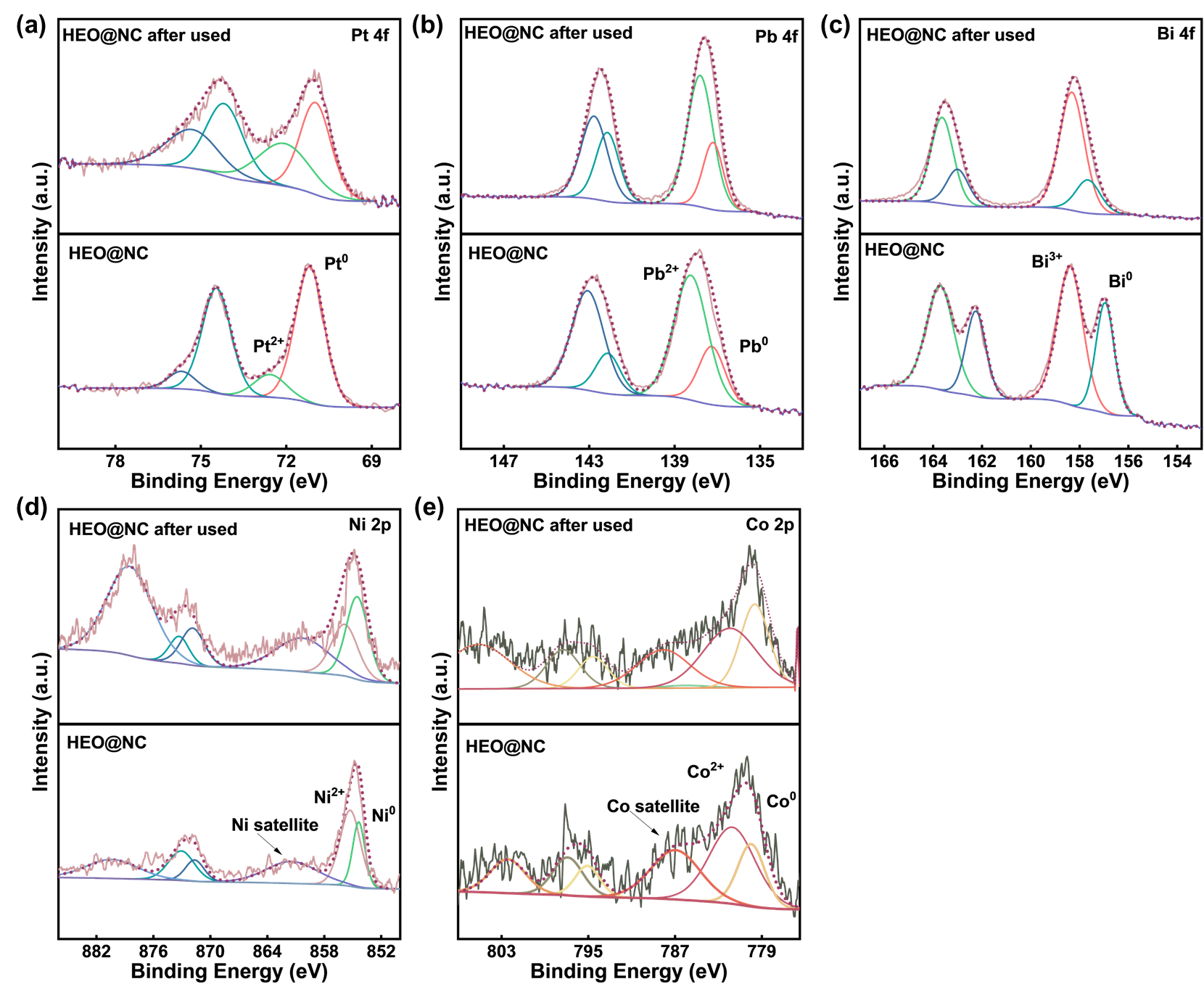


**Fig. S42 |** Comparative XPS analysis of the HEO@NC catalyst after use: (a) Pt 4f, (b) Pb 4f, (c) Bi 4f, (d) Ni 2p, and (e) Co 2p.

Comparative quasi-in situ XPS analysis before and after the reaction reveals pronounced metal-specific shifts in binding energy. The Pt 4f peaks exhibit a negative overall shift, indicating increased electron density and partial reduction. In contrast, Ni 2p and Co 2p show positive binding energy shifts, suggesting that they primarily function as adsorption sites and electron donors and undergo partial oxidation. Pb 4f displays only a slight negative shift, implying limited changes in its oxidation state, whereas Bi 4f exhibits a pronounced positive shift, reflecting significant modulation of its electronic structure. These experimental observations are in excellent agreement with the theoretical calculations.


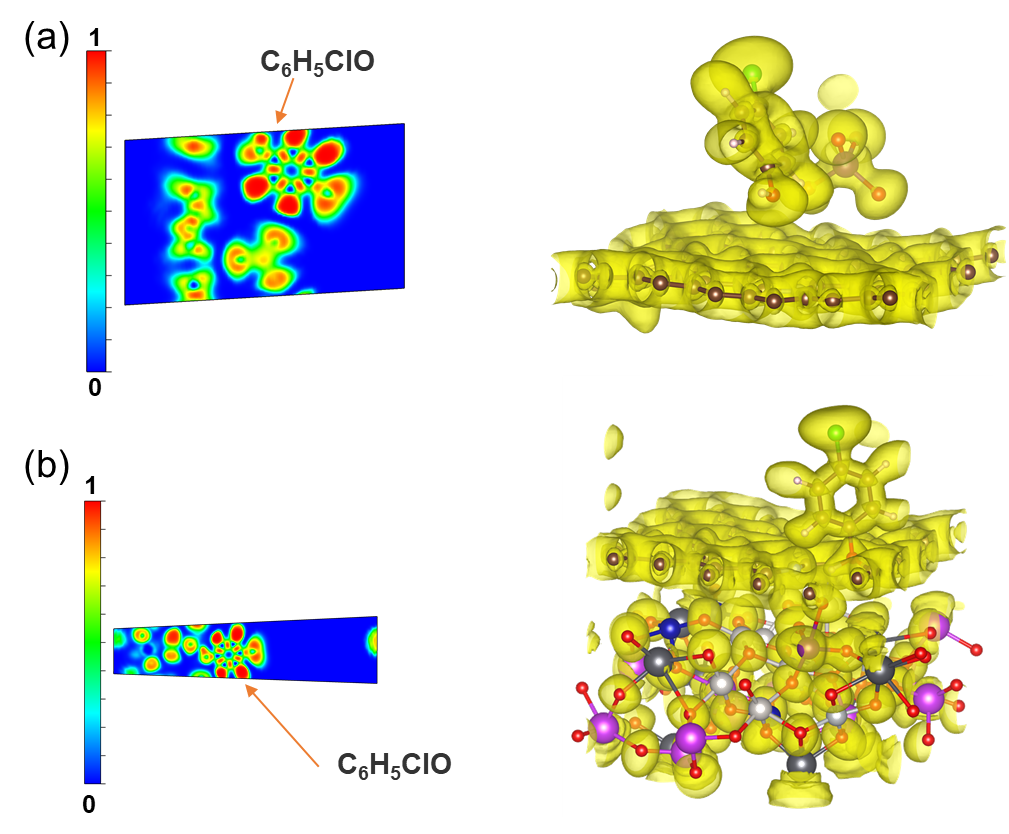


**Fig. S43 |** Charge localization function diagram for 4CP adsorption on NC (a) and HEO@NC (b).


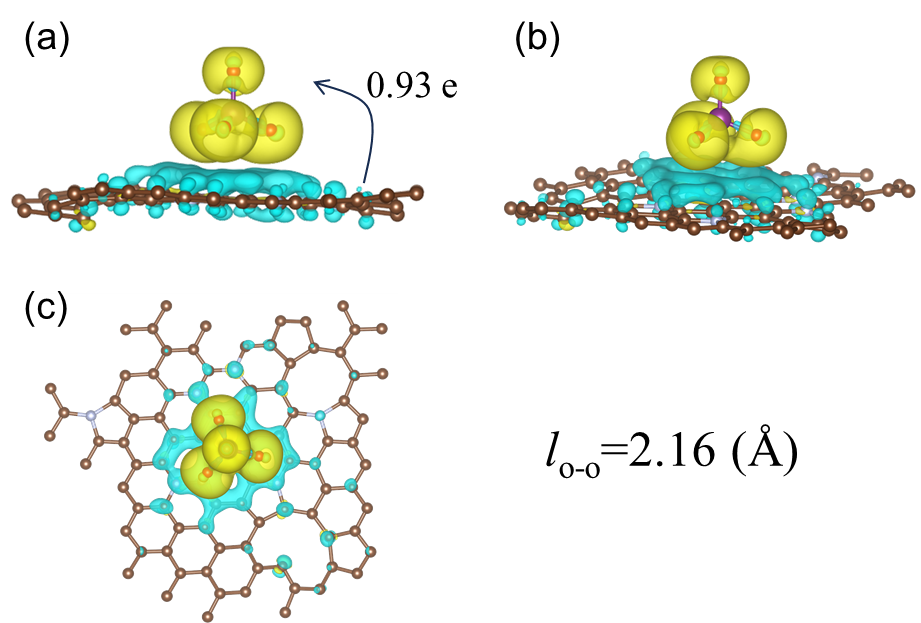


**Fig. S44 |** Electron density difference and the corresponding Bader charges (Q_e_) after PI adsorption on the NC.

Side view and top view of the charge density difference of NC-PI with an isosurface of 3*10^−3^ e‧Å^−3^. (The charge accumulation is shown as the yellow region, and the charge depletion is shown as the cyan region.).


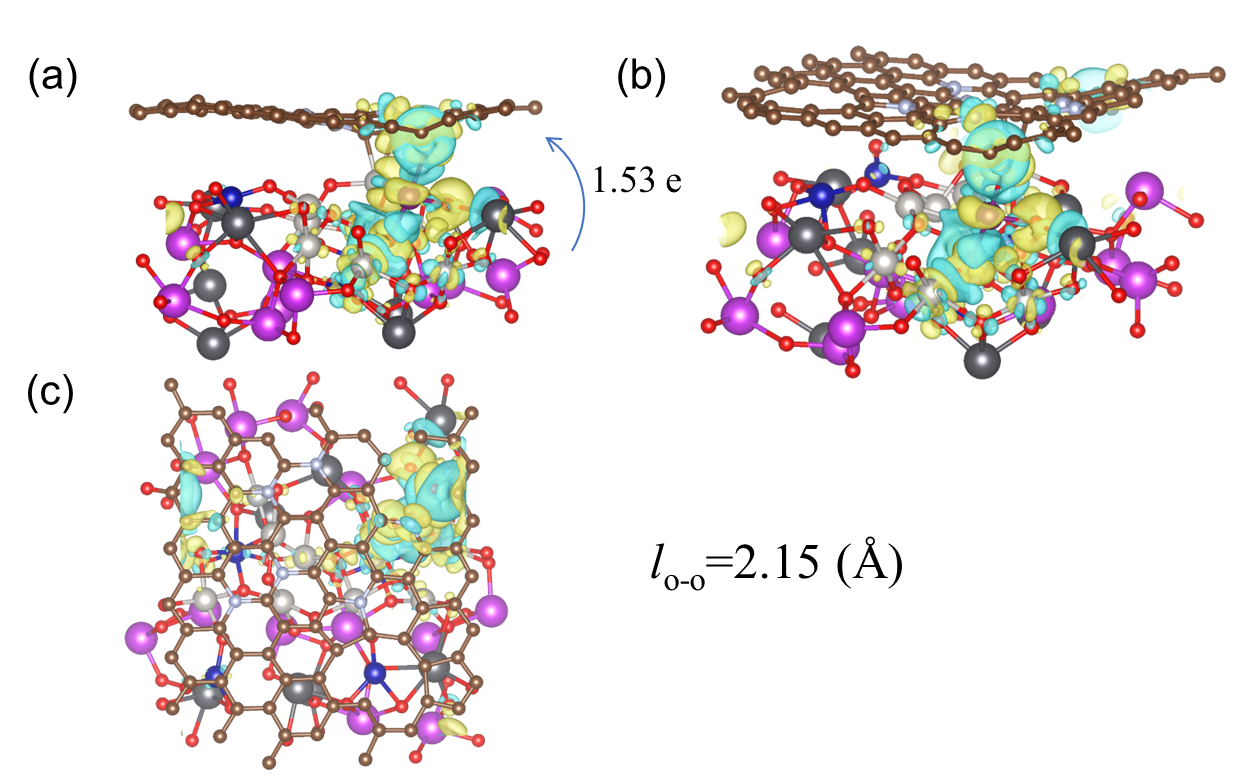


**Fig. S45 |** Electron density difference and the corresponding Bader charges (Q_e_) after PI adsorption on the HEO@NC.

Side view and top view of the charge density difference of HEO@NC-PI with an isosurface of 3*10^−3^ e‧Å^−3^. (The charge accumulation is shown as the yellow region, and the charge depletion is shown as the cyan region.).


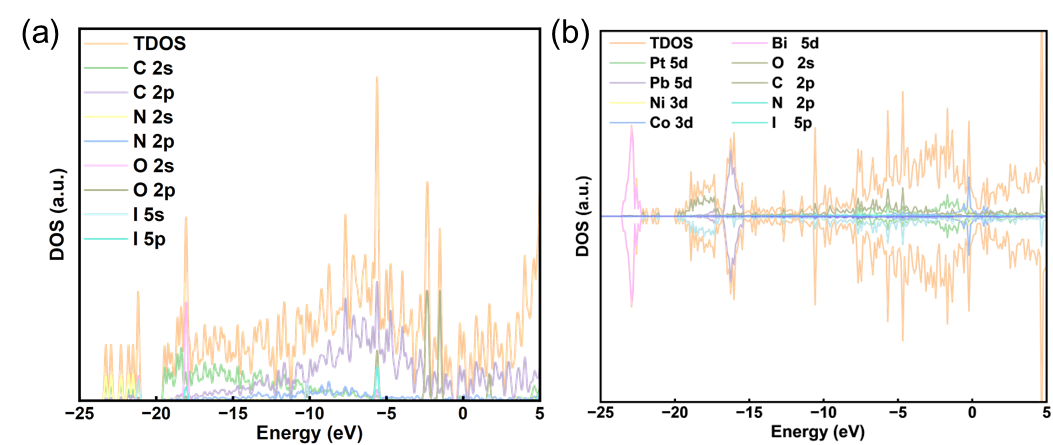


**Fig. S46 |** The PDOS of HEO@NC—PI and NC—PI.

In the NC-PI system, the energy levels of I are primarily composed of the 5s and 5p orbitals, with the 5p orbital playing a dominant role. Analysis of the density of states (DOS) reveals a complete overlap between the I 5p orbital and the O 2s and 2p orbitals at -5.61 eV, indicating a strong orbital hybridization between I and O at this position. However, when PI is adsorbed on the HEOs surface, the orbital coupling position of the I-O bond shifts towards a lower (more negative) energy level. Additionally, the primary orbital involved in coupling with O changes from the I 5p orbital to the I 5s orbital. The shift of the orbital coupling to a lower energy level generally indicates enhanced system stability, suggesting a strong interaction between the I-O bond and the HEOs surface. This leads to a reduction in the binding energy of the I-O bond, resulting in increased stability of the I-O bond.

Moreover, the enhanced coupling between the I 5s orbital and the O orbital implies significant charge transfer between I and O. The involvement of the 5s orbital typically indicates stronger coupling between the low-energy orbitals of I and the O atom, which may alter the electron distribution of I, leading to an electron density shift towards the O atom. This stabilizes the adsorbed state structure, enabling PI to exist more stably on the HEOs surface and facilitating subsequent oxidation reactions.

The 3d orbitals of Ni are closer to the Fermi level, making them frontier orbitals that can more easily interact with the O atoms of PI. This interaction helps stabilize PI on the HEO surface, thereby facilitating the oxidation of pollutants by PI.


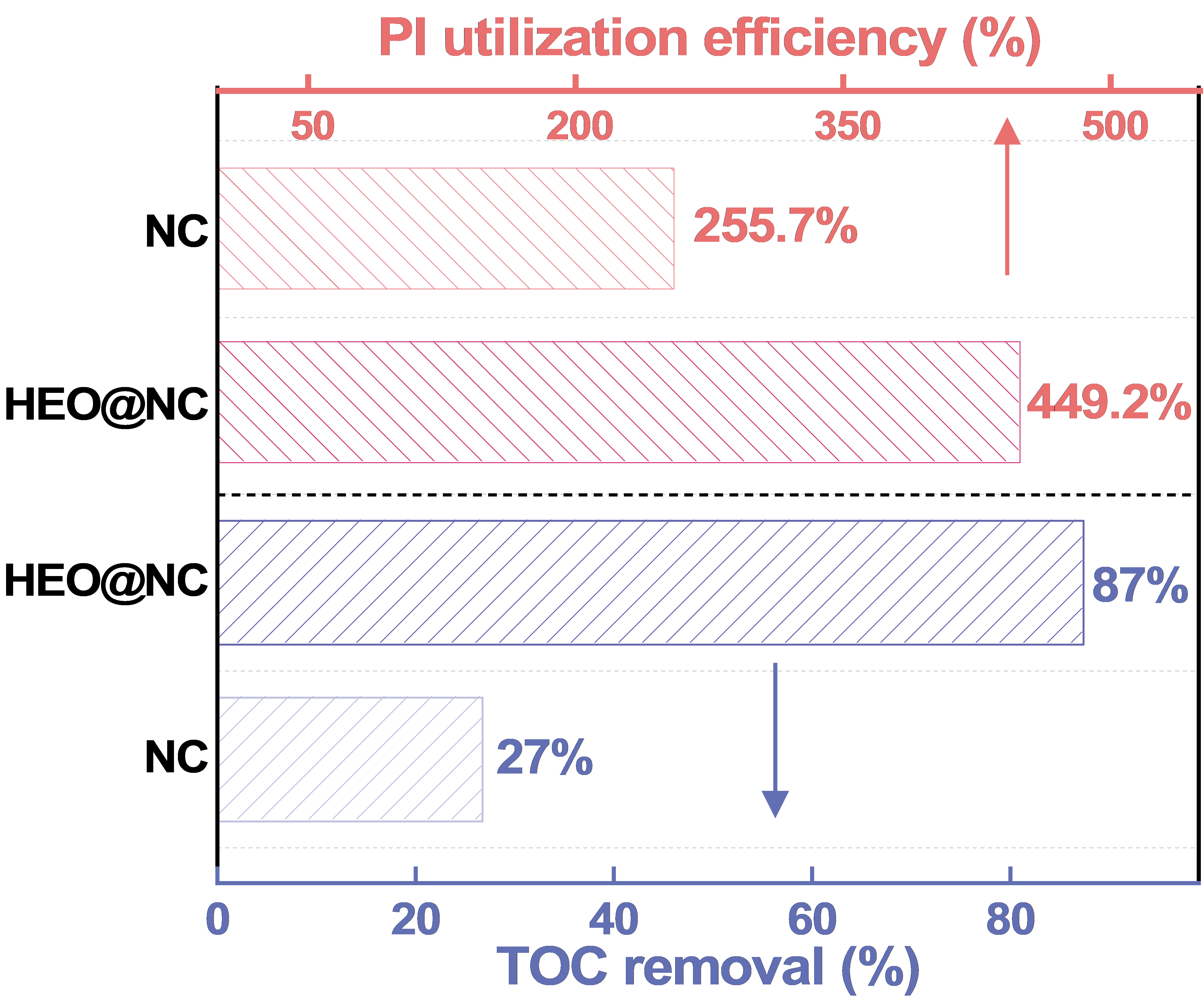


**Fig. S47 |** HEO@NC-PI and NC-PI aqueous solution systems final TOC removal efficiency, and PI utilization.

The TOC removal efficiency of HEO@NC-PI and NC-PI systems was analyzed to elucidate the observed discrepancy in PI equivalents during 4CP elimination (Table S8). The results demonstrated that the theoretical maximum electron equivalents derived from real PI consumption (0.254 mM) were substantially lower than the actual electron equivalents lost during 4CP mineralization (2.272 mM). This discrepancy can be attributed to the selective oxidation of 4CP through single-electron transfer within the polymerization pathway (PI:4CP molar ratio of 1:2), rather than mineralization processes requiring significantly higher theoretical PI consumption (PI:4CP molar ratio of 13:1). Benefiting from this oxidation mechanism, the HEO@NC-PI system exhibited superior PI utilization efficiency (449.2%) compared to the NC-PI system (255.7%), while demonstrating substantially reduced oxidant consumption in practical applications relative to homogeneous mineralization catalysis.


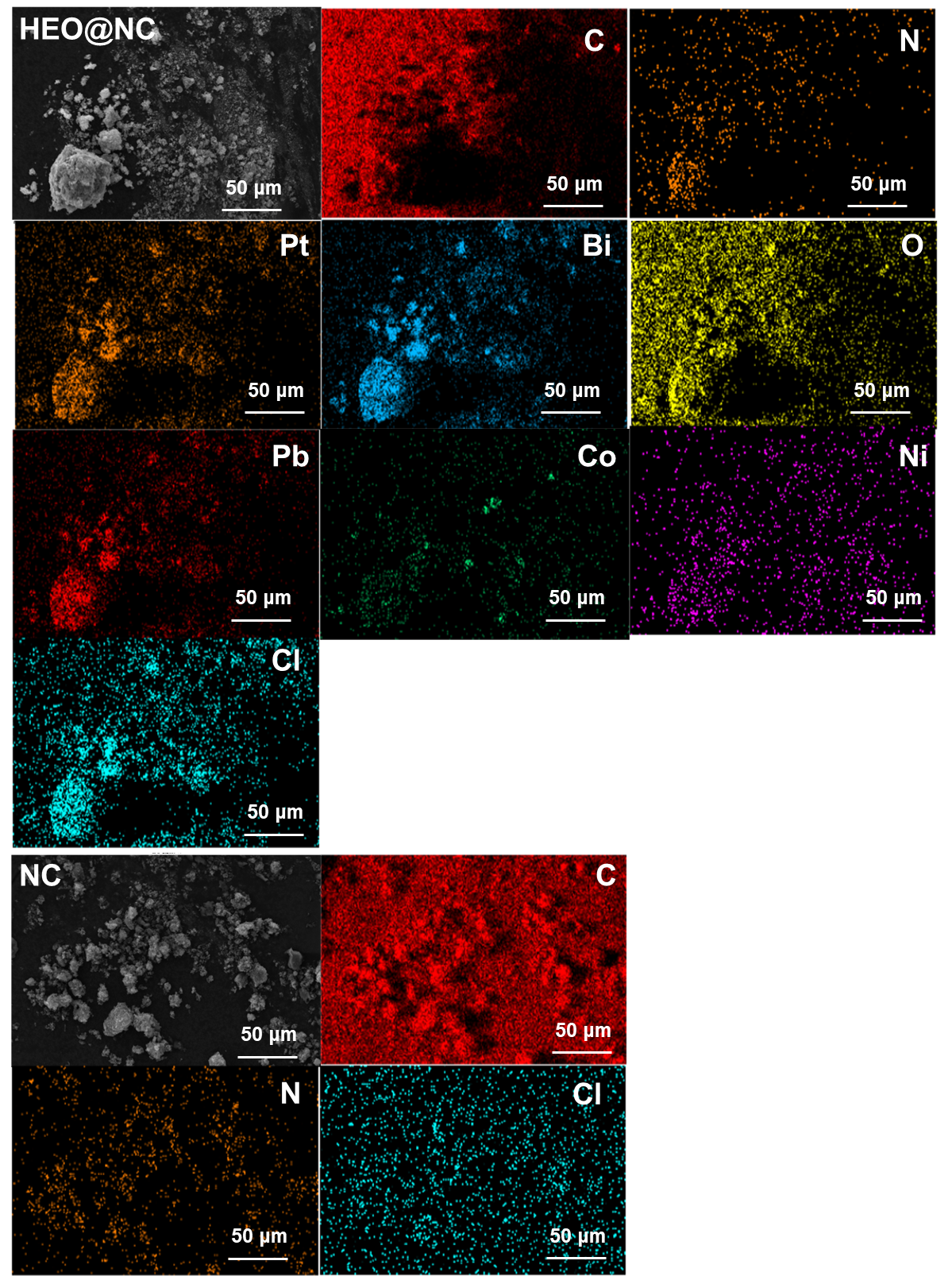


**Fig. S48 |** EDS mapping images show the uniform dispersion of C, N, Pb, Pb, Bi, Ni, Co, and O elements in a HEO@NC.

Energy-dispersive spectroscopy (EDS) elemental mapping reveals a notably higher Cl signal intensity on the surface of HEO@NC than on NC, suggesting enhanced accumulation or adsorption of chlorine-containing species on the HEO@NC catalyst. These findings suggest that instead of being fully decomposed, the pollutants are likely transferred and accumulate on the catalyst surface through polymerization. HEO@NC exhibits a greater capability to facilitate the polymerization and transformation of these pollutants.


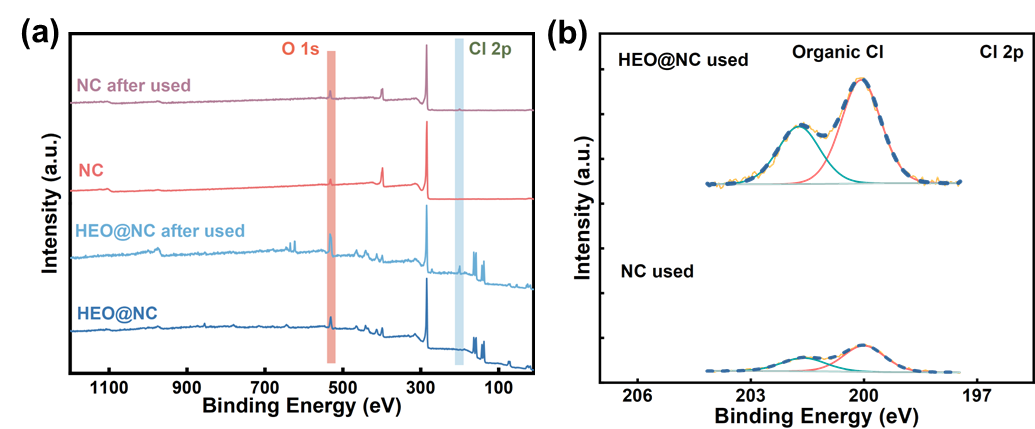


**Fig. S49 |** XPS spectra comparisons: (a) Survey and (b) Cl 2p spectra of HEO@NC and NC, as well as the spectra after use.

The XPS survey and Cl 2p spectra of HEO@NC and NC show that the Cl signal intensity on the surface of HEO@NC is significantly higher than that of NC, indicating an enhanced accumulation of chlorine-containing products on the HEO@NC catalyst. These findings suggest that HEO@NC exhibits a stronger ability to promote the polymerization and conversion of these pollutants, leading to more accumulation of chlorine-containing polymeric products on the catalyst surface.


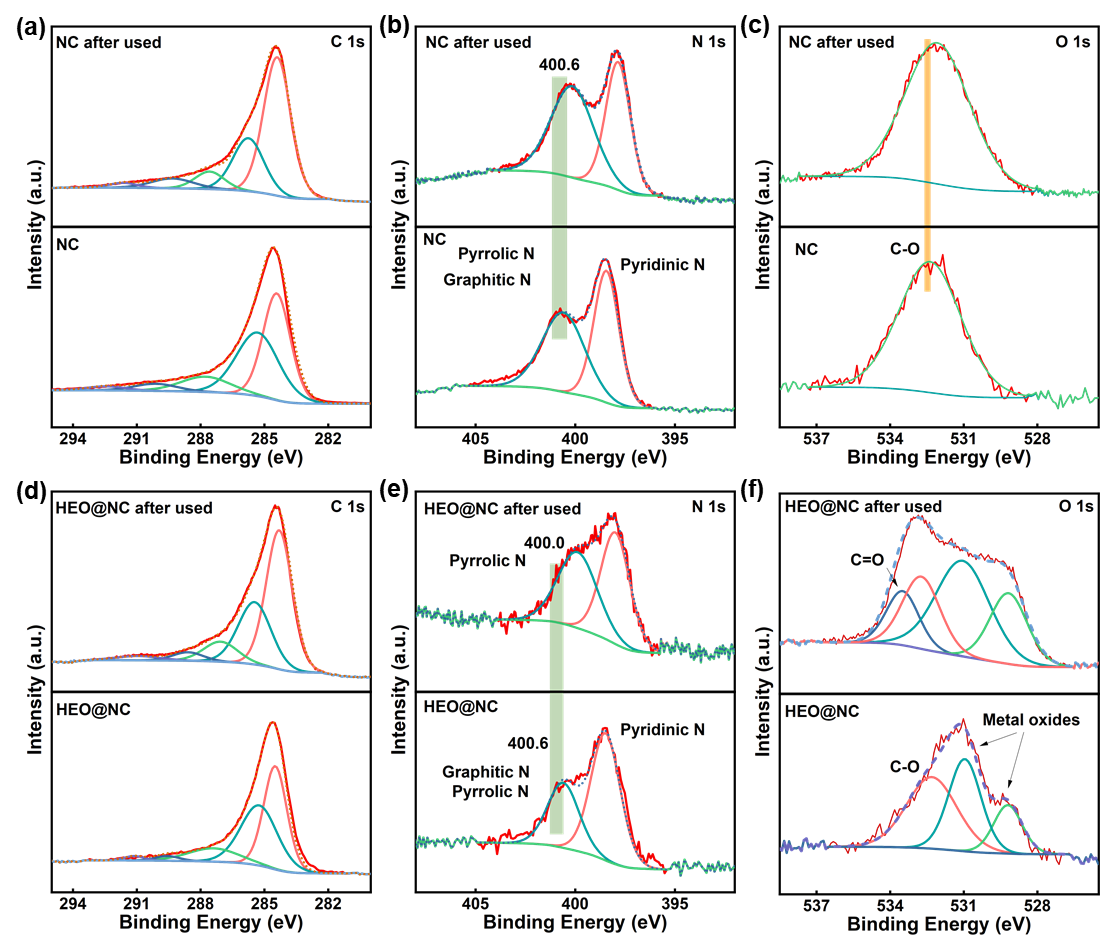


**Fig. S50 |** Comparison of (a) C 1s, (b) N 1s, and (c) O 1s XPS spectra of HEO@NC and NC after use.

According to literature^7^, graphitic nitrogen defect sites in nitrogen-doped carbon exhibit the strongest activation ability for peroxides. Based on the N 1s spectra, the increased prominence of pyrrolic N after 4CP treatment may be due to the accumulation of a large amount of chlorine-containing polymeric products at the active sites. Moreover, the O 1s spectra indicate that the HEO@NC catalyst's stronger oxidative capacity leads to the conversion of phenolic hydroxyl groups into quinone intermediates, as evidenced by the appearance of C=O signals.


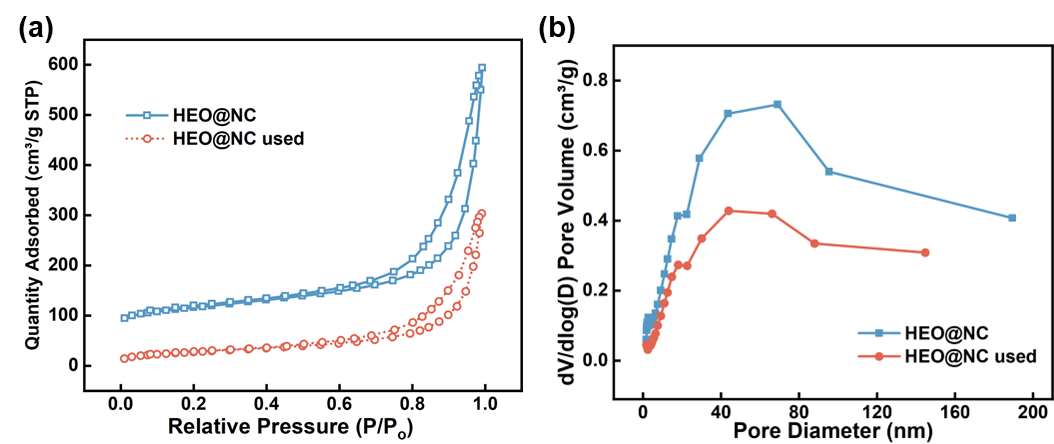


**Fig. S51 |** Comparison of (a) N_2_ adsorption and desorption isotherms and (b) pore size distribution spectra of HEO@NC after use.

The N_2_ adsorption-desorption isotherms and pore size analysis of HEO@NC before and after 4CP treatment show a significant decrease in surface area and pore volume, indicating substantial accumulation of chlorine-containing polymeric products within the pores of the HEO@NC catalyst.


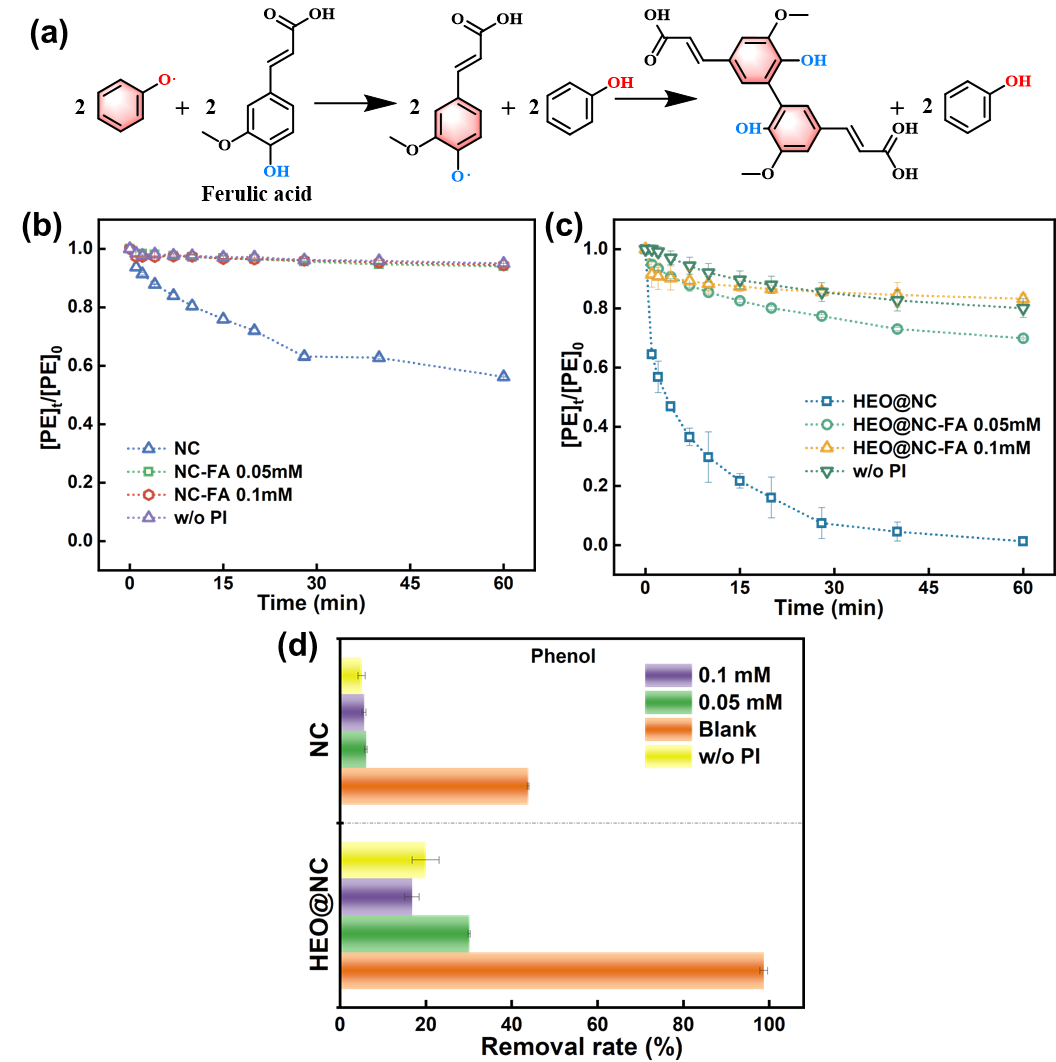


**Fig. S52 |** (a) The schematic illustration of phenoxyl radicals quenching by FA. The effect of FA on phenol removal by NC-PI (b) and HEO@NC (c) systems. (d) The effect of different concentrations of FA on the phenol removal rate in the NC-PI and HEO@NC-PI systems. Error bars represent the standard deviation, obtained by repeating the experiment three times. Dosage: [phenol]_0_: 0.1 mM, PI: 0.5 mM, reaction solution: 50 mL, catalyst: 0.1 g/L.

Previous studies have shown that FA can convert phenoxy radicals back into their redox-inert phenol precursors, making it a highly specific scavenger of phenoxy radicals. The results indicate that approximately 0.05 mM FA completely inhibits phenol removal in the NC-PI system, whereas in the HEO@NC-PI system, the inhibition effect increases with higher FA concentrations, eventually leading to complete suppression at concentrations above 0.1 mM. These findings strongly suggest that the higher PI* complexation potential in the HEO@NC-PI system promotes the formation of more phenoxy radicals during pollutant polymerization compared to the NC-PI system.


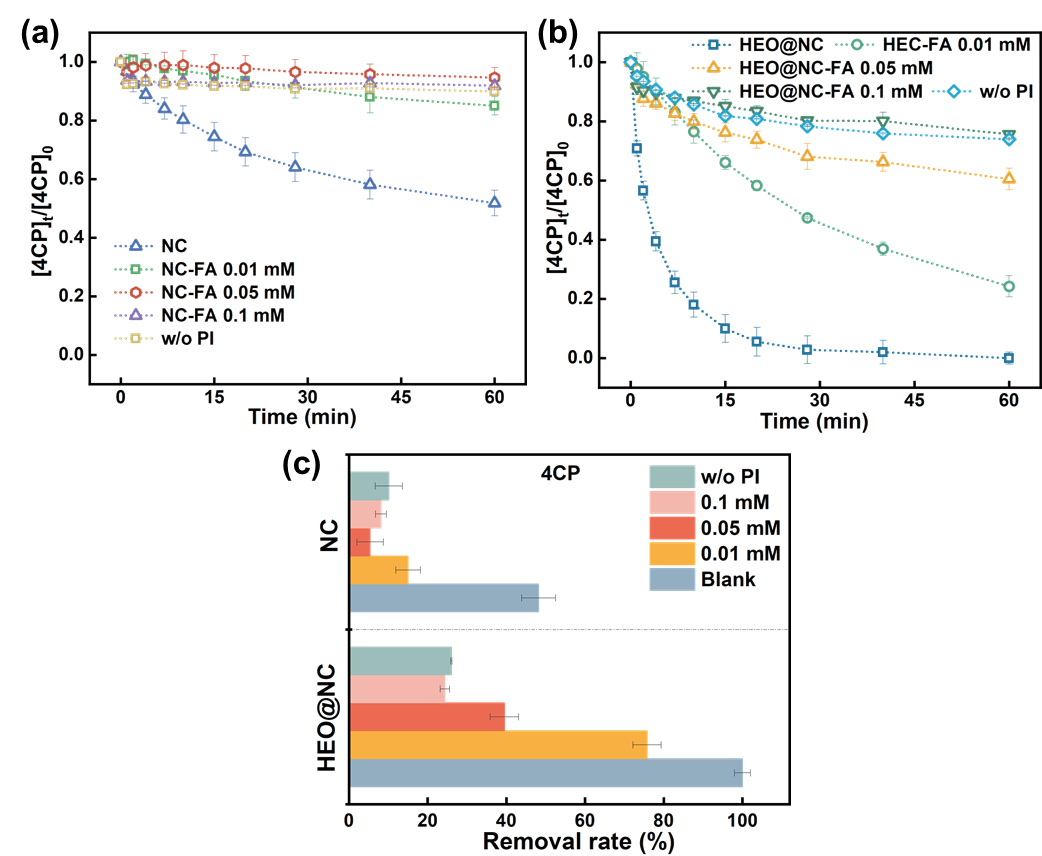


**Fig. S53 |** The effect of FA on 4CP removal by NC-PI (a) and HEO@NC-PI (b) systems. (c) The effect of different concentrations of FA on the 4CP removal rate in the NC-PI and HEO@NC-PI systems. Dosage: [4CP]_0_: 0.1 mM, PI: 0.5 mM, reaction solution: 50 mL, catalyst: 0.1 g/L.


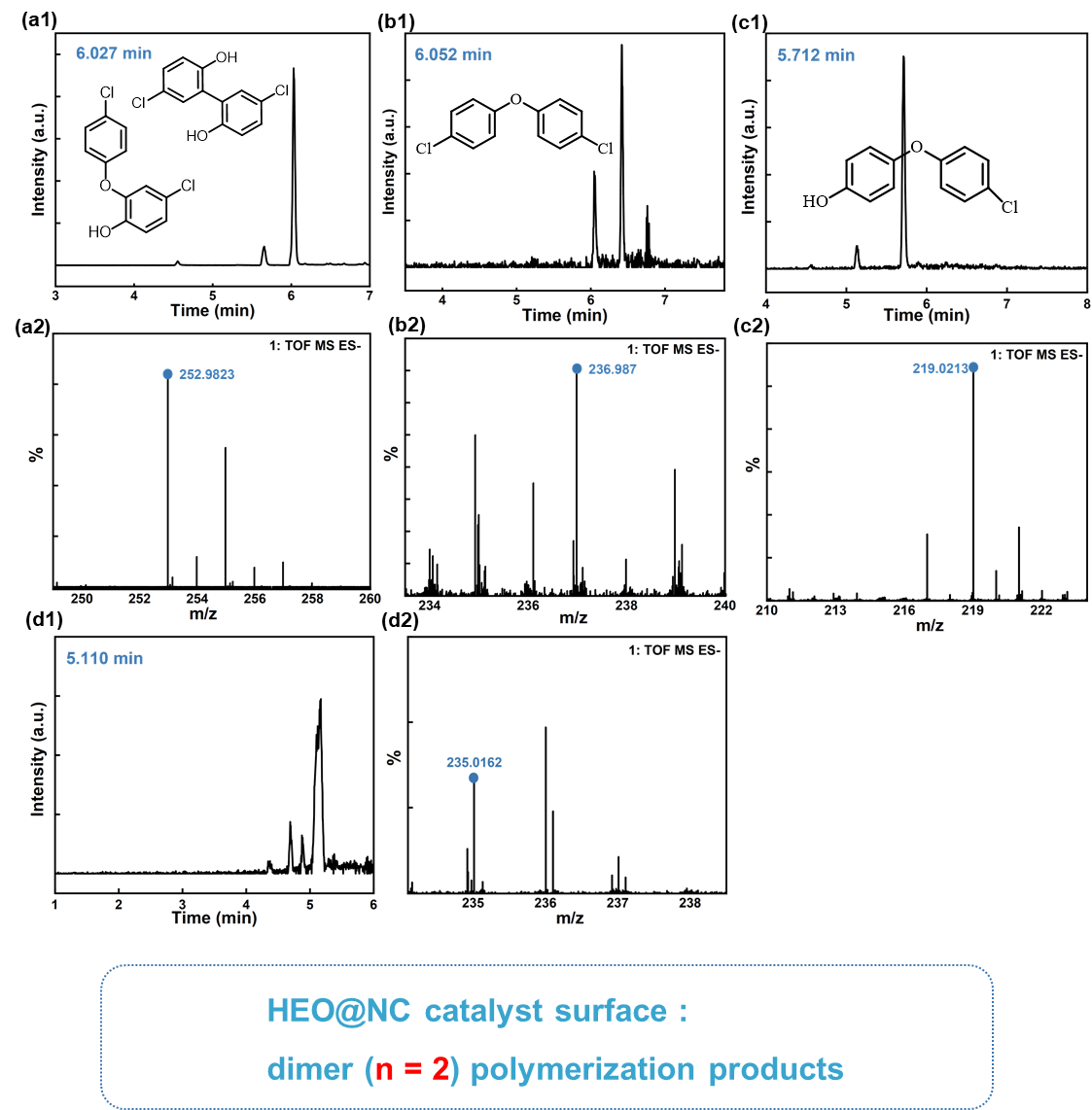


**Fig. S54 |** UPLC–QTOF-MS chromatograms of 4CP oxidation polymerization products in the HEO@NC catalyst surface, and their corresponding molecular ion mass spectra of the chromatographic peaks.


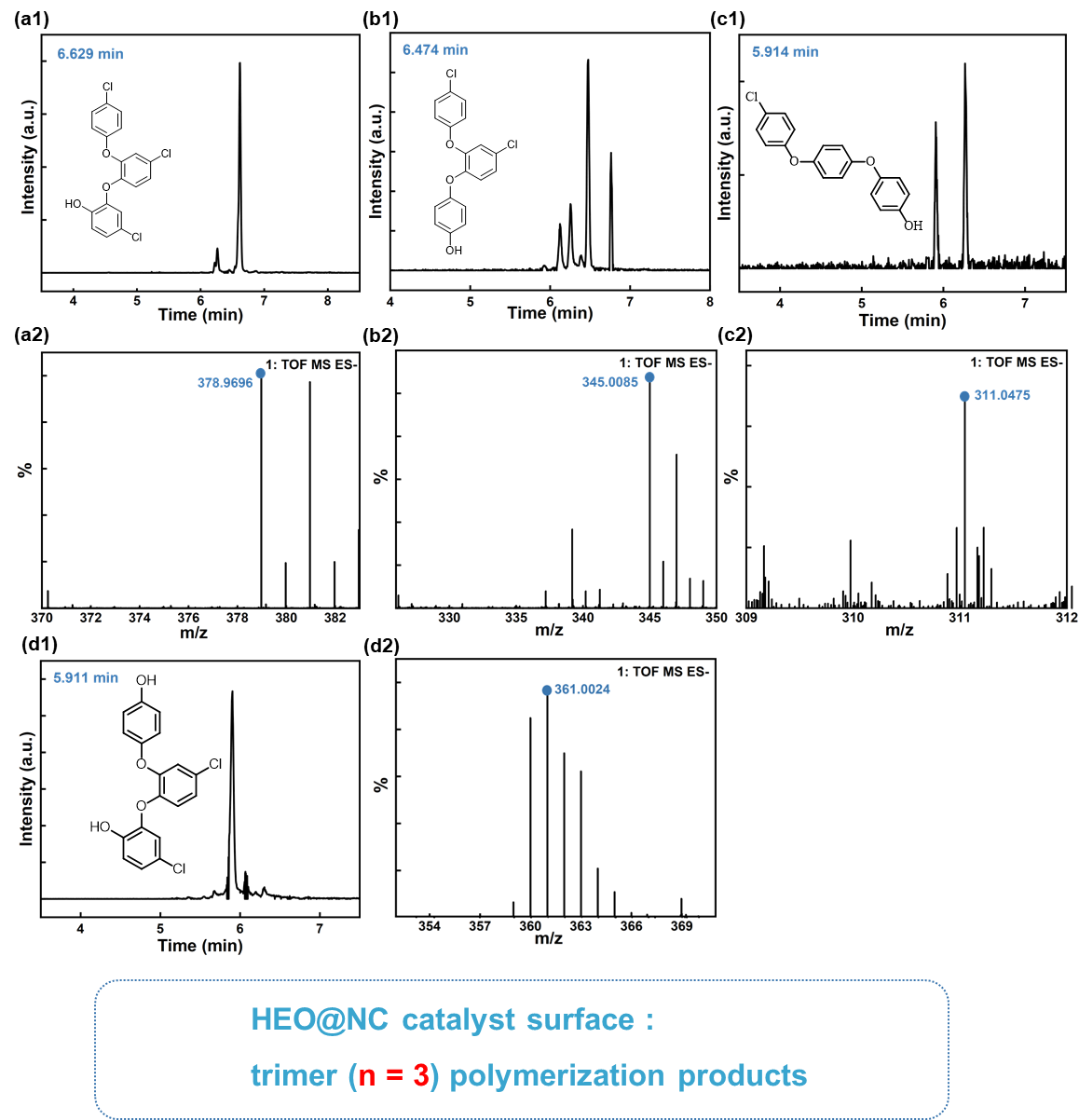


**Fig. S55 |** UPLC–QTOF-MS chromatograms of 4CP oxidation polymerization products in the HEO@NC catalyst surface, and their corresponding molecular ion mass spectra of the chromatographic peaks.


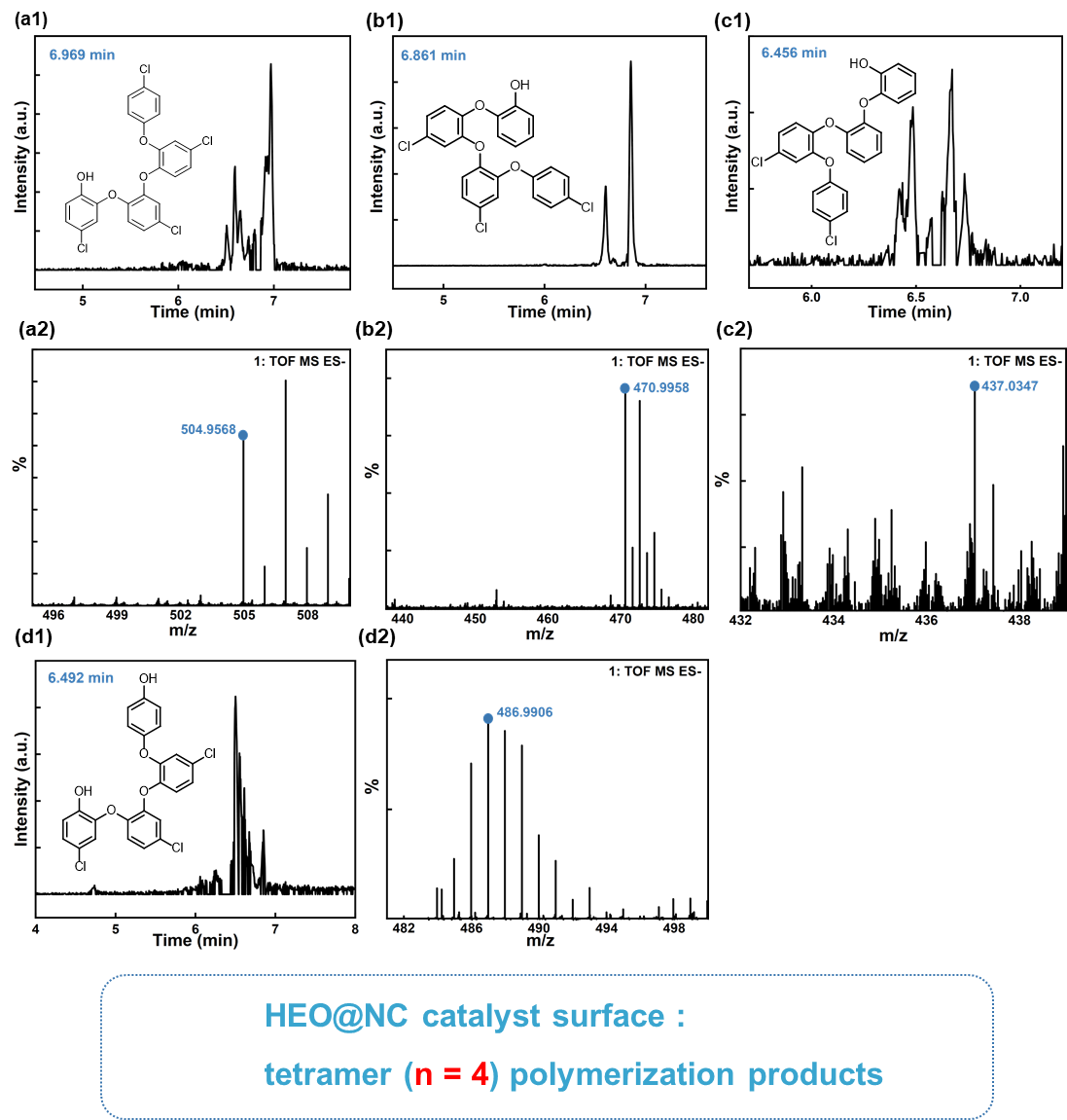


**Fig. S56 |** UPLC–QTOF-MS chromatograms of 4CP oxidation polymerization products in the HEO@NC catalyst surface, and their corresponding molecular ion mass spectra of the chromatographic peaks.


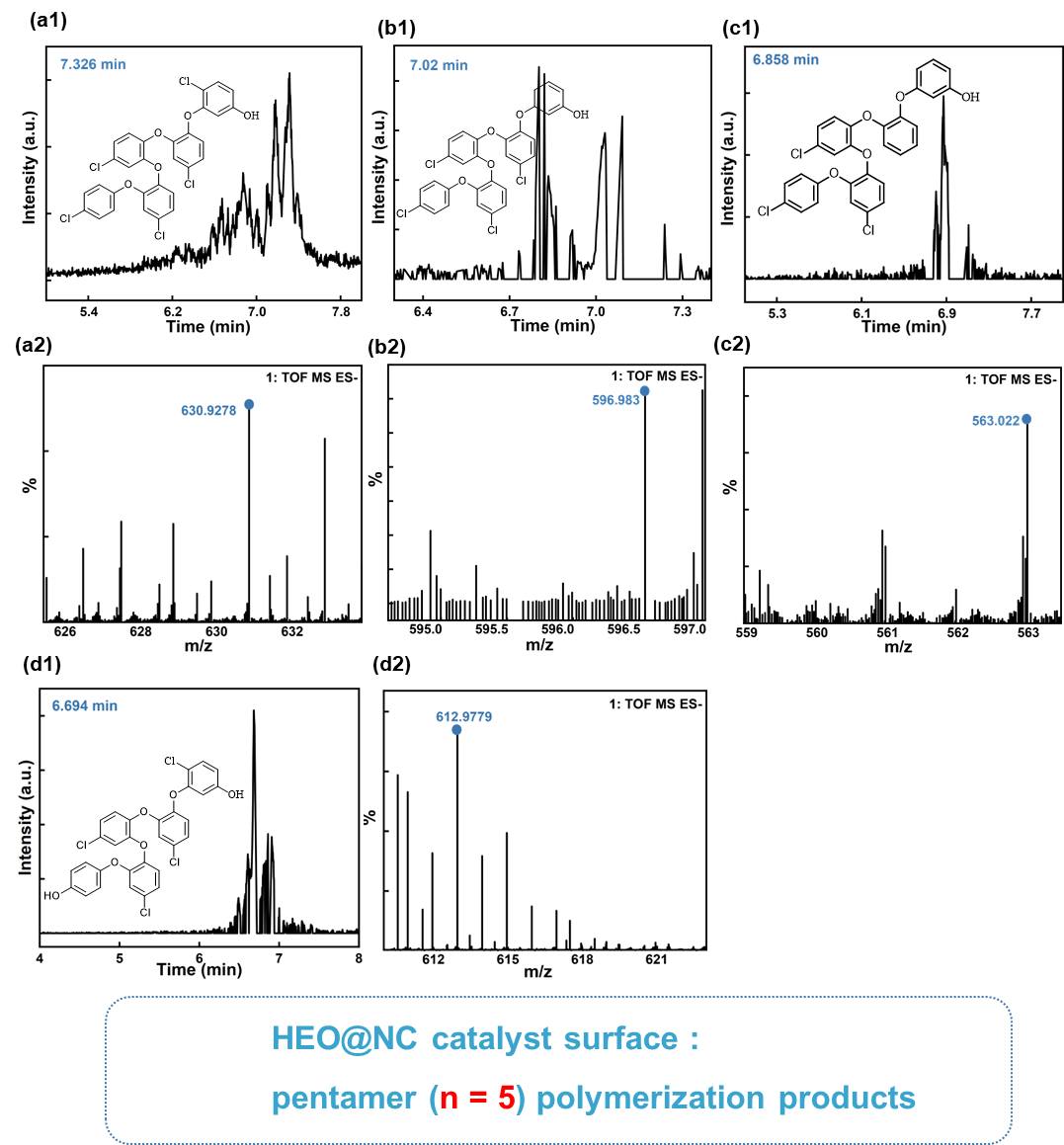


**Fig. S57 |** UPLC–QTOF-MS chromatograms of 4CP oxidation products in the HEO@NC catalyst surface, and their corresponding molecular ion mass spectra of the chromatographic peaks.


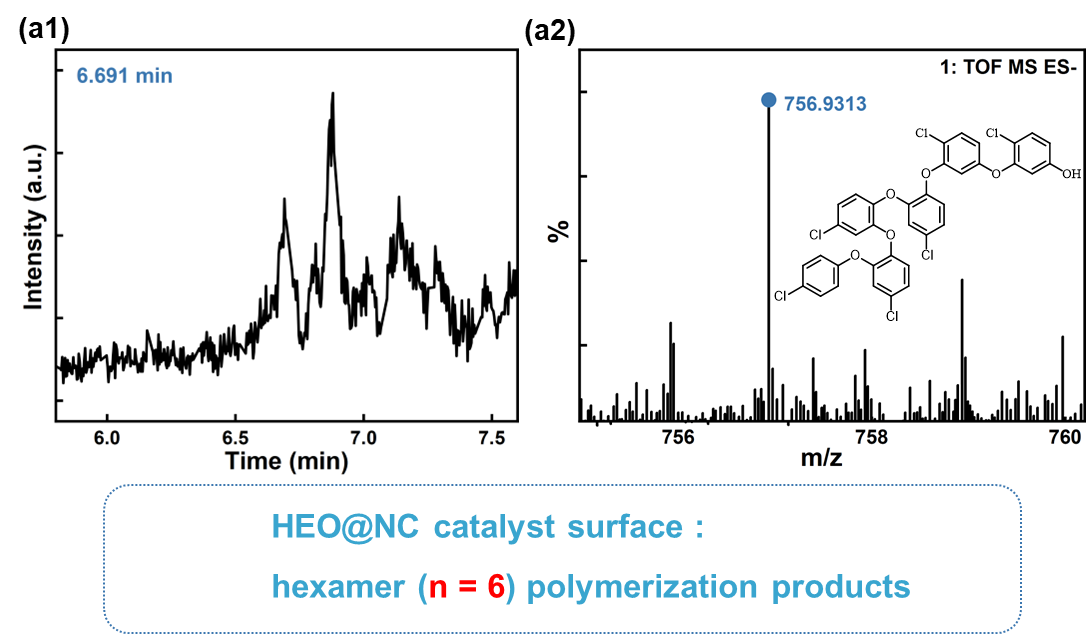


**Fig. S58 |** UPLC–QTOF-MS chromatograms of 4CP oxidation products in the HEO@NC catalyst surface, and their corresponding molecular ion mass spectra of the chromatographic peaks.


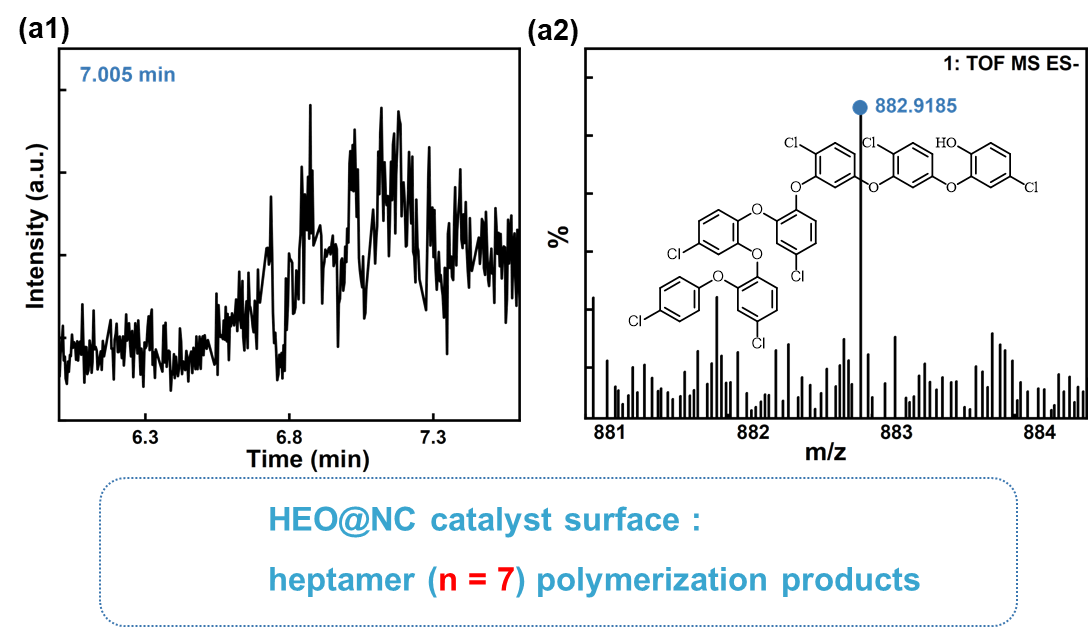


**Fig. S59 |** UPLC–QTOF-MS chromatograms of 4CP oxidation products in the HEO@NC catalyst surface, and their corresponding molecular ion mass spectra of the chromatographic peaks.


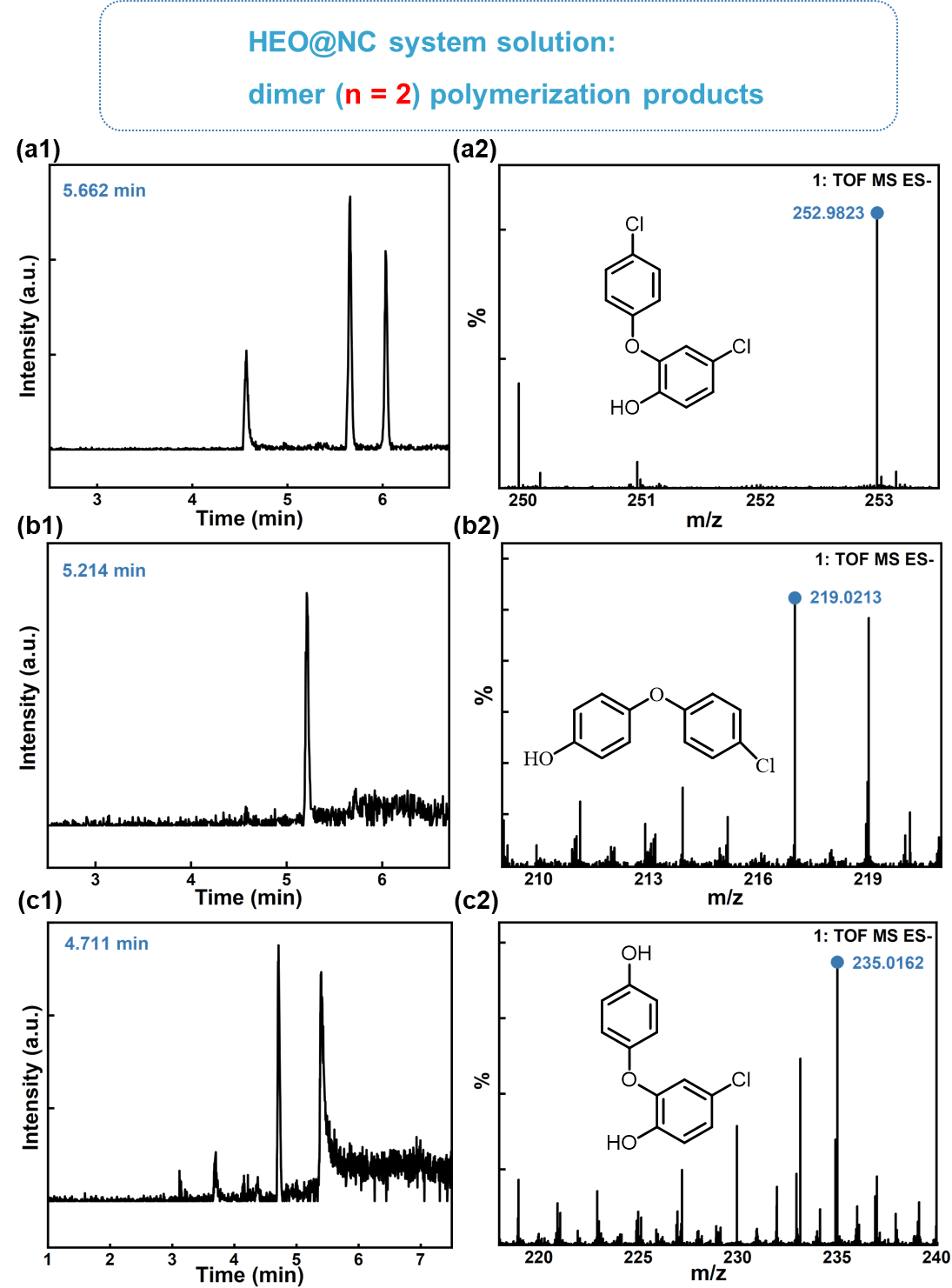


**Fig. S60 |** UPLC–QTOF-MS chromatograms of 4CP oxidation products in the HEO@NC-PI system solution, and their corresponding molecular ion mass spectra of the chromatographic peaks.


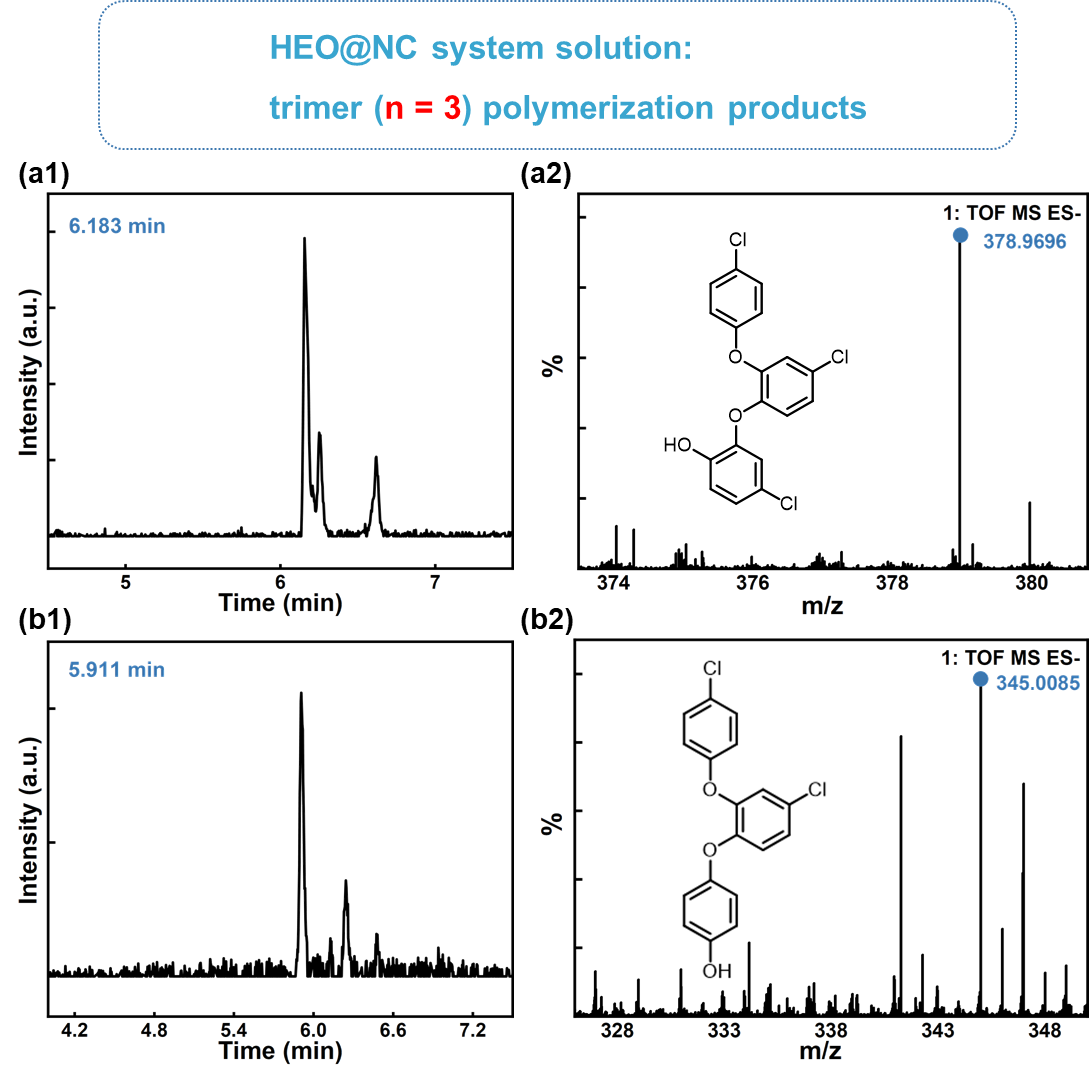


**Fig. S61 |** UPLC–QTOF-MS chromatograms of 4CP oxidation products in the HEO@NC-PI system solution, and their corresponding molecular ion mass spectra of the chromatographic peaks.


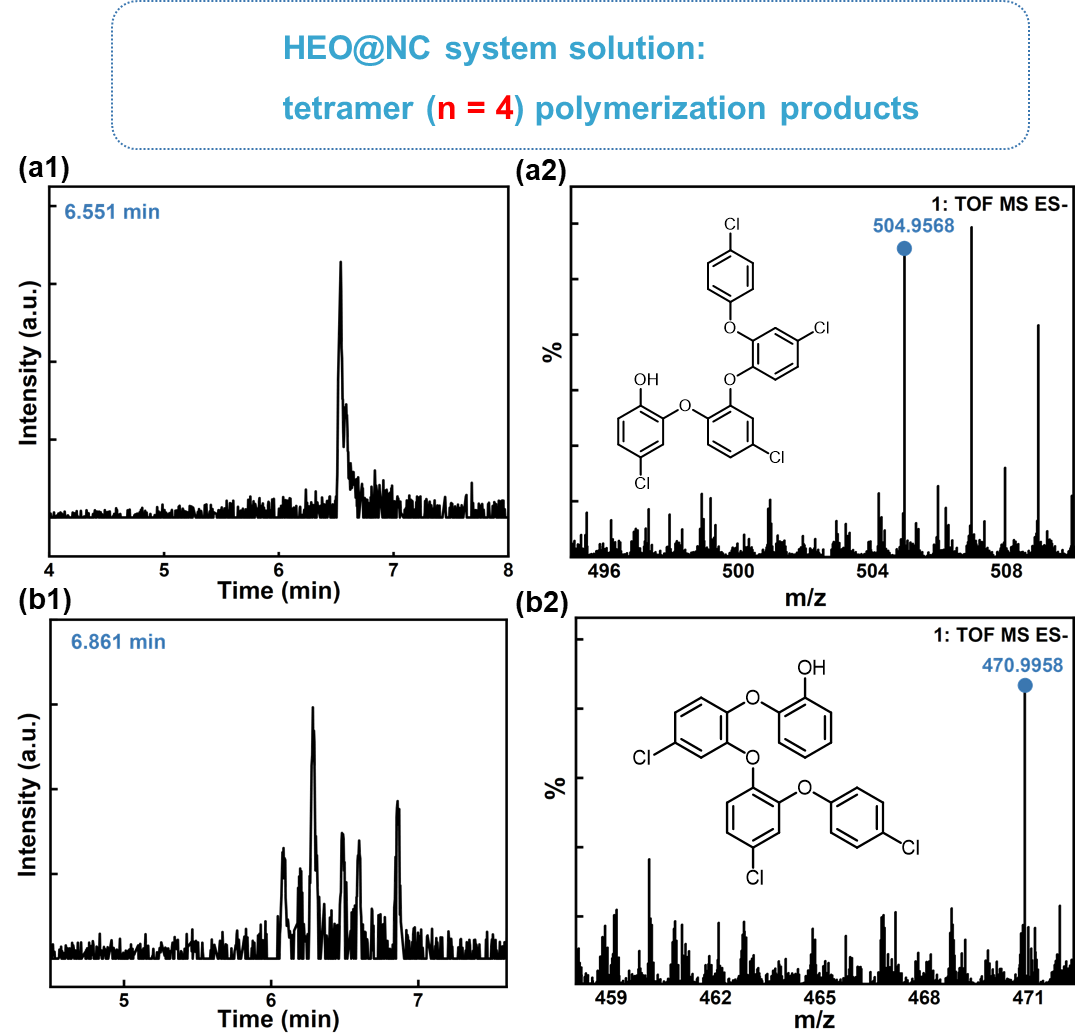


**Fig. S62 |** UPLC–QTOF-MS chromatograms of 4CP oxidation products in the HEO@NC-PI system solution, and their corresponding molecular ion mass spectra of the chromatographic peaks.


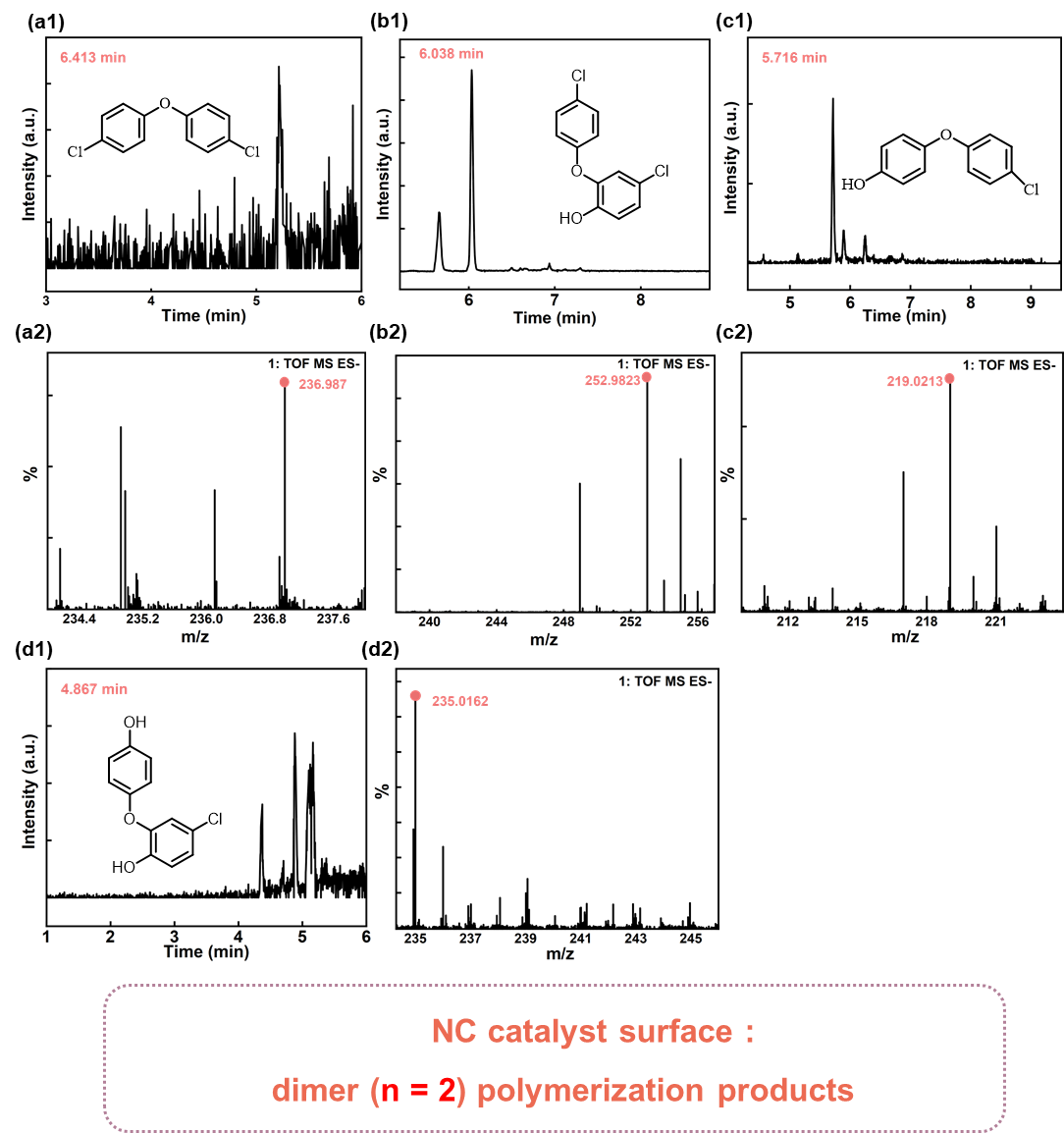


**Fig. S63 |** UPLC–QTOF-MS chromatograms of 4CP oxidation products in the NC catalyst surface, and their corresponding molecular ion mass spectra of the chromatographic peaks.


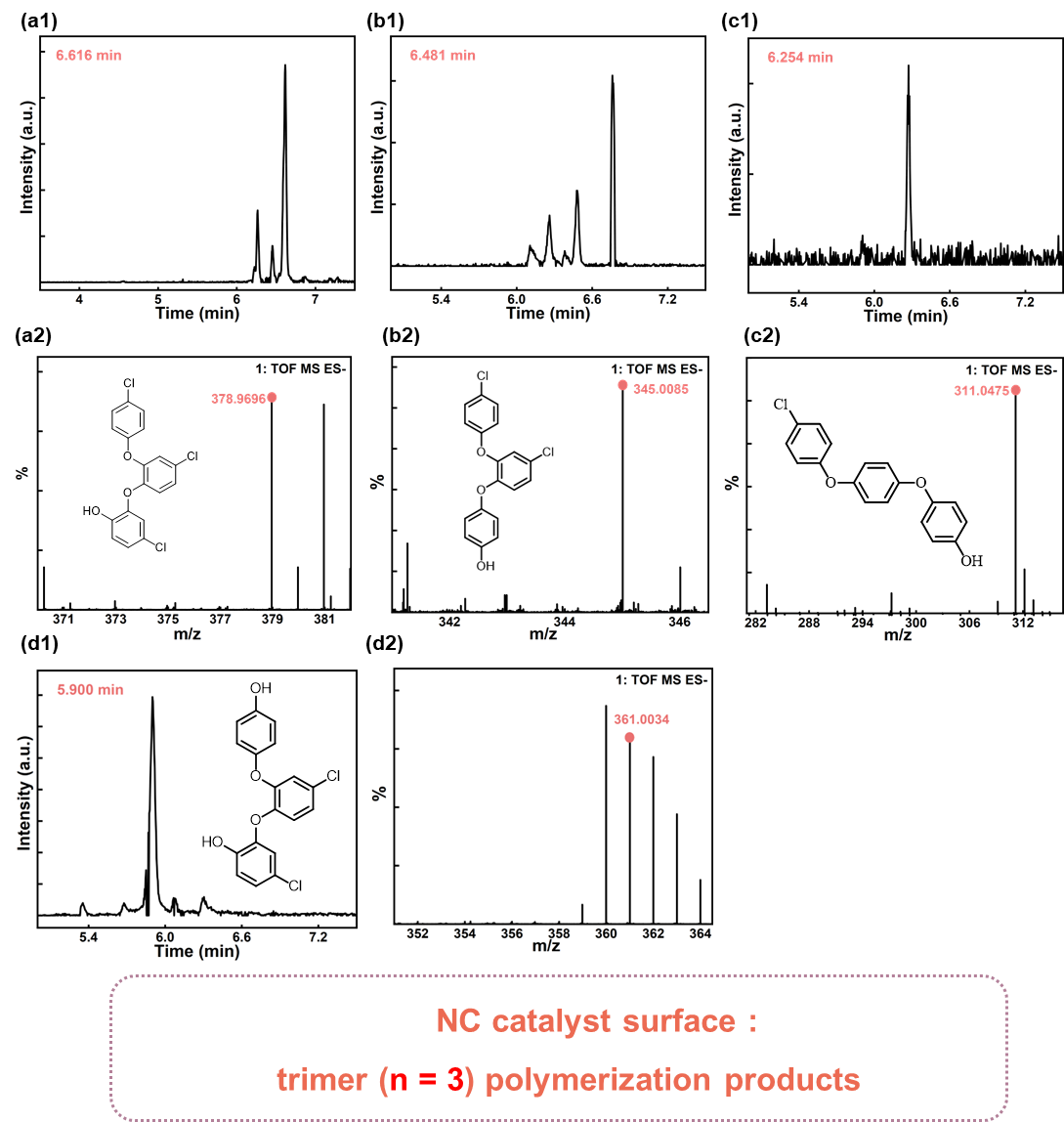


**Fig. S64 |** UPLC–QTOF-MS chromatograms of 4CP oxidation products in the NC catalyst surface, and their corresponding molecular ion mass spectra of the chromatographic peaks.


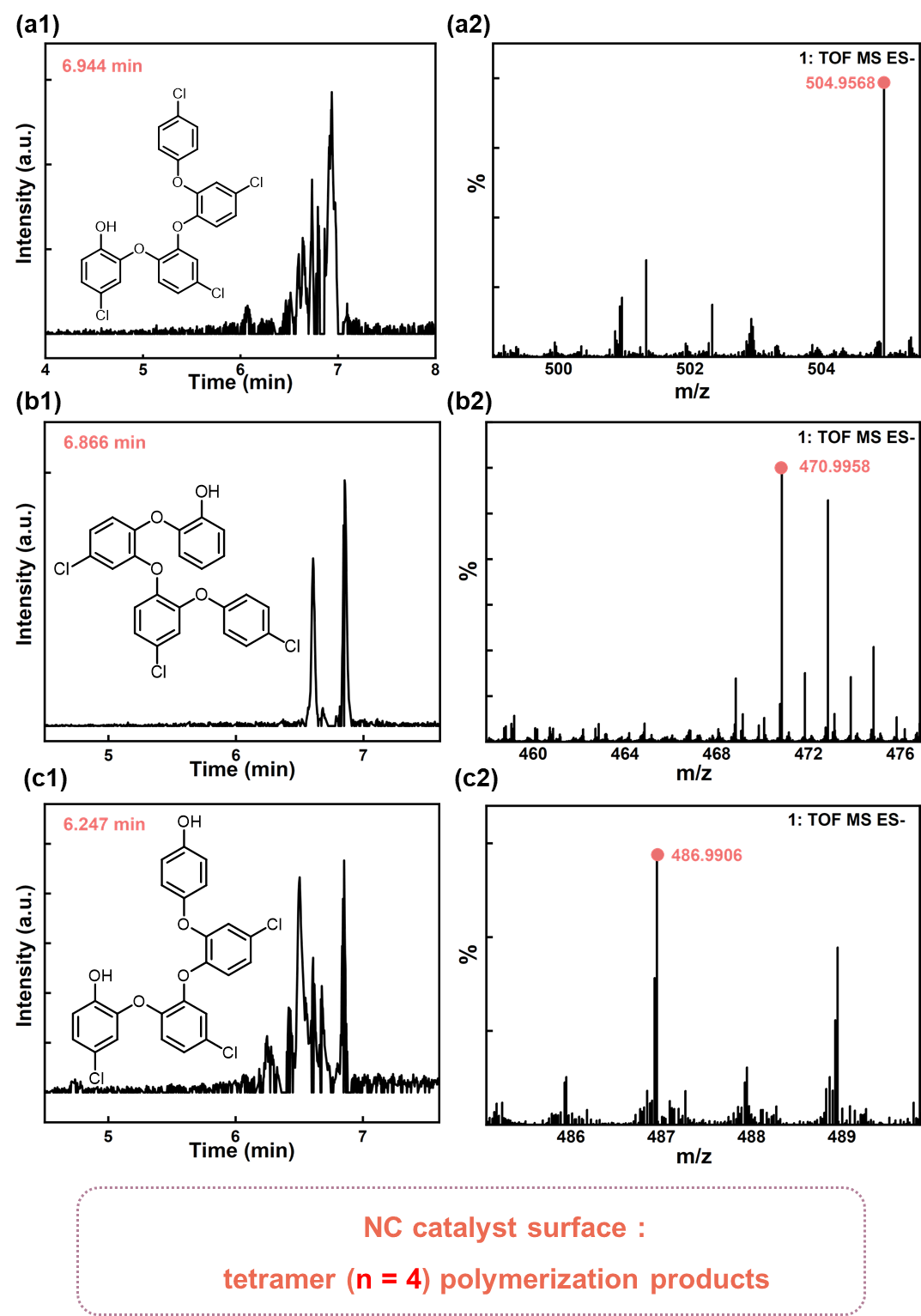


**Fig. S65 |** UPLC–QTOF-MS chromatograms of 4CP oxidation products in the NC catalyst surface, and their corresponding molecular ion mass spectra of the chromatographic peaks.


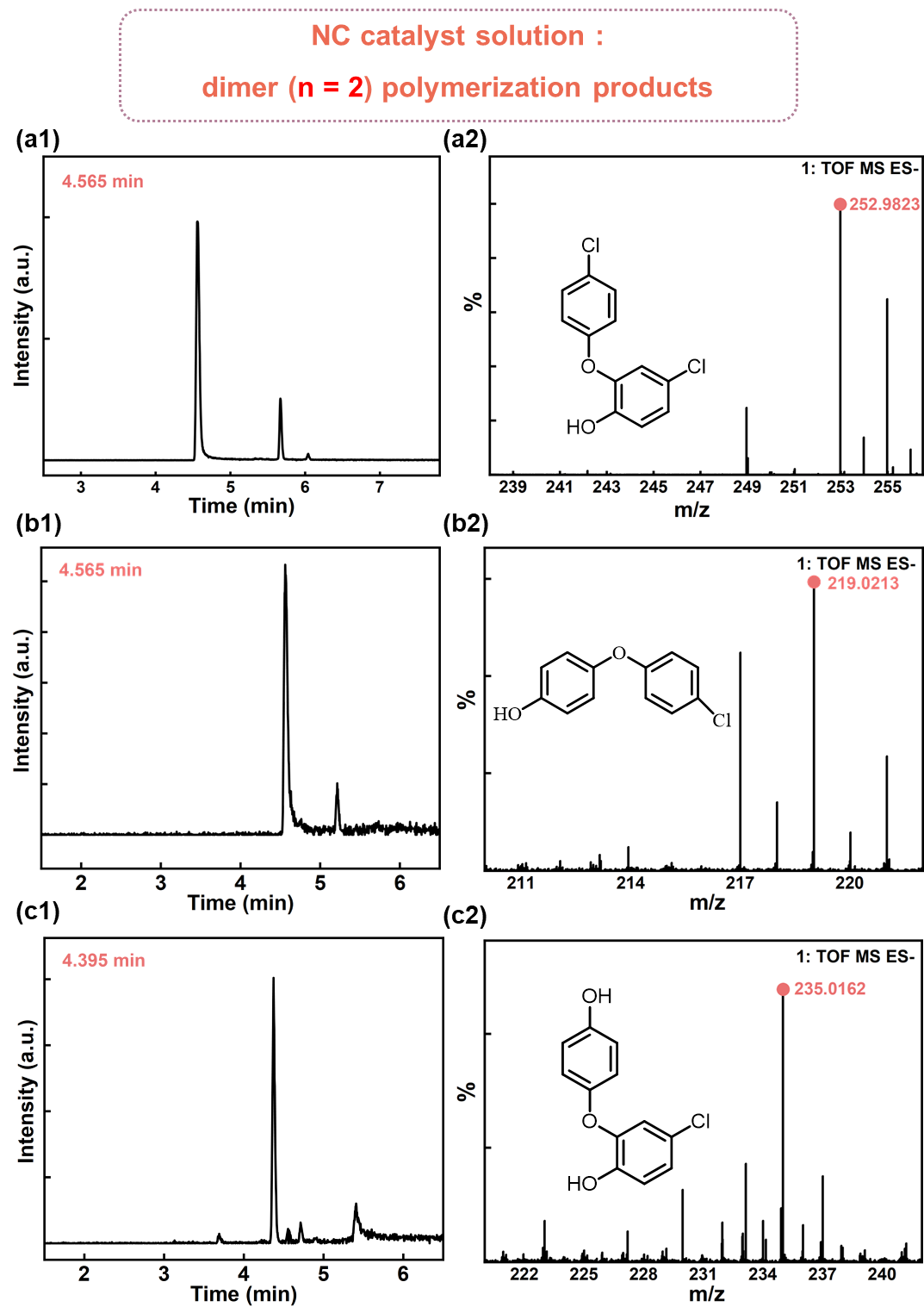


**Fig. S66 |** UPLC–QTOF-MS chromatograms of 4CP oxidation products in the NC-PI system solution, and their corresponding molecular ion mass spectra of the chromatographic peaks.


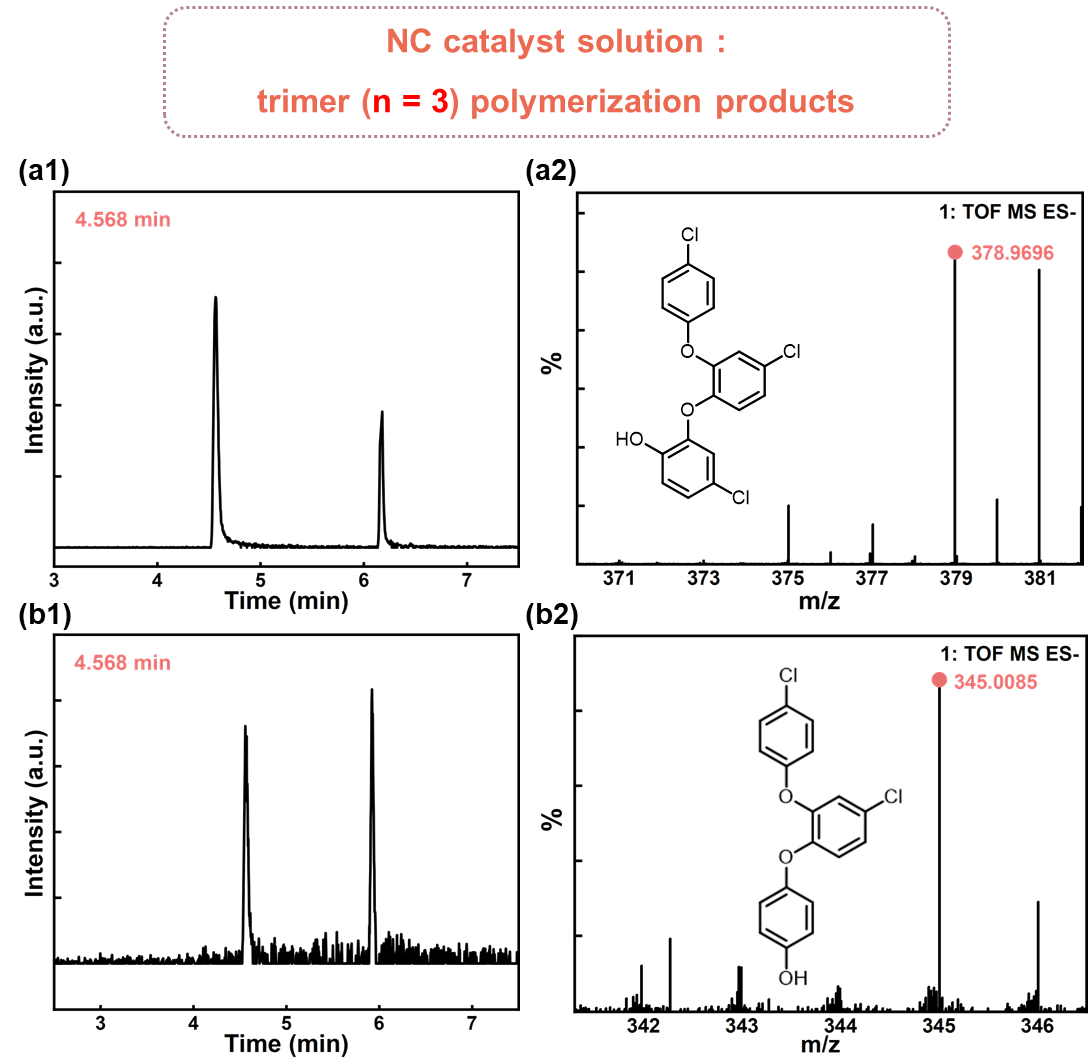


**Fig. S67 |** UPLC–QTOF-MS chromatograms of 4CP oxidation products in the NC-PI system solution, and their corresponding molecular ion mass spectra of the chromatographic peaks.


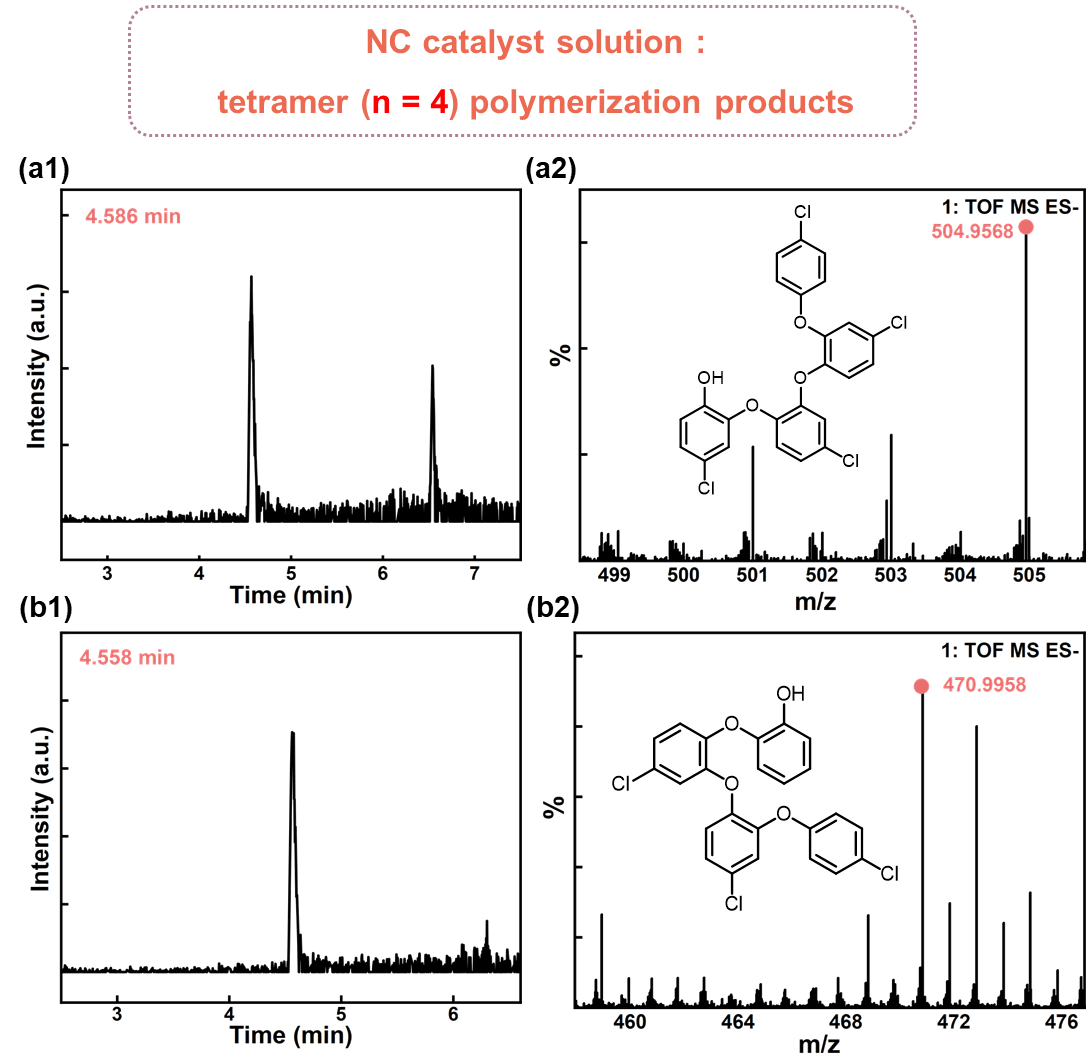


**Fig. S68 |** UPLC–QTOF-MS chromatograms of 4CP oxidation products in the NC-PI system solution, and their corresponding molecular ion mass spectra of the chromatographic peaks.


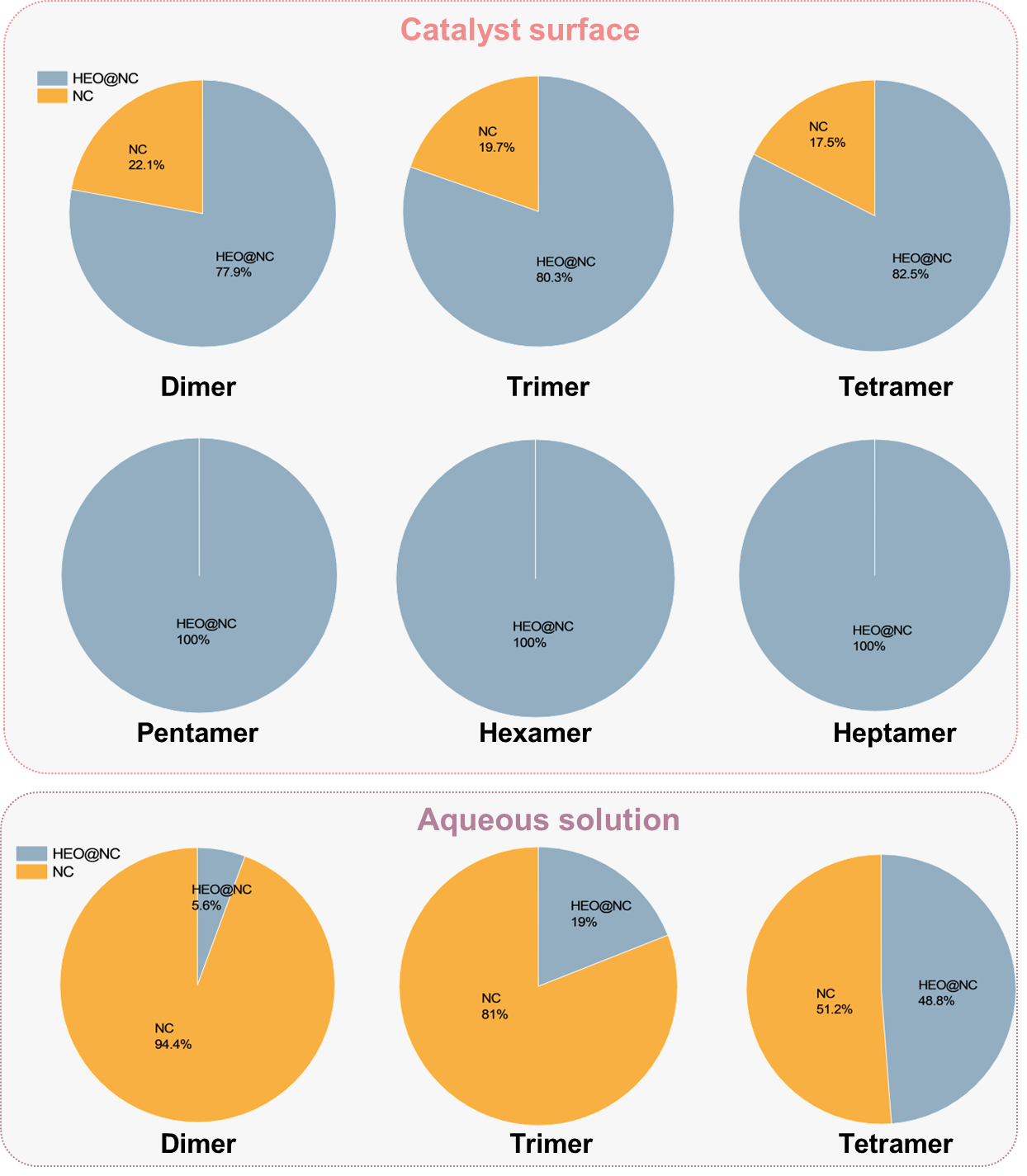


**Fig. S69 |** Comparison of polymeric product distributions across varying unit polymerization metrics in NC-PI and HEO@NC-PI systems.


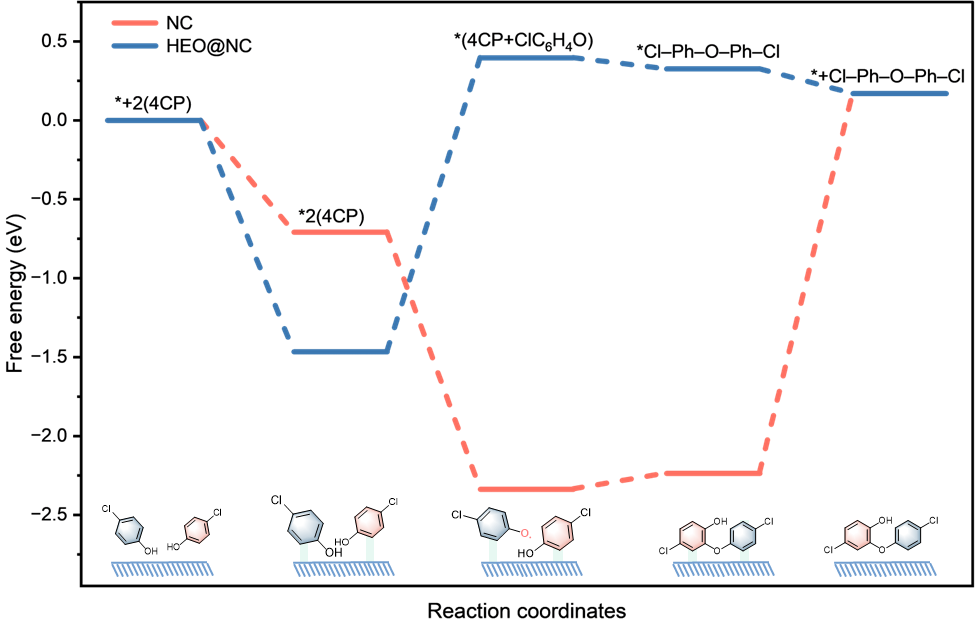


**Fig. S70 |** Thermodynamic potential energy curves during the polymerization reaction.


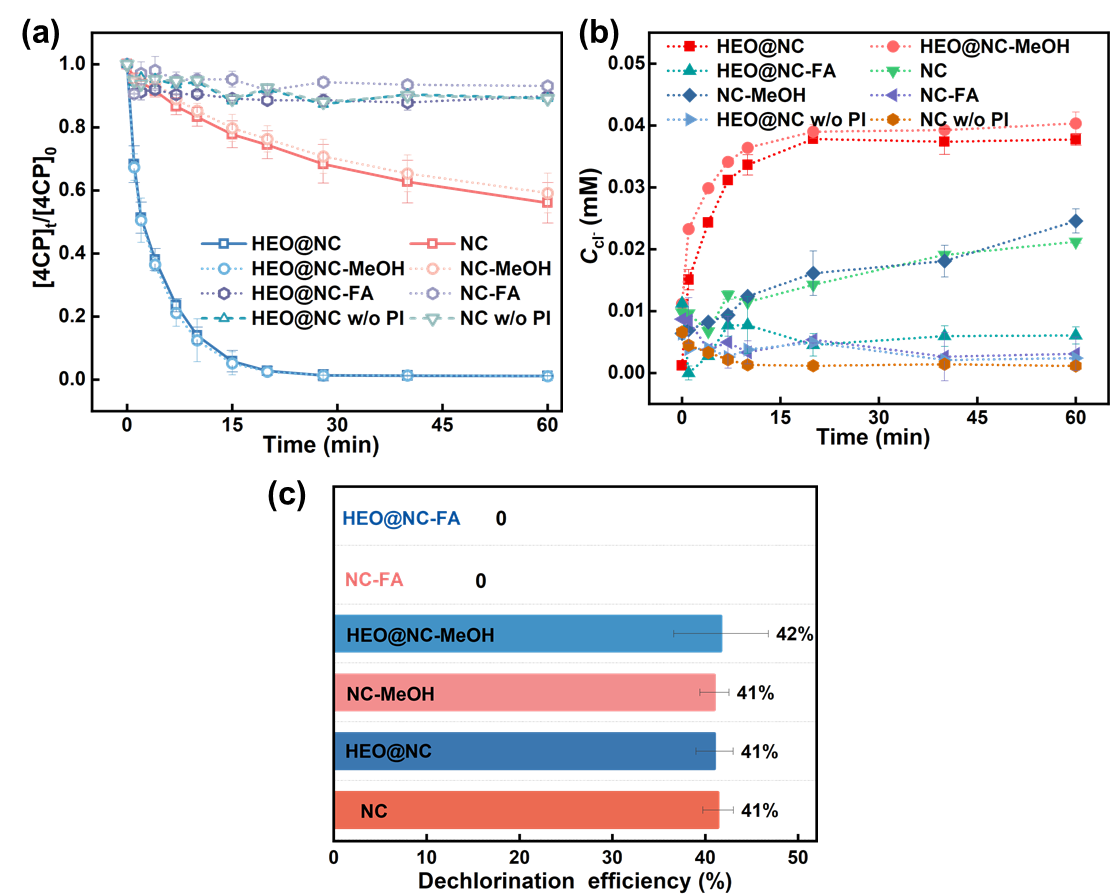


**Fig. S71 |** The effect of various scavengers on 4CP removal (a) and dechlorination concentration (b) by NC-PI and HEO@NC-PI systems. (c) Comparison of dechlorination levels in different systems.

The use of methanol as a quenching agent does not affect the kinetic rate or thermodynamic endpoint of dechlorination, indicating that atomic hydrogen (H^*^) is not the primary active species responsible for dechlorination. This observation confirms the absence of reductive dechlorination pathways in both NC-PI and HEO@NC-PI systems. Furthermore, the lack of oxidative dechlorination mediated by freely diffusing hydroxyl radicals (^•^OH) in the bulk solution is evidenced by the complete termination of dechlorination upon addition of FA. These results collectively suggest that polymerization reactions play a critical role in the dechlorination process, while the hydroxylated dechlorination products likely originate from ^•^OH_ads_ localized on the catalyst surface.


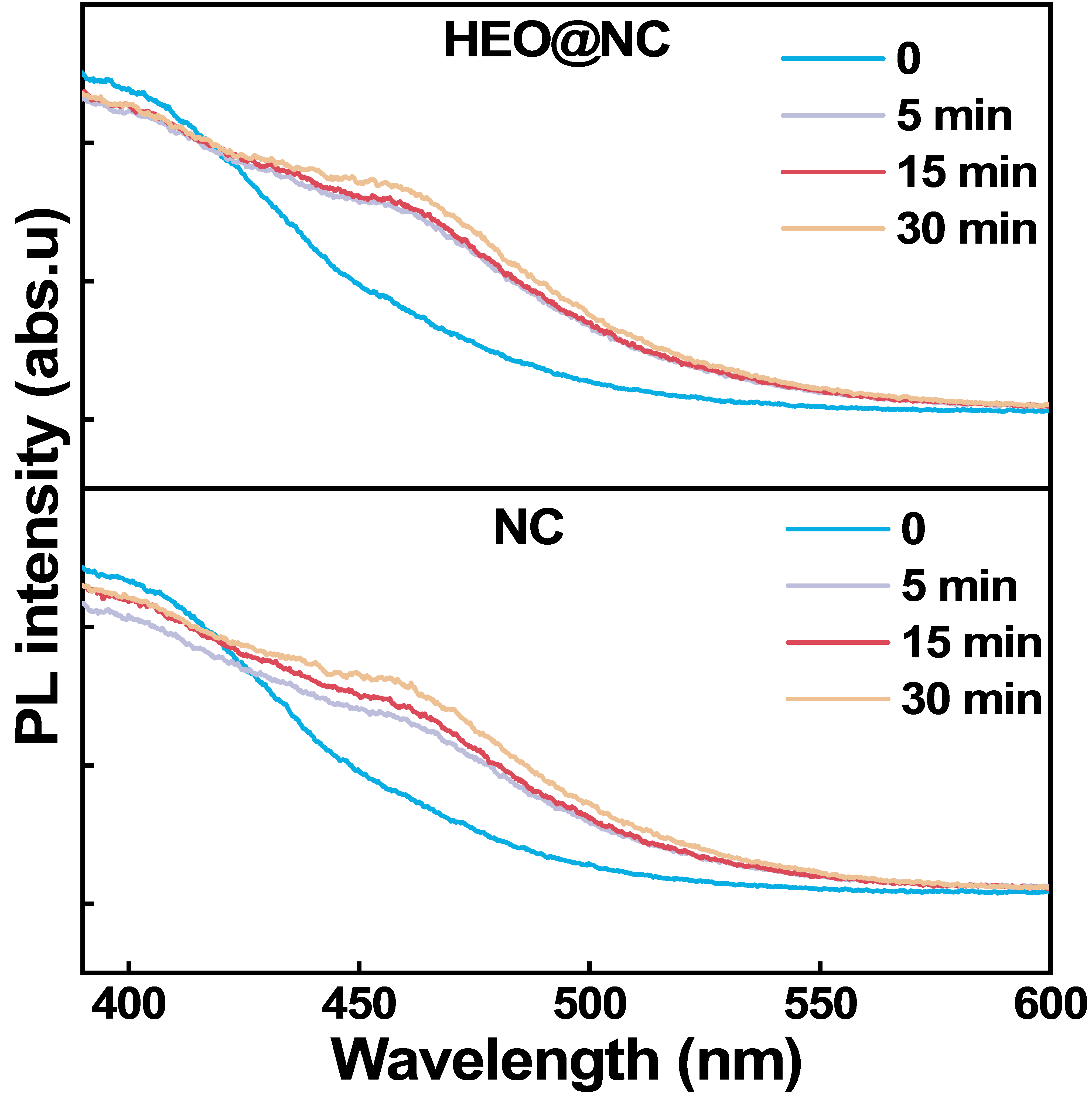


**Fig. S72 |** Fluorescence detection of adsorbed hydroxyl radicals in coumarin reaction systems using NC and HEO@NC.

In both the NC-PI and HEO@NC-PI systems, distinct 7-hydroxycoumarin products were detected at 460 nm, though the weak peak signals suggest the presence of only a small amount of surface-adsorbed hydroxyl radicals (^•^OH_ads_) in the system. This observation aligns with the methanol quenching experiment, which showed no significant impact on 4CP removal.


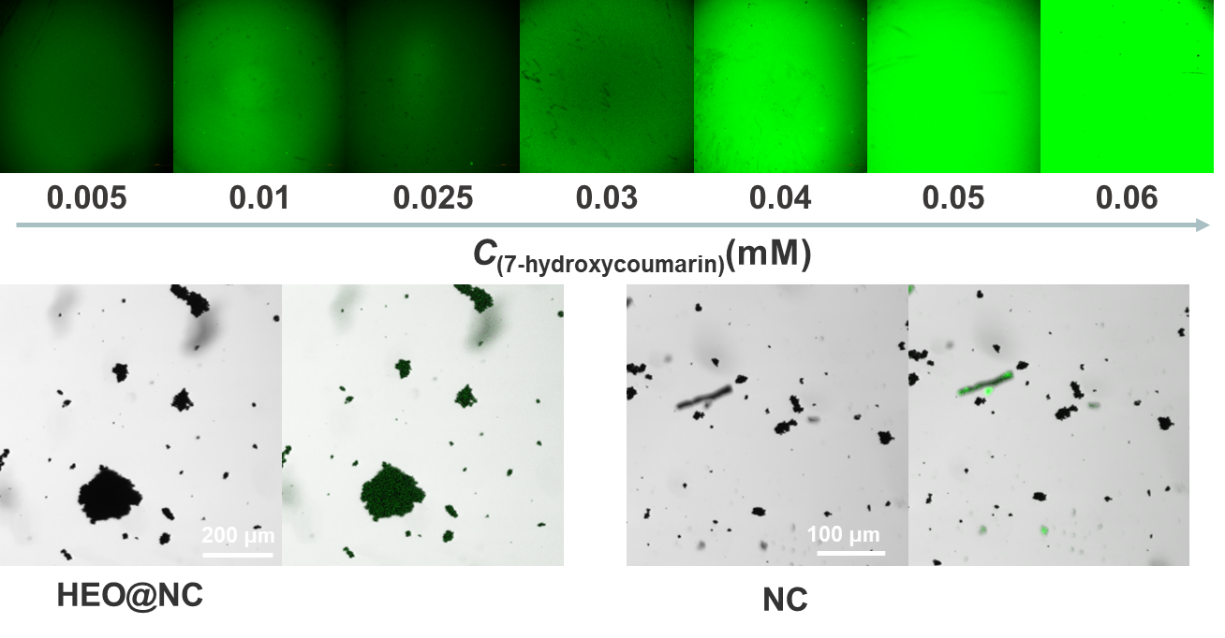


**Fig. S73 |** Colorimetric references of 7-hydroxycoumarin solutions and fluorescence microscopy images of the NC-PI and HEO@NC-PI systems.

Fluorescence microscopy shows strong fluorescence during the reaction, directly confirming the presence of ^•^OH_ads_ at the catalyst interface and its involvement in dechlorination.


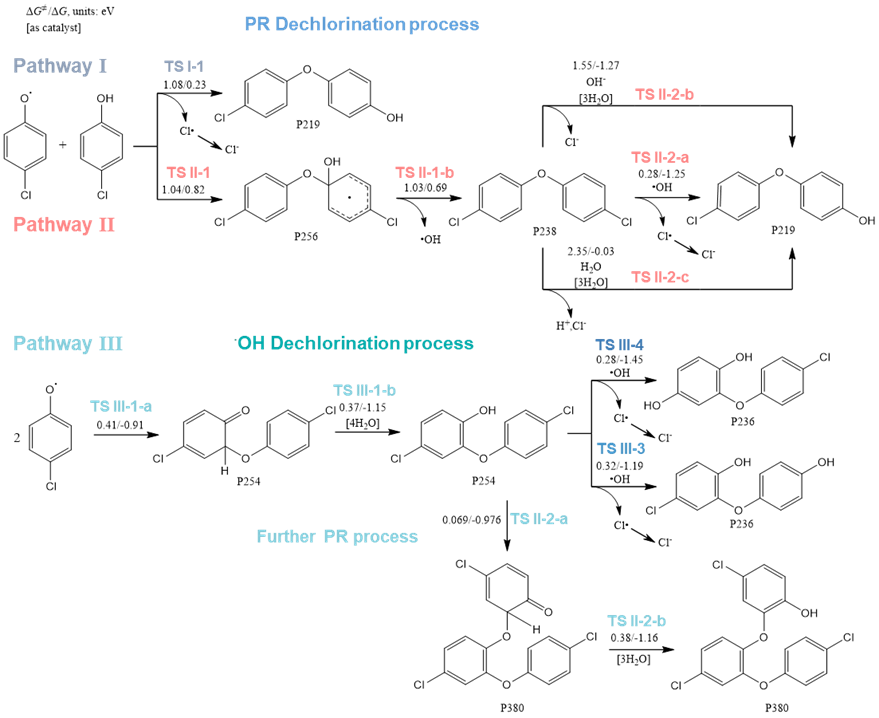


**Fig. S74 |** Proposed reaction pathways for PR and **^·^**OH-mediated dechlorination in NC-PI and HEO@NC-PI systems

**
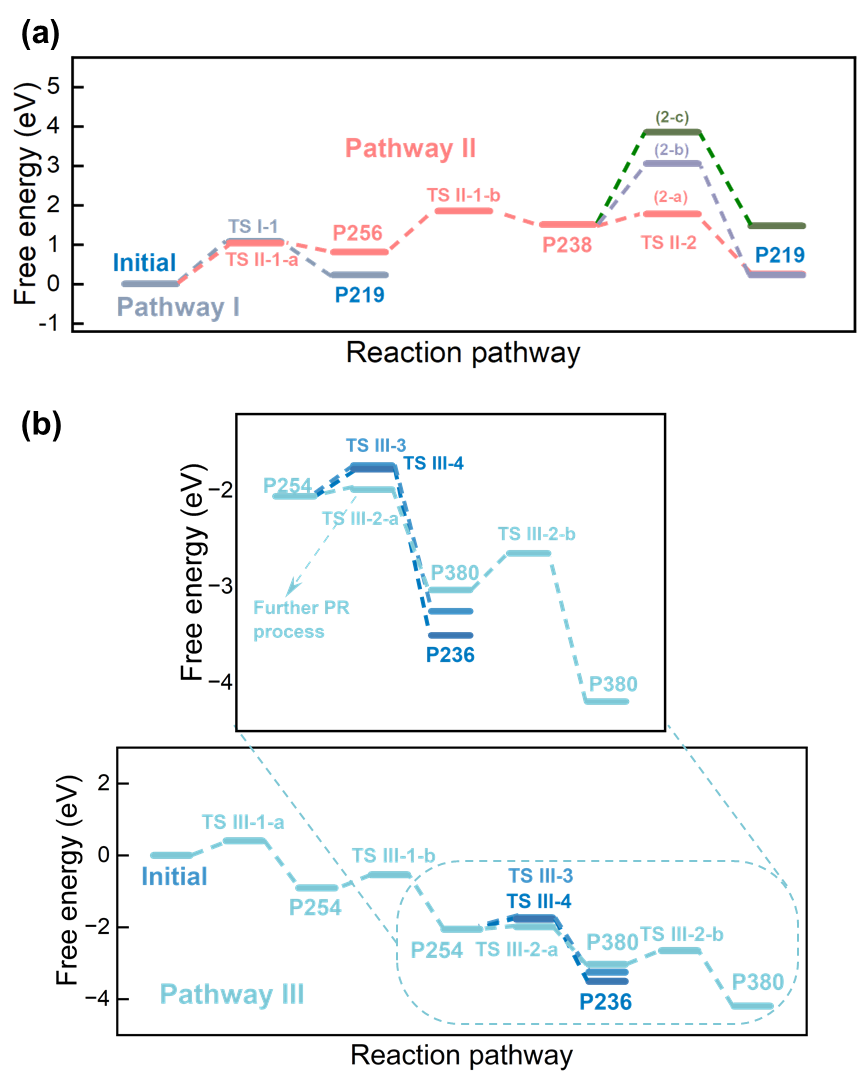
**

**Fig. S75 |** Reaction energy barriers of the different PR and **^·^**OH-mediated dechlorination pathways.


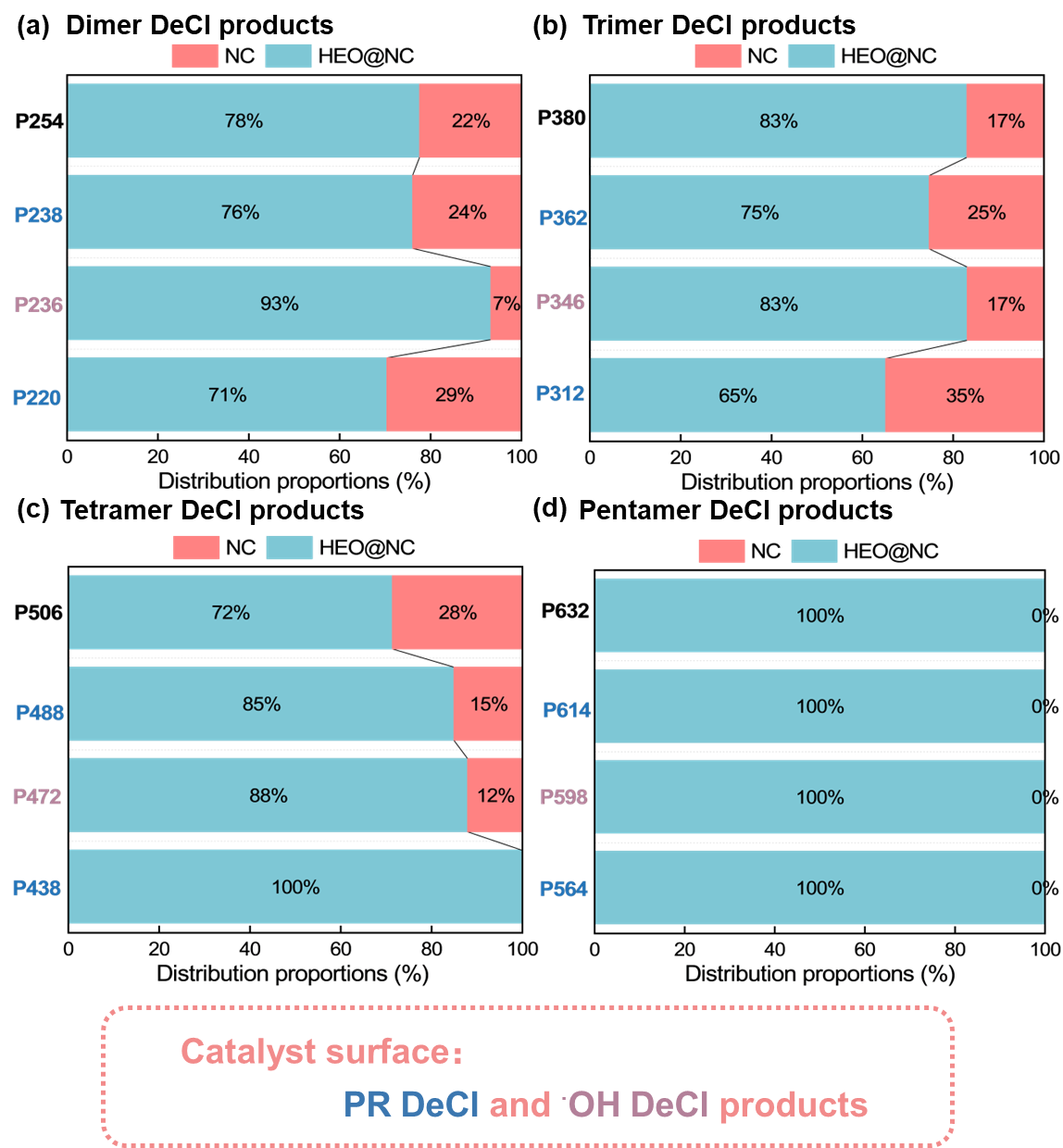


**Fig. S76 |** Comparison of different polymeric product distributions on the NC and HEO@NC catalyst surface. **Blue** and **purple** color font indicate PR and **^·^**OH-mediated dechlorination products, respectively.


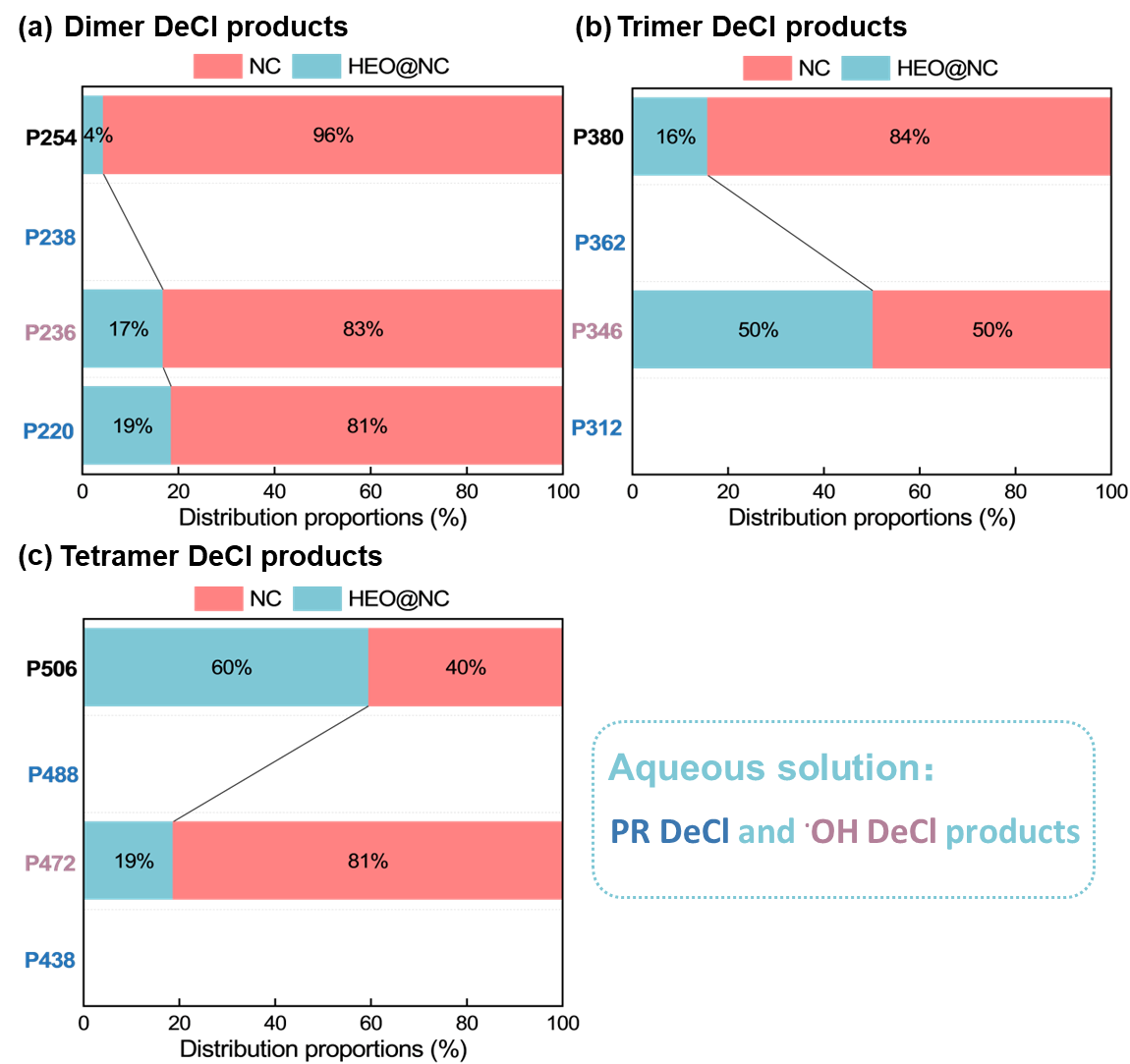


**Fig. S77 |** Comparison of different polymeric product distributions in the NC-PI and HEO@NC-PI system solution. **Blue** and **purple** color fonts indicate PR and **^·^**OH-mediated dechlorination products, respectively.


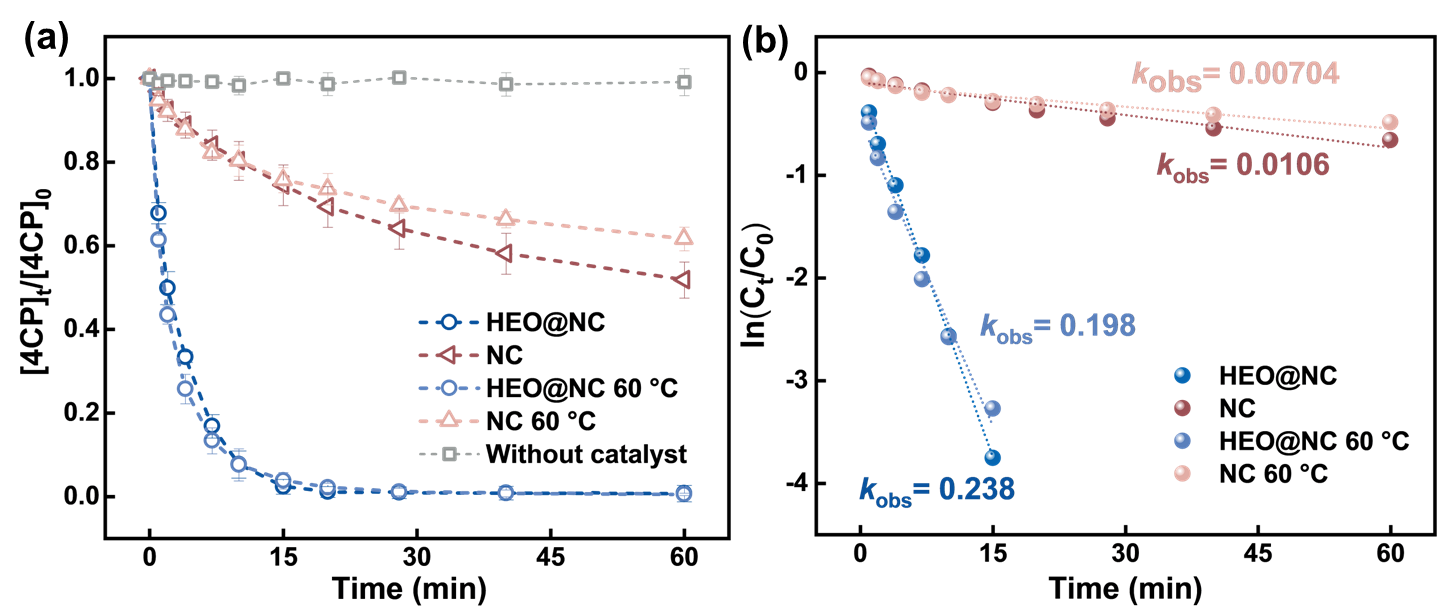


**Fig. S78 |** Catalytic performance (a) and apparent rate constants (b) for 4-CP removal at 60 °C. Dosage: [4CP]_0_: 0.1 mM, PI: 0.5 mM, reaction solution: 50 mL, catalyst: 0.1 g/L, reaction time: 60 min.

To elucidate how temperature influences polymer chain growth and product spatial distribution, UPLC–QTOF–MS was employed to separately analyze surface-confined and solution-phase products under elevated temperature conditions. Notably, when the temperature was increased to 60 °C, the oxidation kinetics of 4CP decreased rather than accelerated. This behavior indicates that the electron-transfer-mediated oxidative polymerization process, followed by surface enrichment of products; these processes are overall exothermic. Unlike conventional oxidants such as PMS or PDS, for which temperature mainly regulates the generation of reactive oxygen species, temperature alone is insufficient to activate PI. Although increasing the temperature exerts only a minor influence on the overall reaction rate, it significantly affects both the degree of polymerization and the surface enrichment behavior of the products.


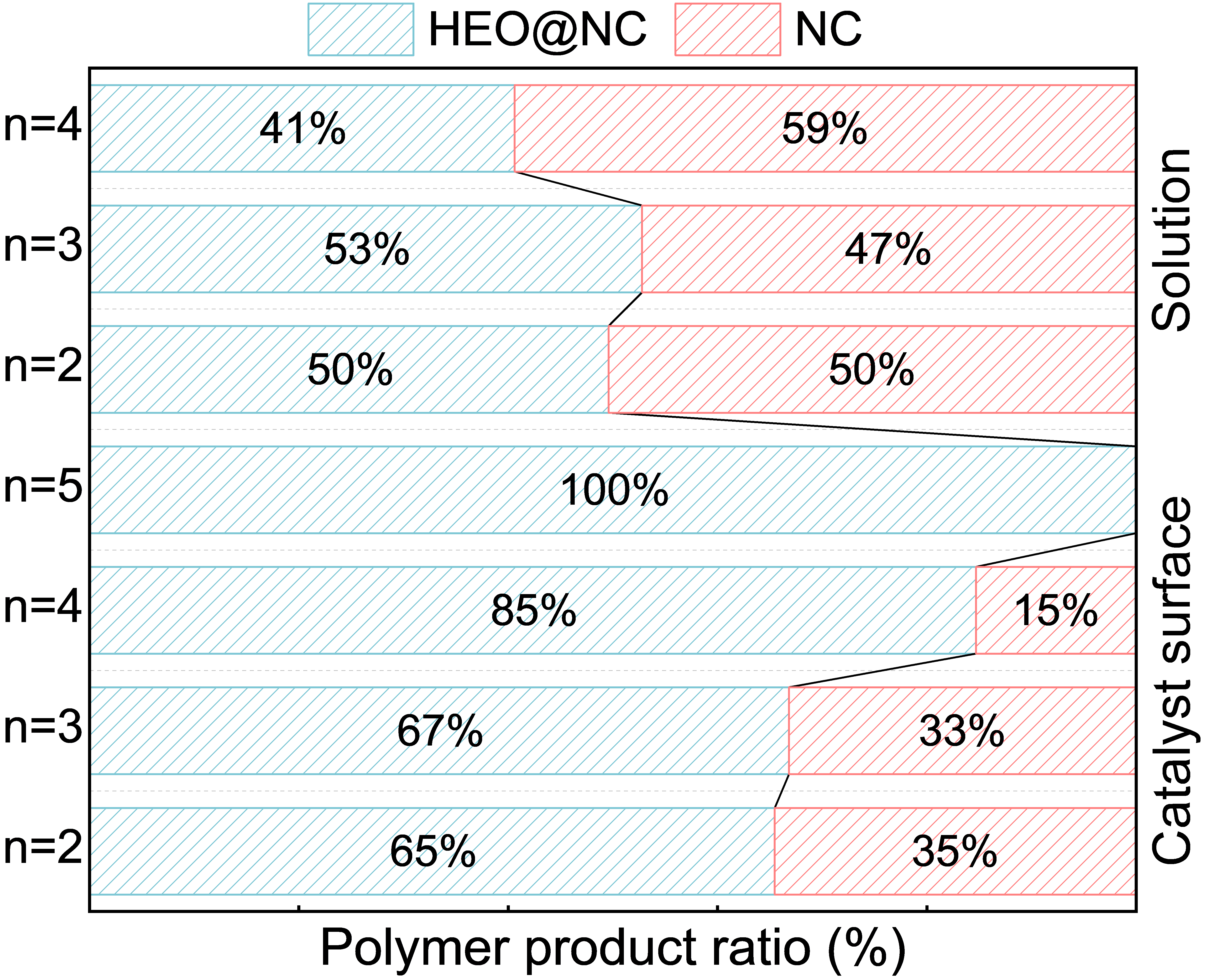


**Fig. S79 |** Comparison of polymeric product distributions by polymerization degree in the NC-PI and HEO@NC-PI systems at 60 °C.

Specifically, higher temperatures suppress the formation of highly polymerized species and significantly reduce their accumulation on the catalyst surface. Consistent with this observation, the concentration of polymeric products detected in the bulk solution increases with temperature in both HEO@NC–PI and NC–PI systems.


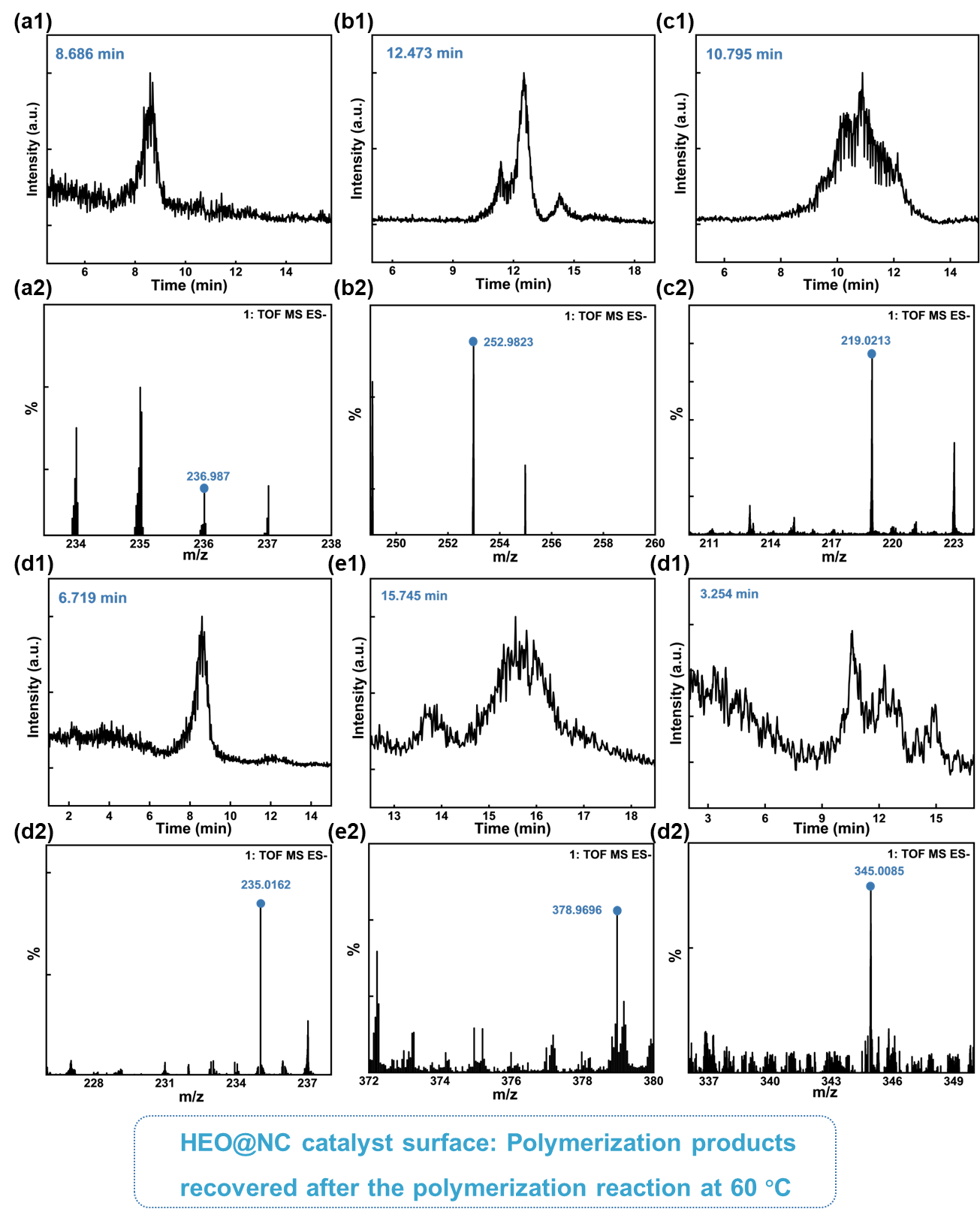


**Fig. S80 |** UPLC-QTOF-MS chromatograms of the polymerization products collected from the 4CP oxidation reaction at 60 °C on the surface of the HEO@NC catalyst, and the corresponding molecular ion mass spectra of the chromatographic peaks.


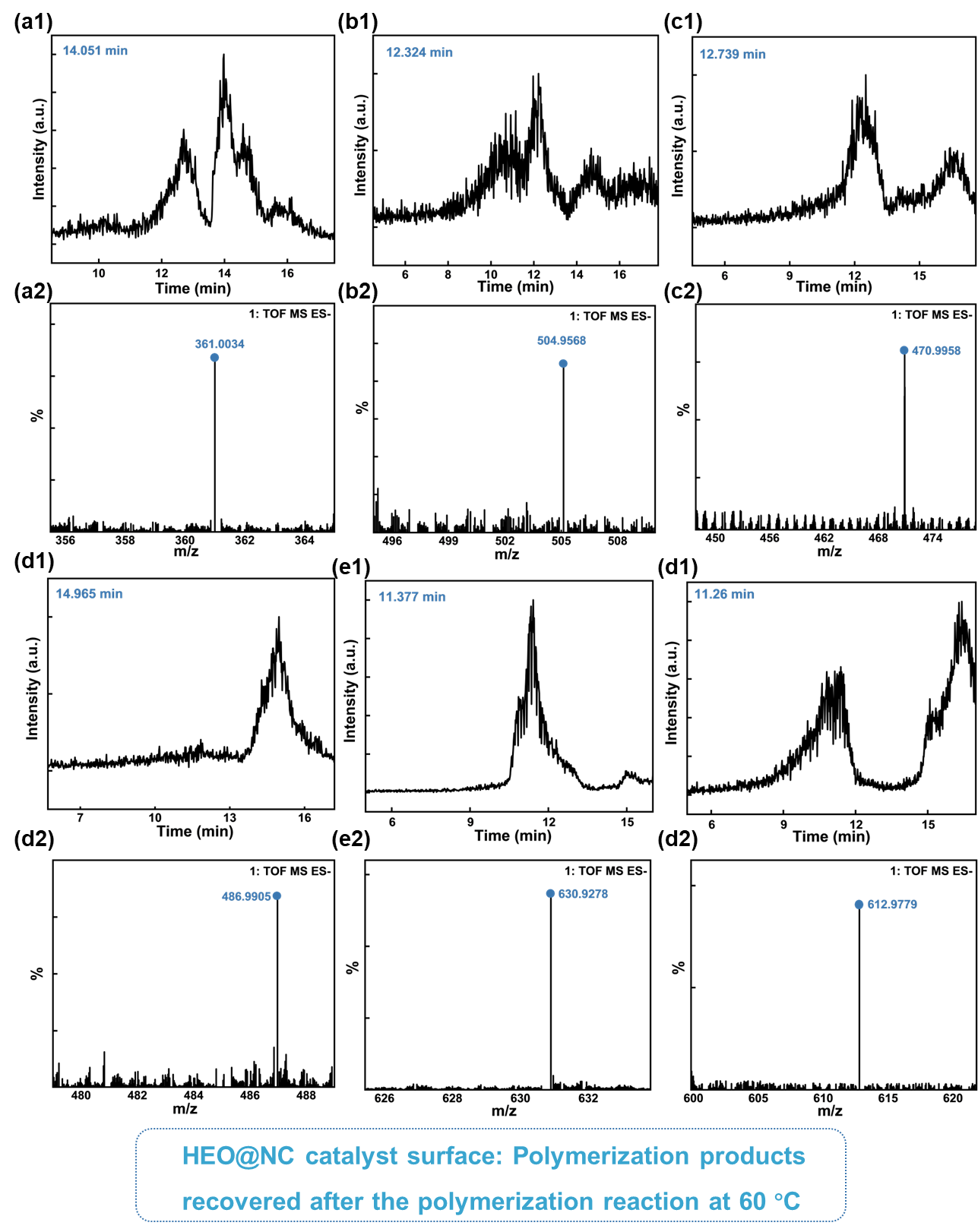


**Fig. S81 |** UPLC-QTOF-MS chromatograms of the polymerization products collected from the 4CP oxidation reaction at 60 °C on the surface of the HEO@NC catalyst, and the corresponding molecular ion mass spectra of the chromatographic peaks.


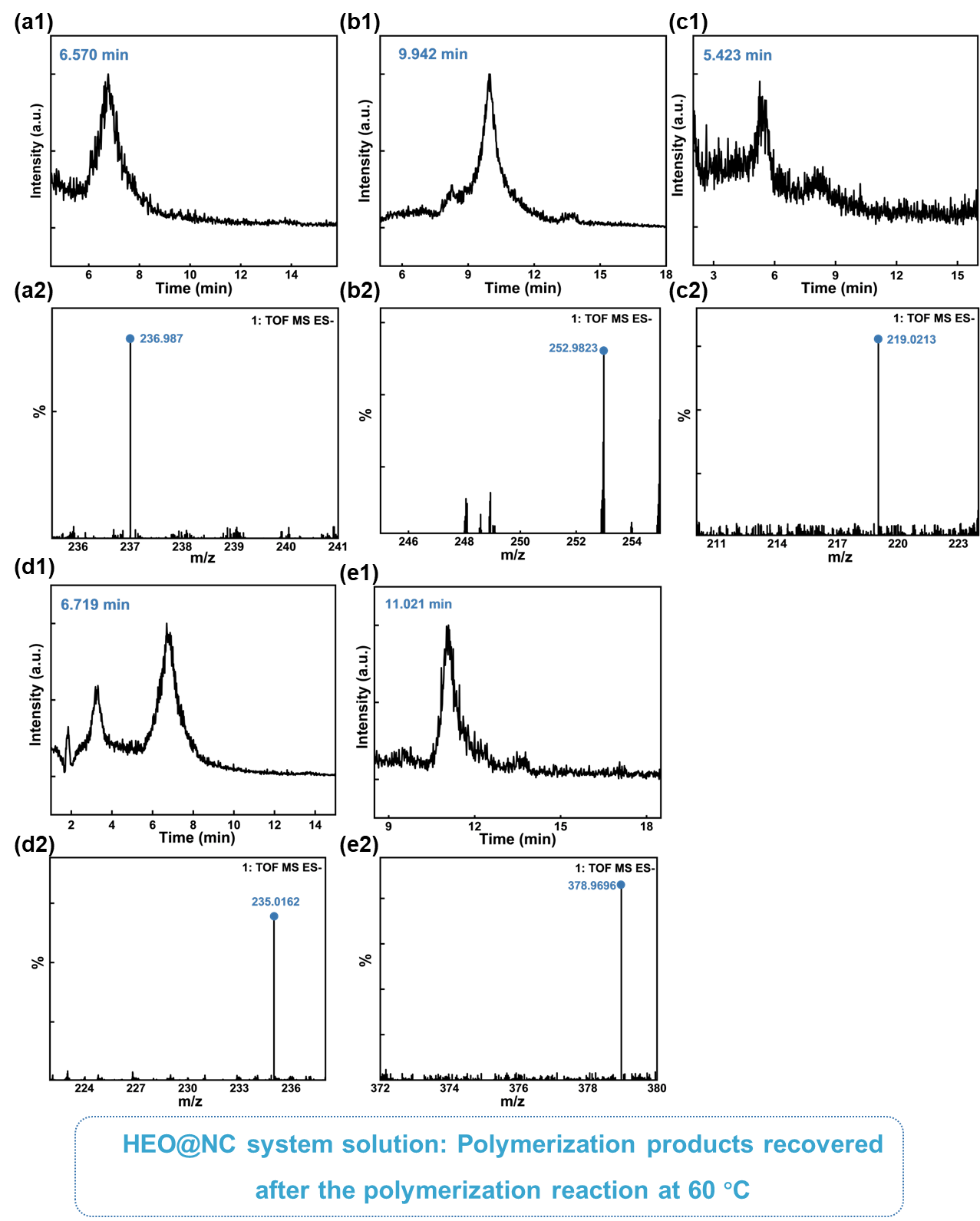


**Fig. S82 |** UPLC-QTOF-MS chromatograms of the polymerization products collected from the 4CP oxidation reaction at 60 °C on the HEO@NC system solution, and the corresponding molecular ion mass spectra of the chromatographic peaks.


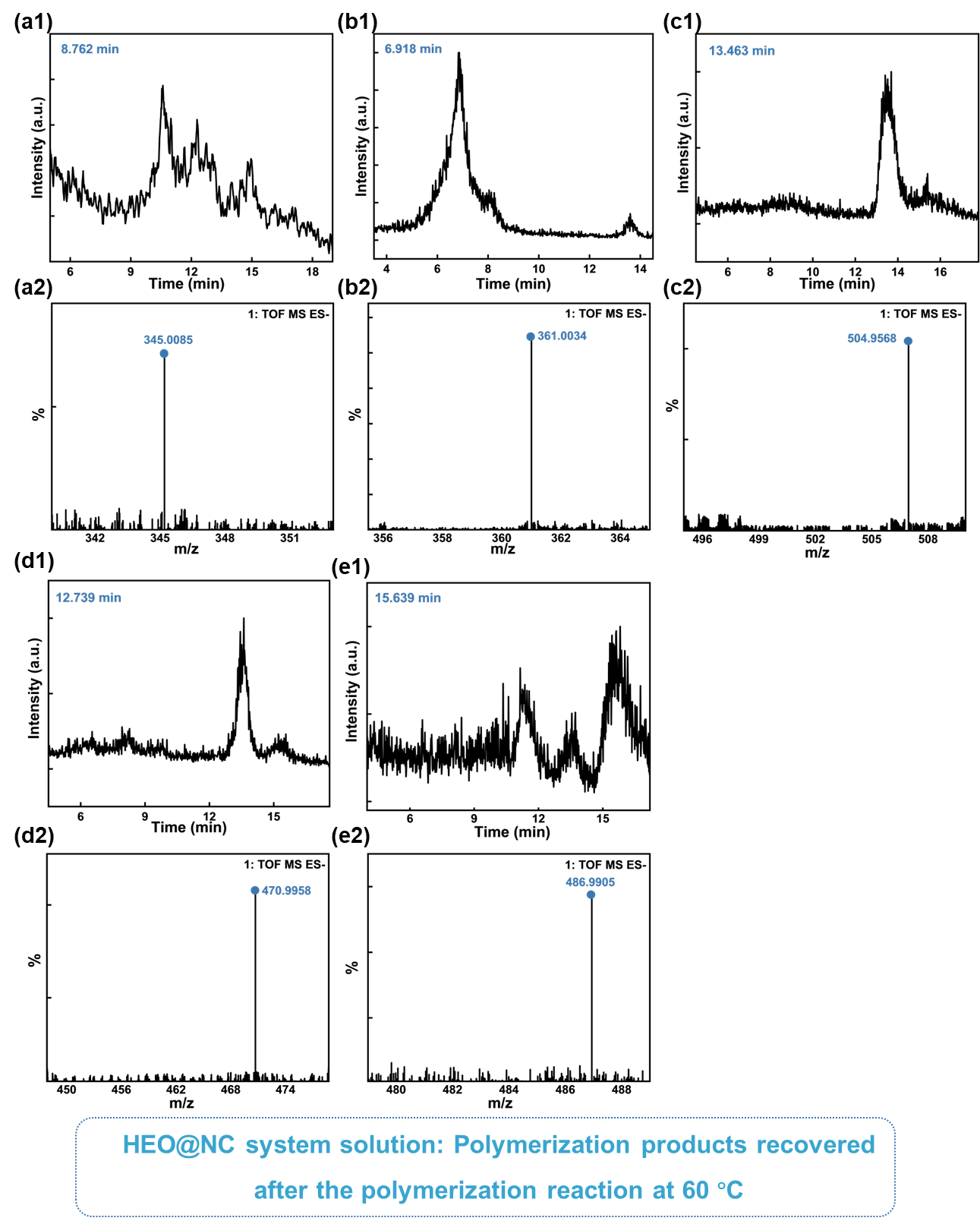


**Fig. S83 |** UPLC-QTOF-MS chromatograms of the polymerization products collected from the 4CP oxidation reaction at 60 °C on the HEO@NC system solution, and the corresponding molecular ion mass spectra of the chromatographic peaks.


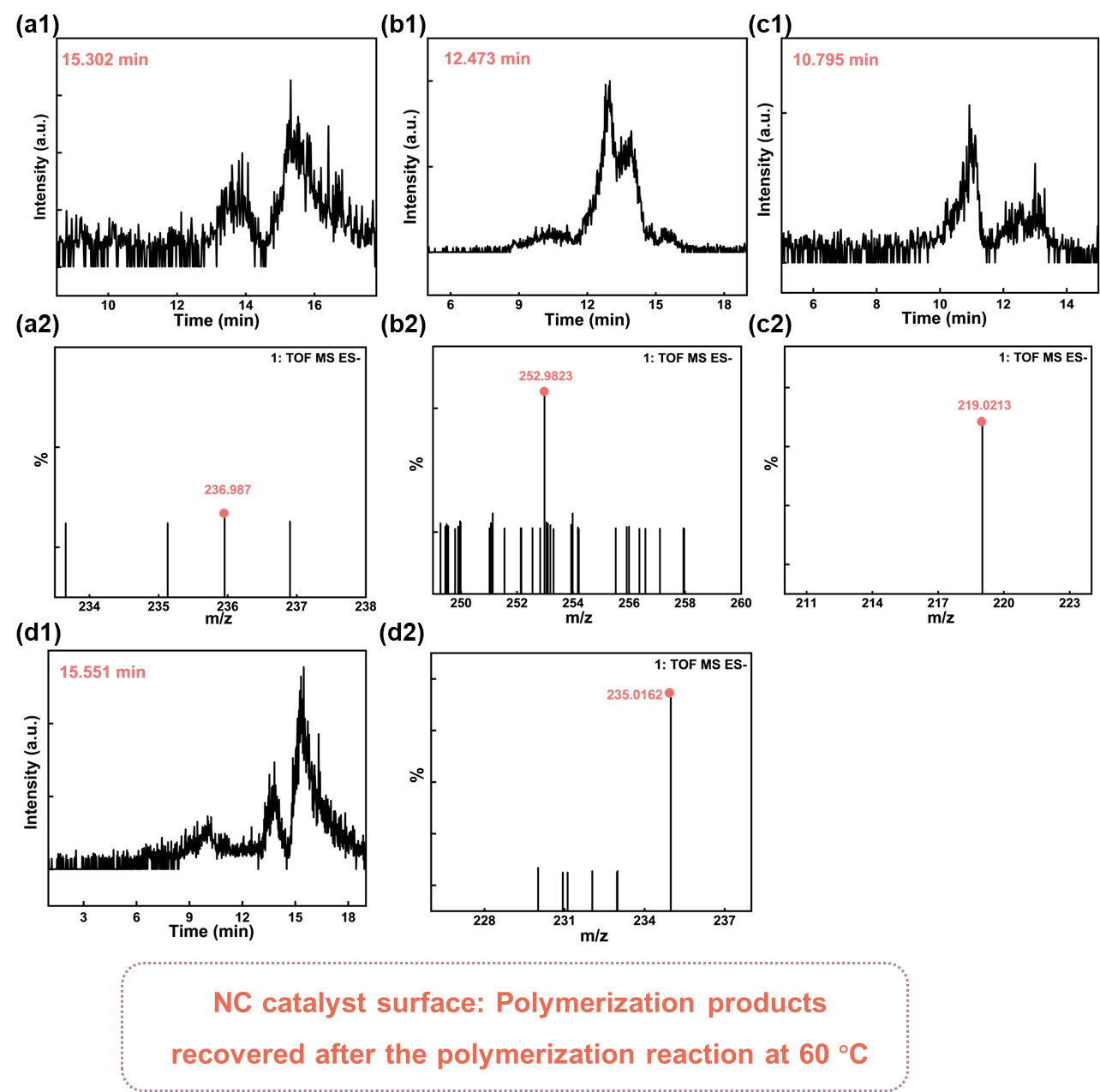


**Fig. S84 |** UPLC-QTOF-MS chromatograms of the polymerization products collected from the 4CP oxidation reaction at 60 °C on the surface of the NC catalyst, and the corresponding molecular ion mass spectra of the chromatographic peaks.


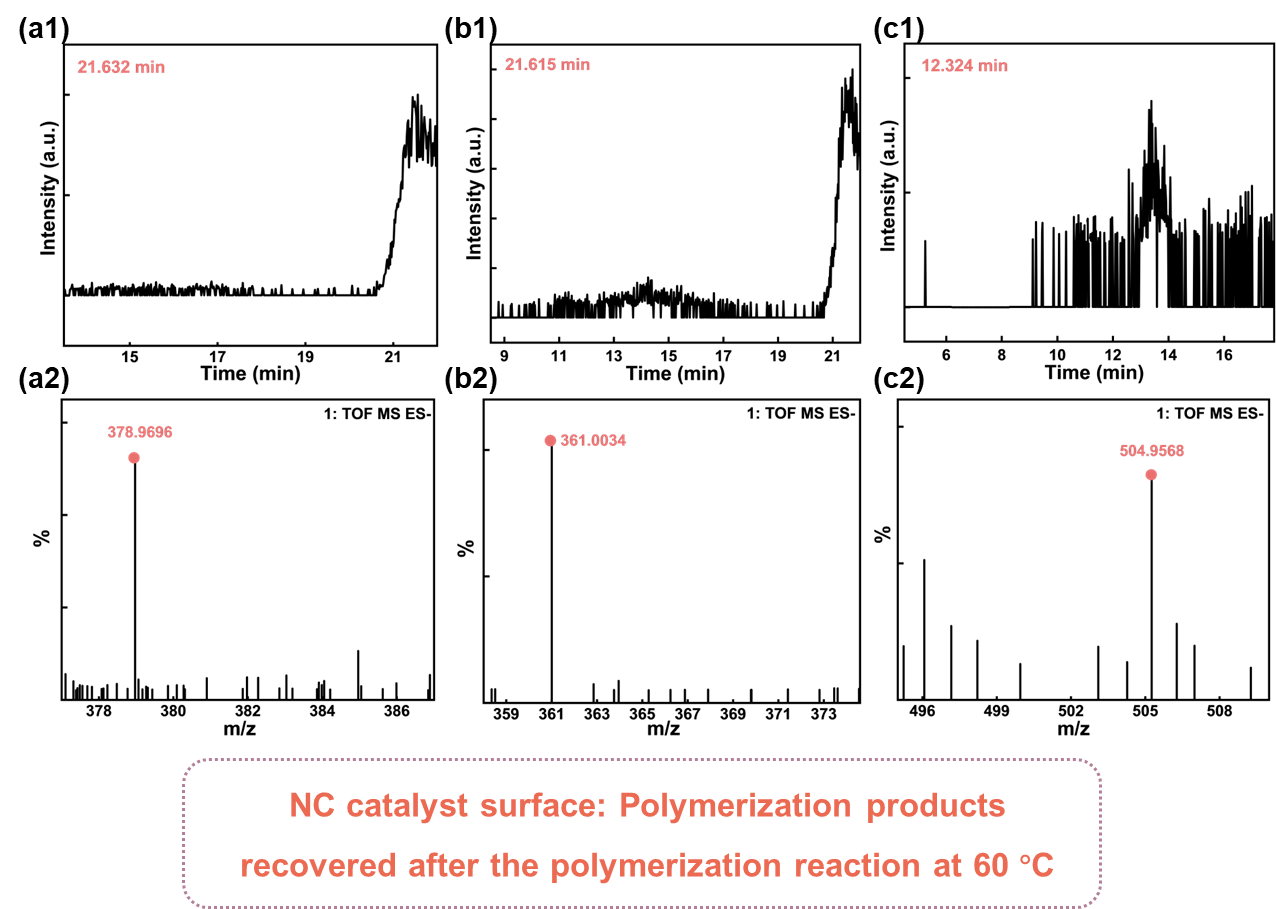


**Fig. S85 |** UPLC-QTOF-MS chromatograms of the polymerization products collected from the 4CP oxidation reaction at 60 °C on the surface of the NC catalyst, and the corresponding molecular ion mass spectra of the chromatographic peaks.


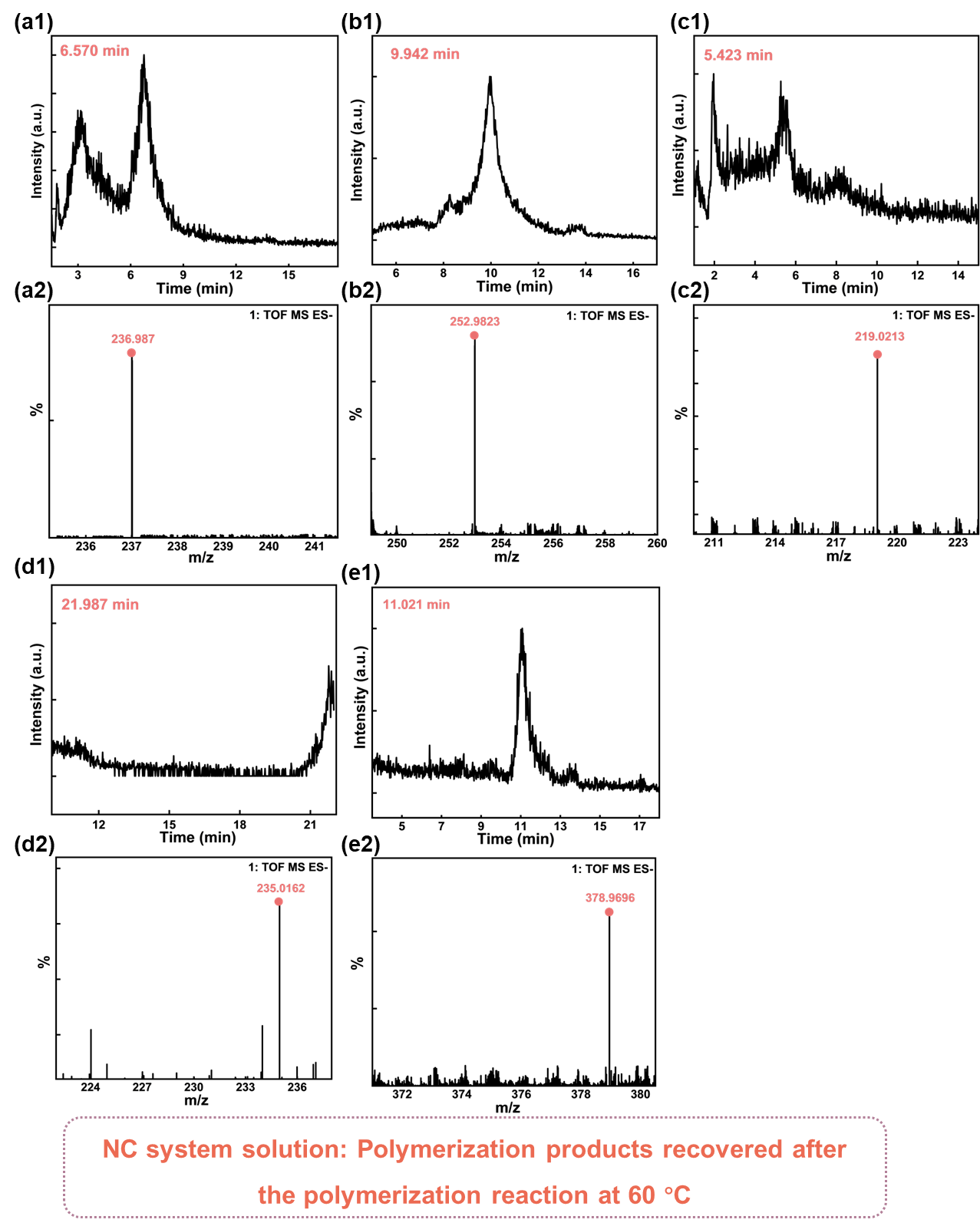


**Fig. S86 |** UPLC-QTOF-MS chromatograms of the polymerization products collected from the 4CP oxidation reaction at 60 °C on the NC system solution, and the corresponding molecular ion mass spectra of the chromatographic peaks.


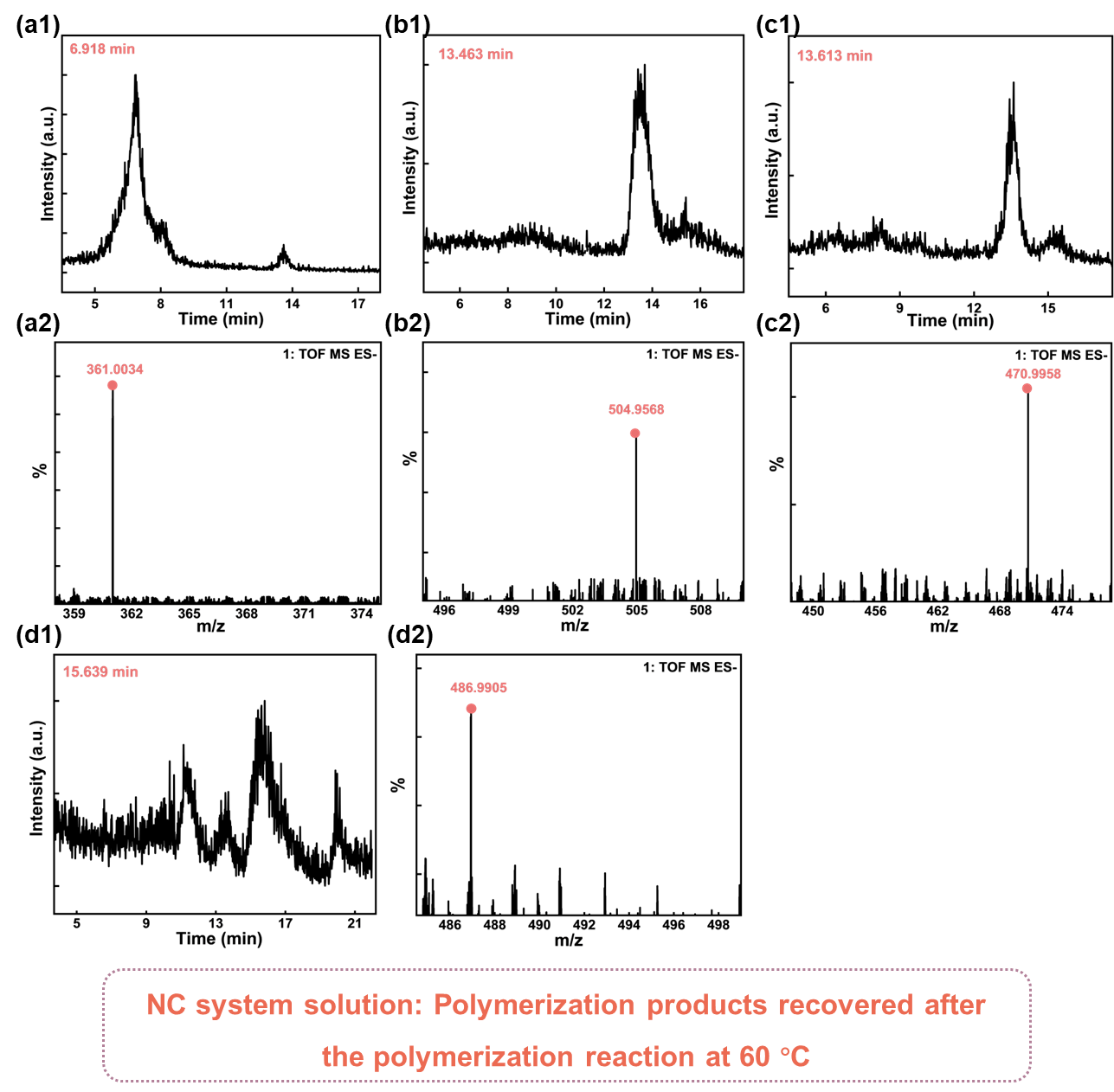


**Fig. S87 |** UPLC-QTOF-MS chromatograms of the polymerization products collected from the 4CP oxidation reaction at 60 °C on the NC system solution, and the corresponding molecular ion mass spectra of the chromatographic peaks.


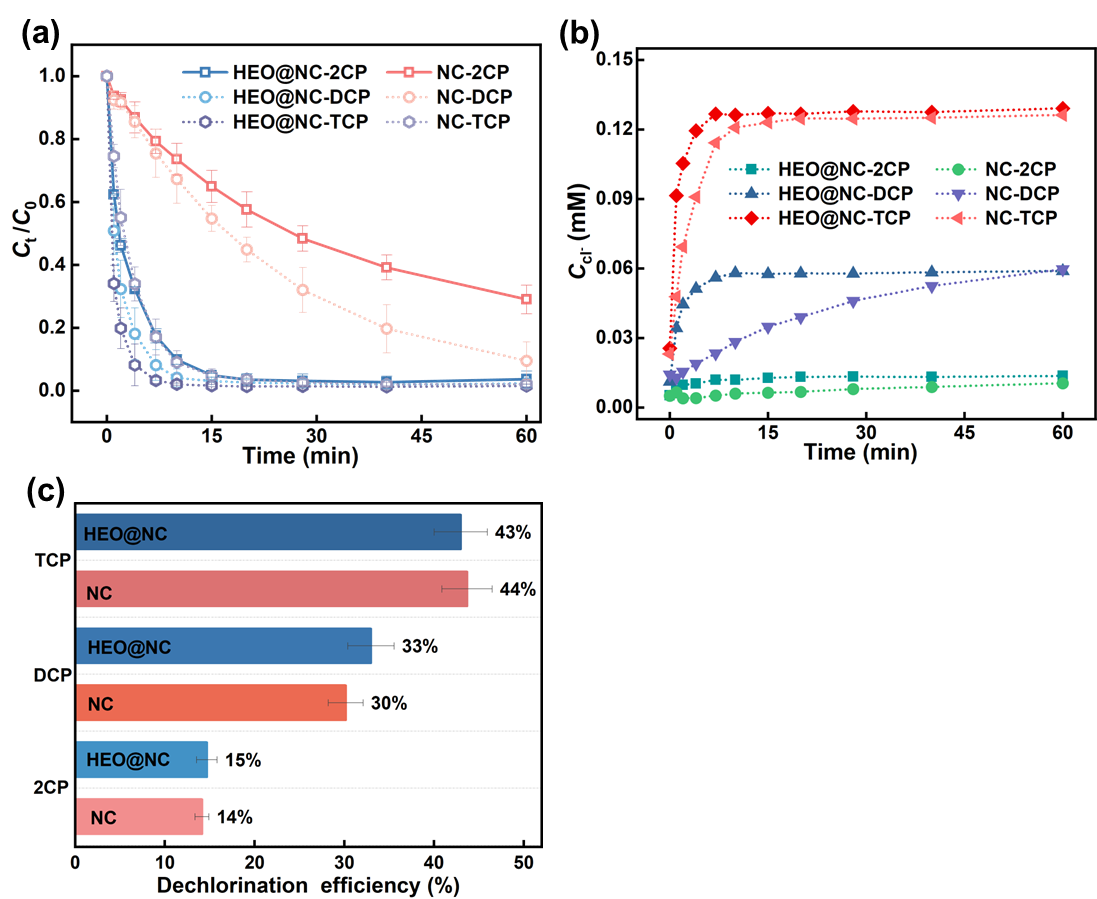


**Fig. S88 |** (a) Removal efficiencies of different chlorophenols. (b) The resulting dechlorination concentrations. (c) Comparative analysis of the dechlorination levels achieved by the different systems.


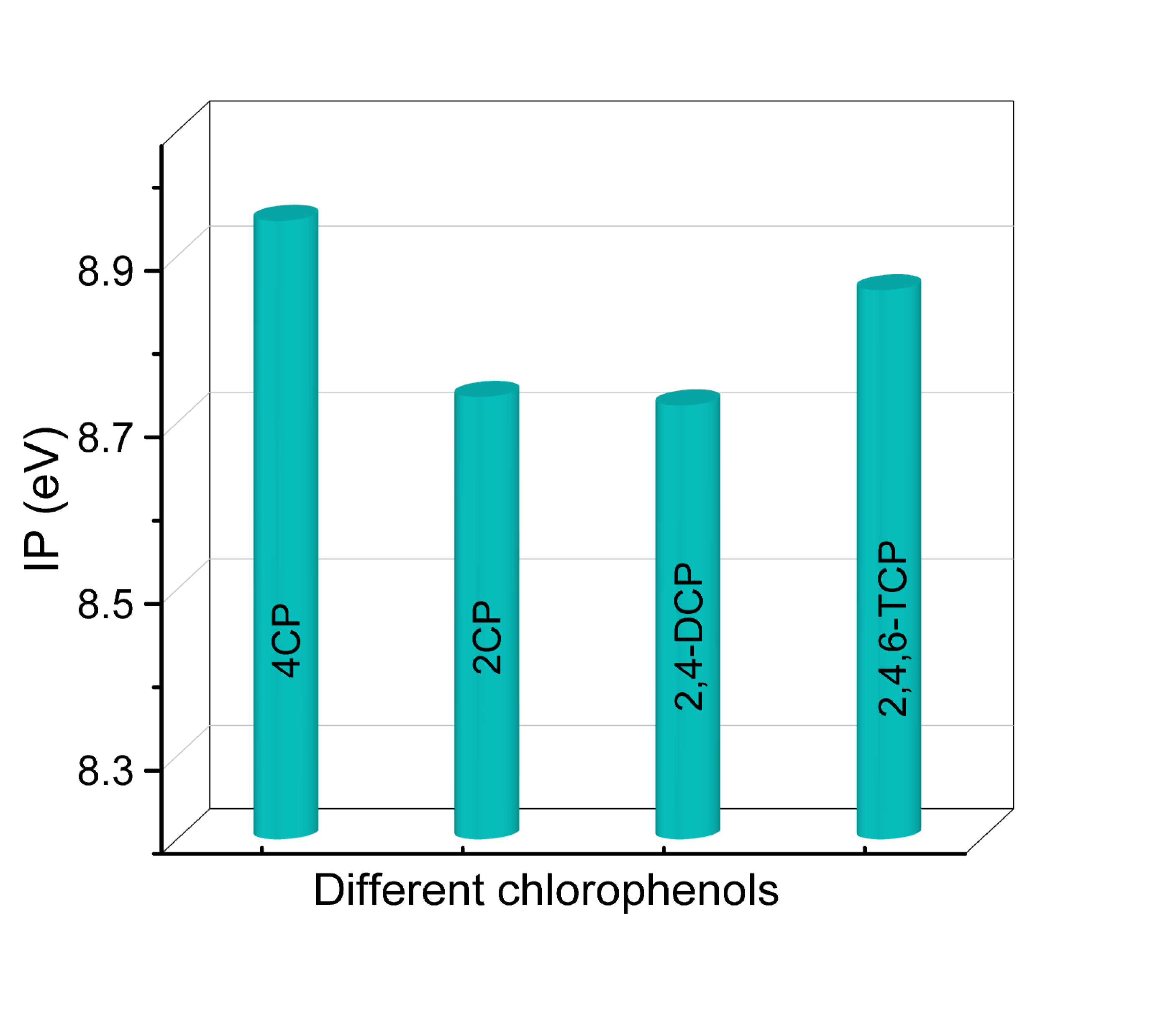


**Fig. S89 |** The First vertical IP of different chlorophenols.


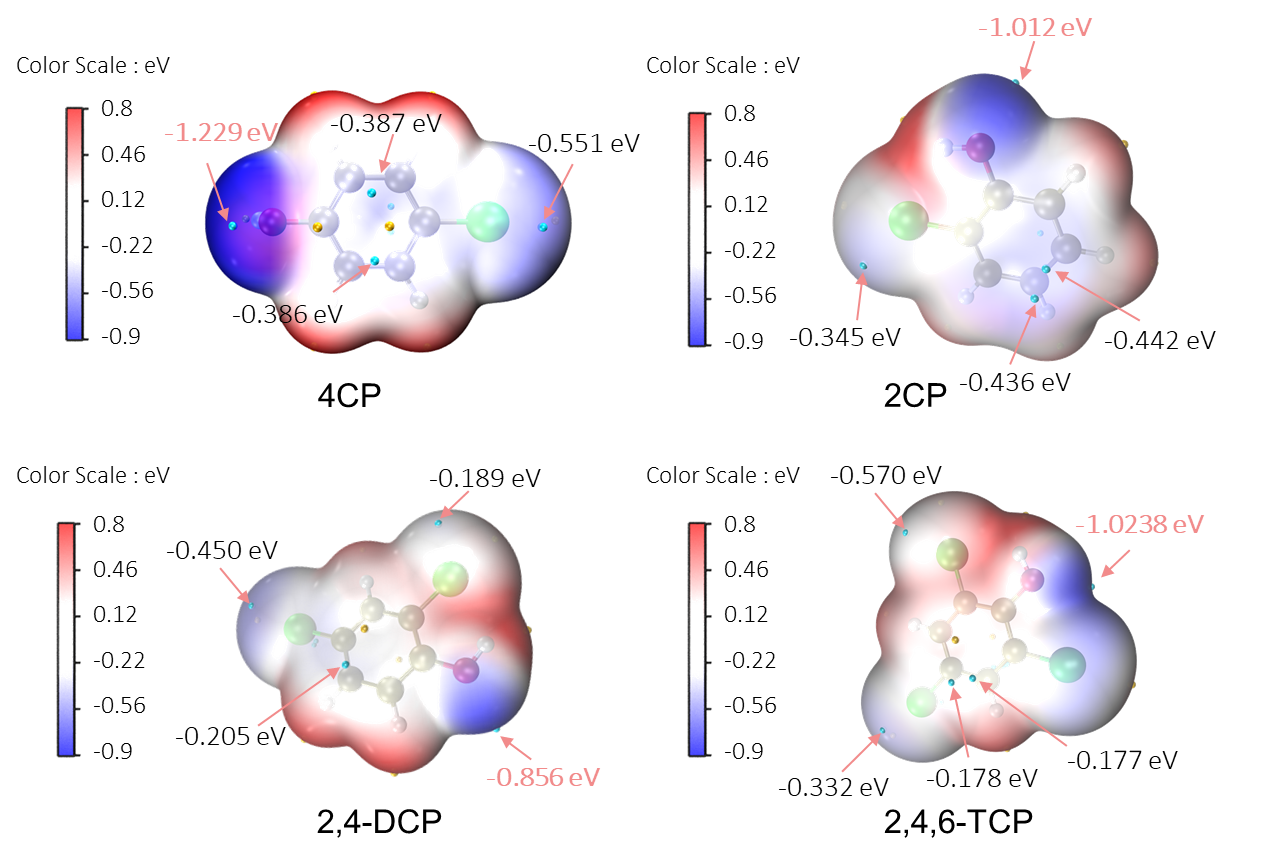


**Fig. S90 |** The electrostatic potential (ESP) distribution of different chlorophenols.


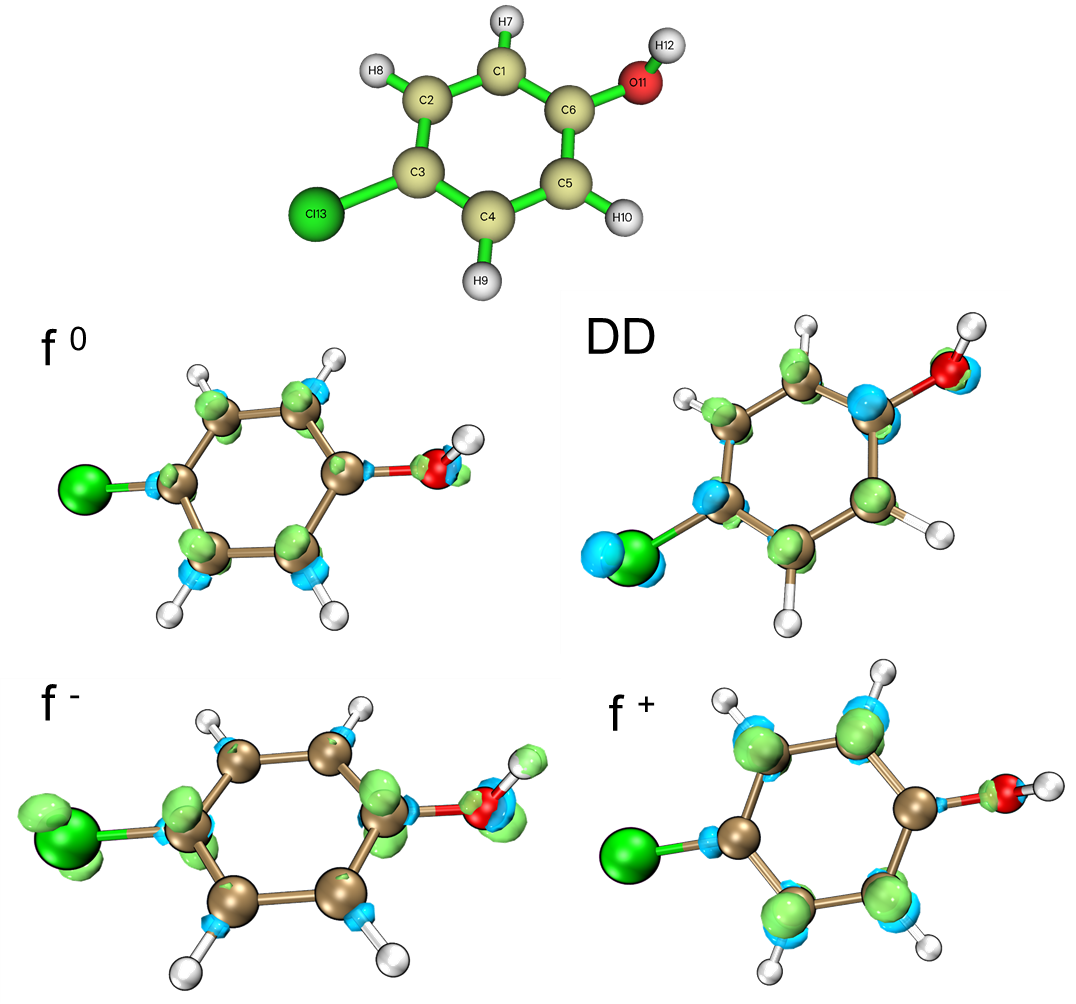


**Fig. S91 |** Visualized f^+^, f^⁻^, f^0^ and condensed dual descriptors (DD) Fukui functions values of 4CP and the numbering system. (The green surface represented the positive value of Fukui functions, and blue surface represented the negative value. The brown, white, red, and green balls represented C, H, O, and Cl atoms, respectively.)

The calculated local electrophilicity/nucleophilicity indices, including the f⁻ and CDD, reveal that the para-chlorine atom exhibits the highest f⁻ and CDD values (with the trend f⁻_para-Cl_: 4CP > DCP > TCP > 2CP), indicating its high reactivity toward electrophilic species such as ^•^OH_ads_ and monomeric organic radicals. Notably, the higher degree of dechlorination observed for TCP compared to DCP is attributed to the greater number of chlorine substitution sites available in the TCP molecule. In aromatic systems, the hydroxyl group (–OH), as a strong electron-donating substituent, significantly enhances the electron density at the para-carbon via resonance and inductive effects, thereby facilitating ^•^OH_ads_-induced chlorine substitution at that position. In contrast, the reactivity of other carbon atoms on the ring is much lower than that of the chlorine-substituted site, making them less susceptible to similar substitution reactions.


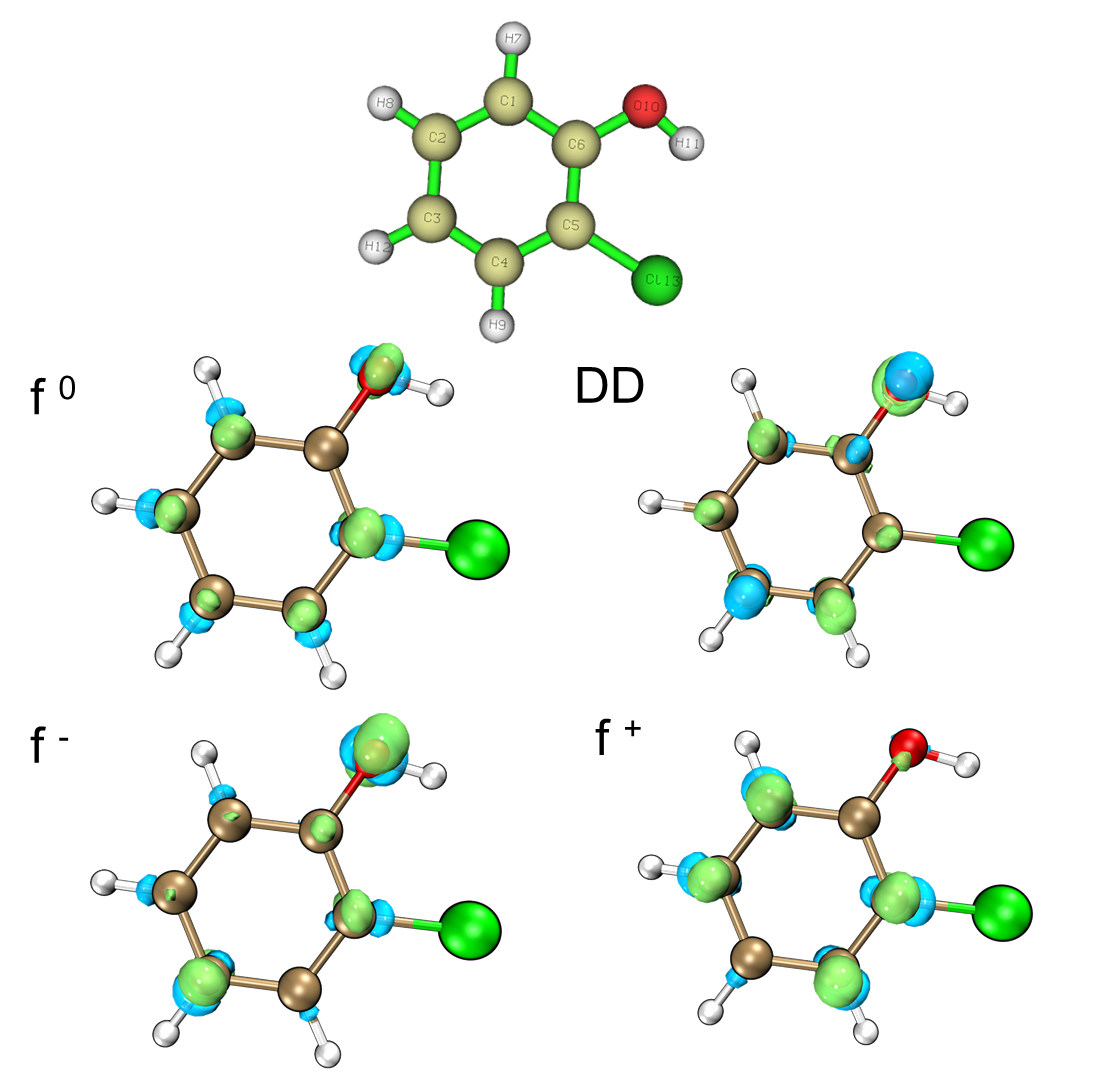


**Fig. S92 |** Visualized f^+^, f^-^, f^0^ and condensed dual descriptors (DD) Fukui functions values of 2CP and the numbering system. (The green surface represented the positive value of Fukui functions, and blue surface represented the negative value. The brown, white, red, and green balls represented C, H, O, and Cl atoms, respectively.)


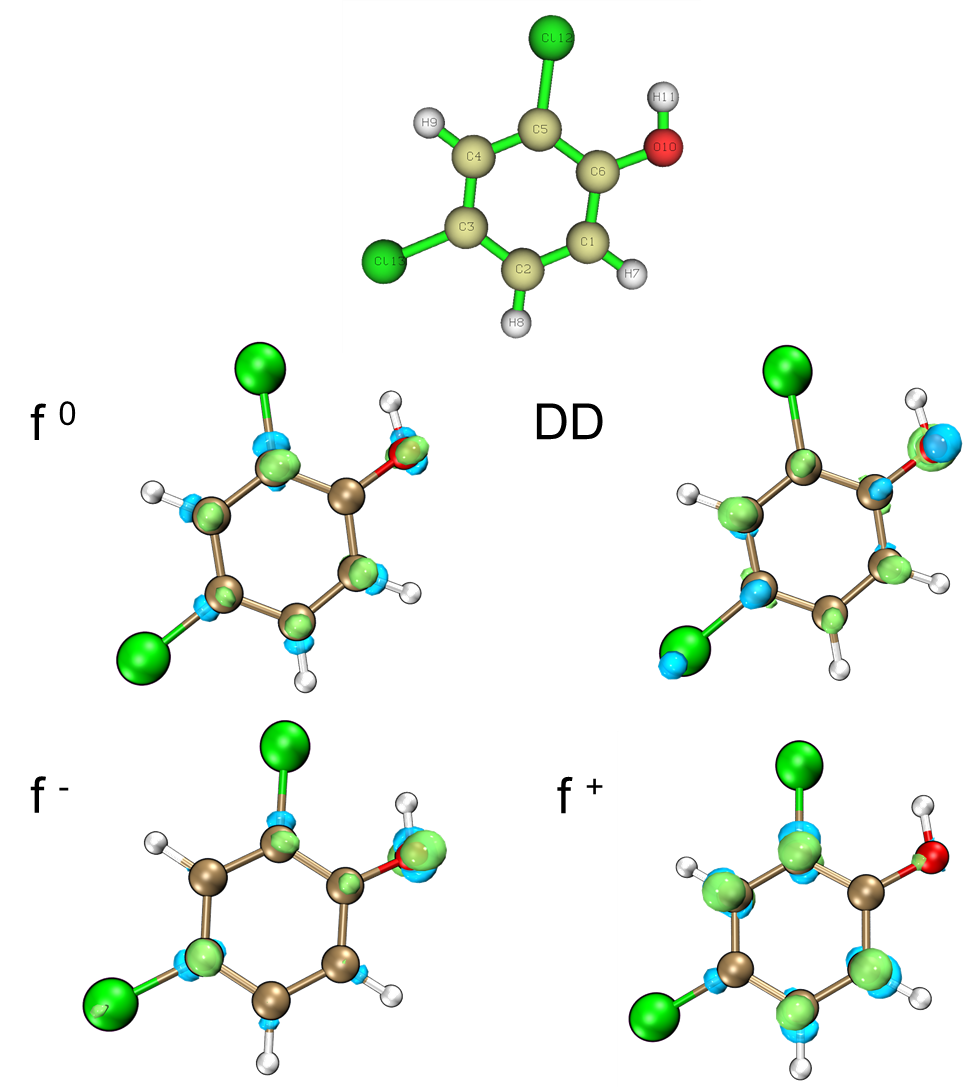


**Fig. S93 |** Visualized f^+^, f^-^, f^0^ and condensed dual descriptors (DD) Fukui functions values of 2,4-DCP and the numbering system. (The green surface represented the positive value of Fukui functions, and blue surface represented the negative value. The brown, white, red, and green balls represented C, H, O, and Cl atoms, respectively.)


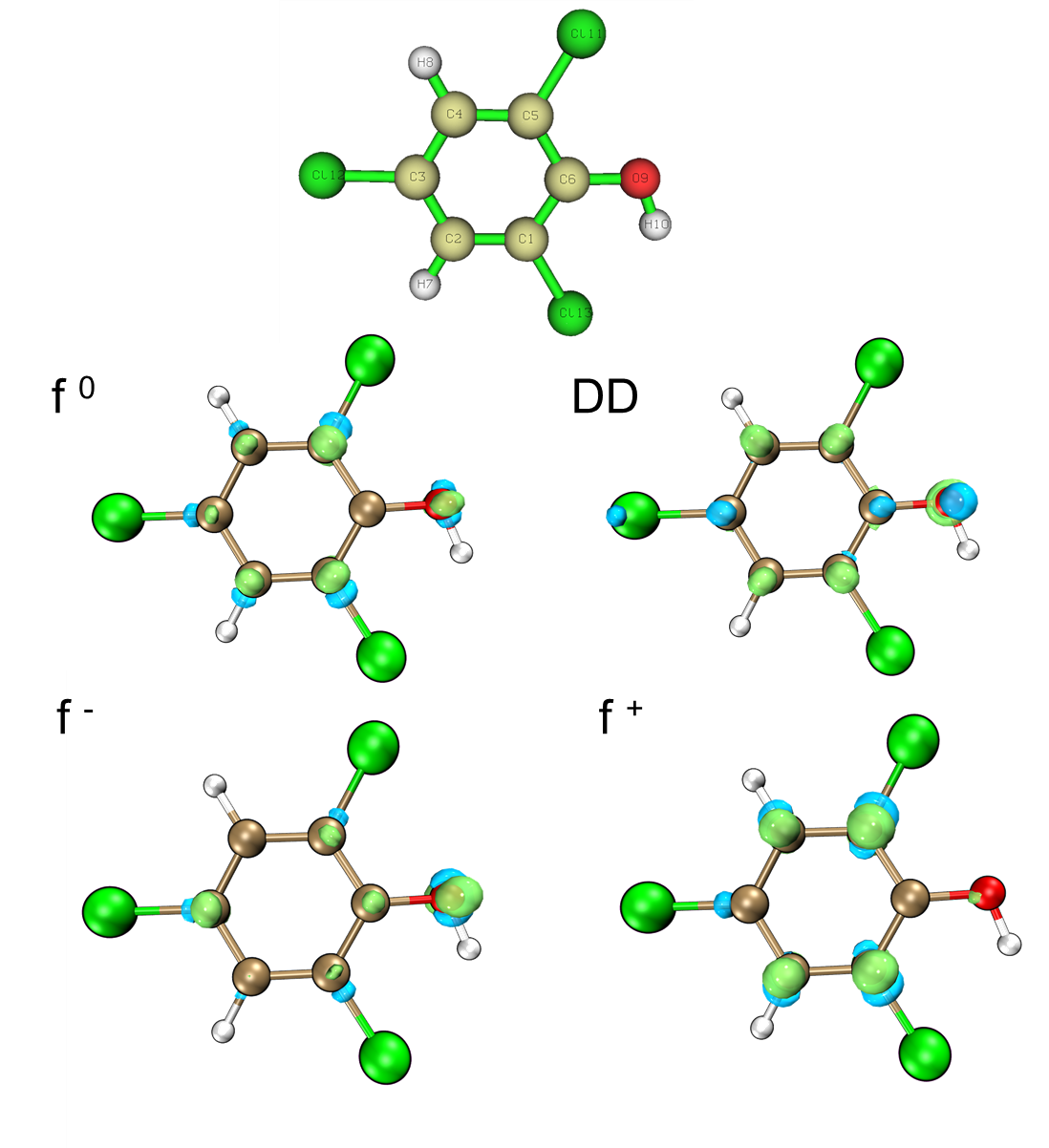


**Fig. S94 |** Visualized f^+^, f^-^, f^0^ and condensed dual descriptors (DD) Fukui functions values of 2,4,6-TCP and the numbering system. (The green surface represented the positive value of Fukui functions, and blue surface represented the negative value. The brown, white, red, and green balls represented C, H, O, and Cl atoms, respectively.)


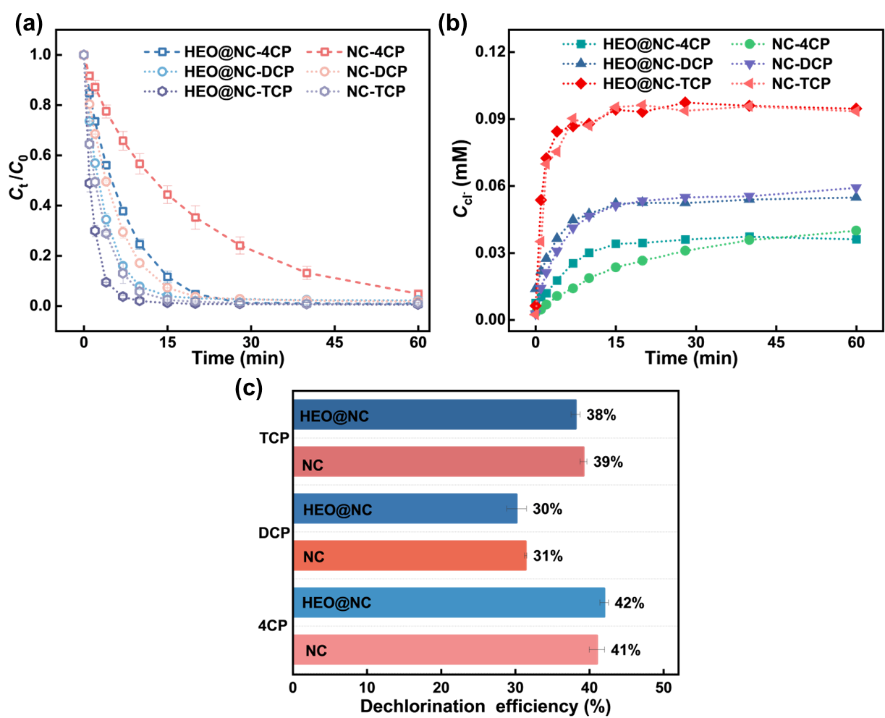


**Fig. S95 |** (a) Removal efficiencies of different chlorophenols by PDS activation. (b) The resulting dechlorination concentrations. (c) Comparative analysis of the dechlorination levels achieved by the different systems. Dosage: [Pollutants]_0_: 0.1 mM, PDS: 0.5 mM, reaction solution: 50 mL, catalyst: 0.1 g/L.

The dechlorination efficiency for the different pollutants maintained the same order and strong correlation with their computed electronic properties (e.g., Fukui index), despite the change in oxidant. This result robustly confirms that the dechlorination behavior is indeed governed by the intrinsic electronic structure of the pollutant molecules, rather than being specific to a particular oxidant.


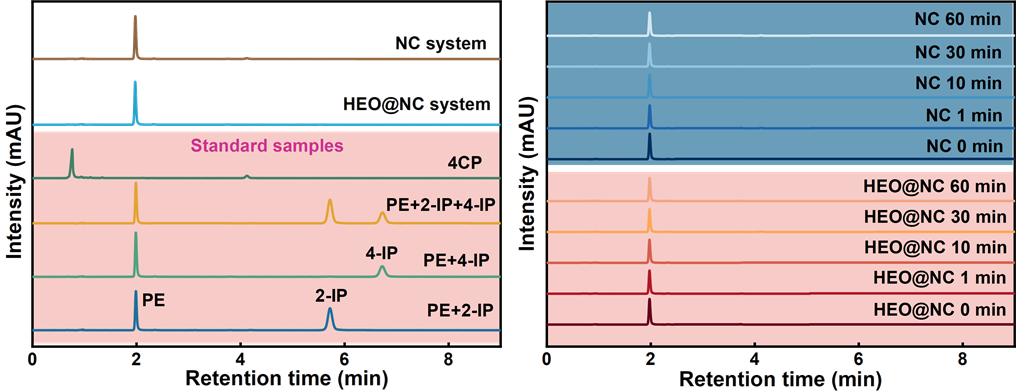


**Fig. S96 |** Detection of HOI generated in HEO@NC-PI system using phenol as the trapping agent.


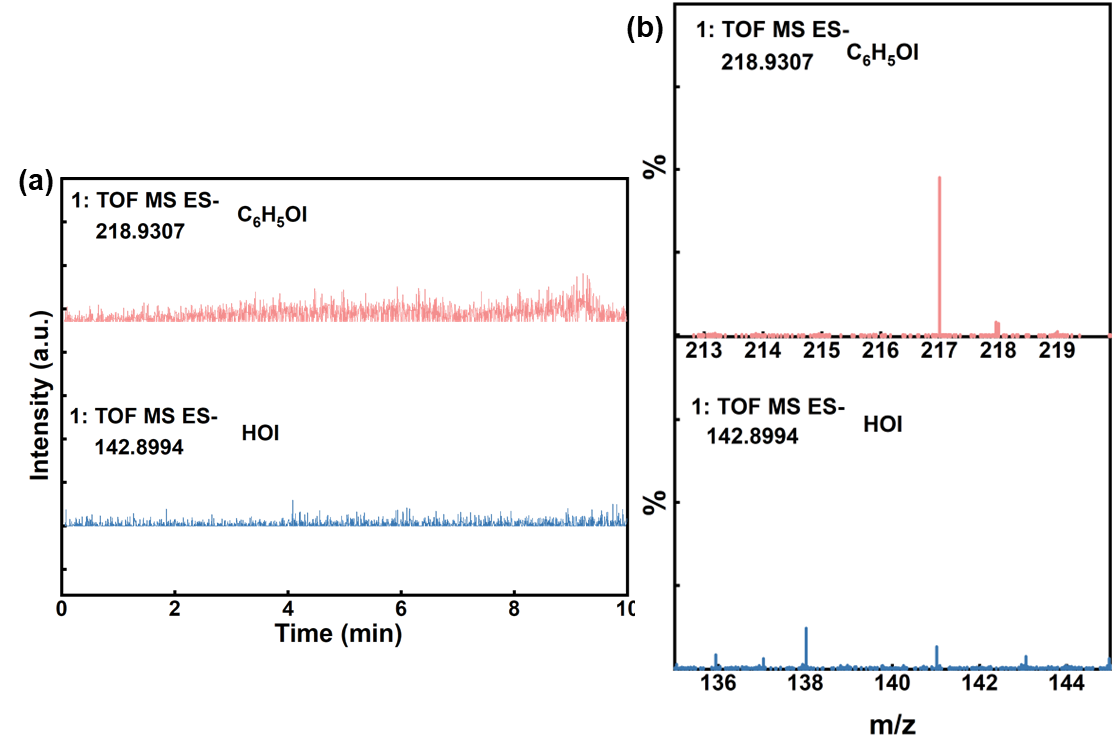


**Fig. S97 |** Detection of HOI generated in HEO@NC-PI system by UPLC-QTOF-MS.


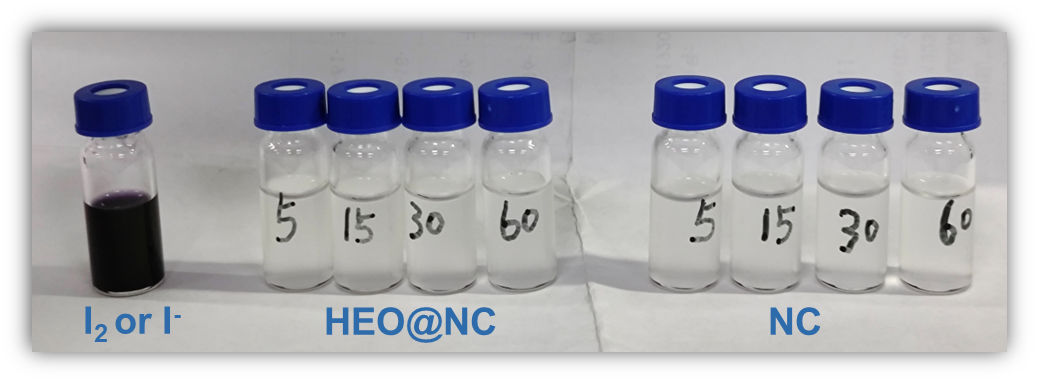


**Fig. S98 |** Detection of I_2_ and I_3_^−^ HEO@NC-PI system.


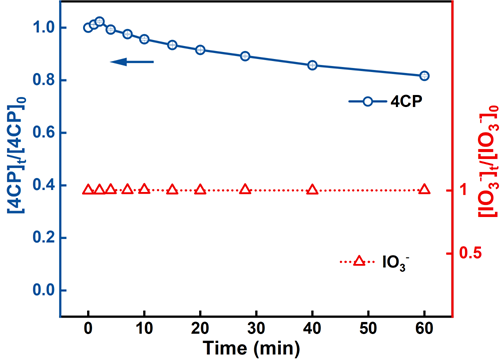


**Fig. S99 |** IO_3_^−^ concentration profile in the presence HEO@NC-IO_3_ system. Dosage: [4CP]_0_: 0.1 mM, IO_3_^−^: 0.5 mM, reaction solution: 50 mL, catalyst: 0.1 g/L, reaction time: 60 min.


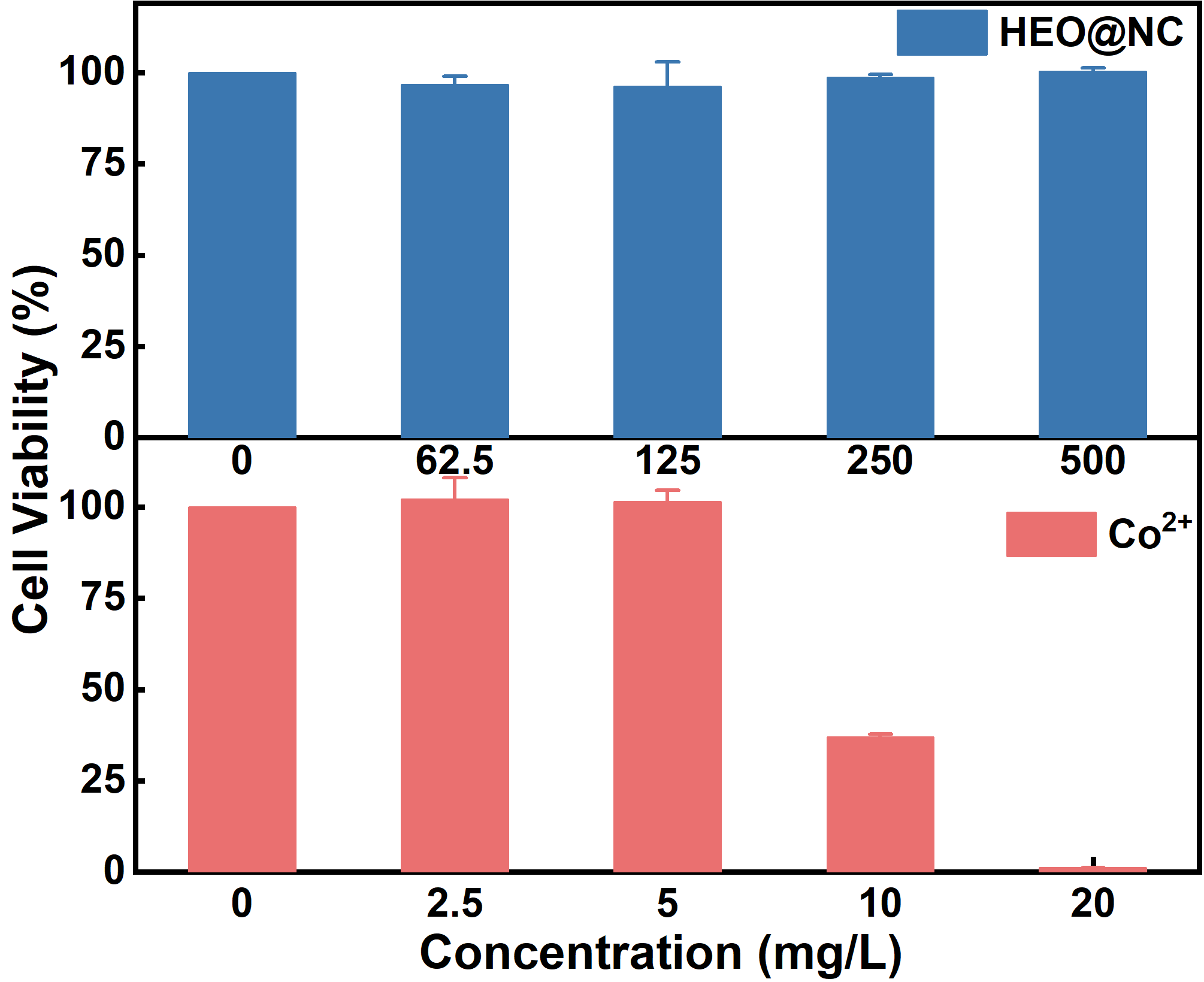


**Fig. S100 |** Cell viability of 16HBE under different concentrations of HEO@NC and Co^2+^. Cell culture time: 48 h.

**Fig. S101 |** Cell viability of 16HBE under different concentrations of HEO@NC and Co^2+^, and cells in the culture medium were observed under a microscope. Cell culture time: 48 h.

Exposure to Co^2+^ significantly compromised the viability of 16HBE cells. Under microscopic examination, clear morphological disruptions were observed, accompanied by a substantial increase in cell death.

**Fig. S102 |** Representative images of zebrafish development (a) and fluorescent ROS imaging (b) in NC and HEO@NC systems. (c) Survival rate (144 hpf) and hatchability (72hpf) of zebrafish exposed to the NC and HEO@NC systems.

**Fig. S103 |** HEO@NC and NC catalytic removal rate of 4CP with interference of salts and humic acid. Dosage: [4CP]_0_: 0.1 mM, PI: 0.5 mM, reaction solution: 50 mL, catalyst: 0.1 g/L, reaction time: 60 min.

However, when the concentration increased to 5 mM, HCO_3_^−^ slowed the removal rate to 85.7%, likely due to rate changes induced by pH variations. Similarly, HEO@NC achieved a removal rate exceeding 99.1% at 1 mg/L. However, when the DOM concentration was increased to 5 mg/L, it significantly affected the 4CP removal rate decreased to 79.7%. In contrast, NC consistently showed a noticeable impact on the removal process.

**Fig. S104 |** Comparison of the distribution ratios of polymeric products categorized by degree of polymerization in the NC-PI and HEO@NC-PI systems under (a) different initial pH conditions and (b) coexisting ions

We employed UPLC-QTOF-MS to comparatively analyze 4CP polymerization products of varying degrees in the HEO@NC-PI and NC-PI systems at initial pH values of 3, 7, and 9, as well as in the presence of Cl⁻ ions. The results indicate that the distribution of 4CP polymerization products is influenced by pH: under acidic conditions, polymerization is primarily controlled by the PCET process and the formation of phenoxy radicals, while under alkaline conditions, changes in the speciation and activation efficiency of the PI oxidant influence the polymerization degree and product distribution. Moreover, coexisting Cl⁻ ions exert negligible impact on the polymerization behavior, suggesting that the catalytic system possesses strong resistance to ionic interference in realistic aqueous matrices and highlighting its potential for practical wastewater treatment applications.

**Fig. S105 |** EEM spectra of collected coal chemical industry wastewater treatment by HEO@NC–PI system at different time intervals.

**Fig. S106 |** Physical diagram of the continuous-flow reactor.

**Fig. S107 |** Performance of regenerated HEO@NC treated with different elution regeneration methods after long-term stability tests.

Simple solvent washing was unable to fully restore the catalytic activity, likely because polymeric products are strongly adsorbed at the active sites through enhanced chemical interactions with polymer chain ends, leading to persistent blockage of active sites. In contrast, the catalytic activity was effectively recovered after annealing under appropriate conditions, indicating that thermal treatment can completely decompose and remove organic residues, thereby re-exposing the blocked active sites. These results demonstrate that catalyst deactivation is primarily caused by surface fouling rather than irreversible structural damage.

**Fig. S108 |** Structural characterization of the polymerization products generated during 4CP oxidation: (a) photographic images, (b) FTIR spectrum of characteristic aromatic and functionalities, and (c) thermogravimetric analysis of thermal stability.

TG and FTIR analyses further verify that the polymeric products accumulated during the reaction possess excellent thermal stability and well-preserved structural integrity, indicating that these species can withstand the applied reaction conditions without significant structural decomposition or fragmentation.

**Table S1 |** Metal atomic ratios of HEO@NC characterized by ICP.

| sample | Pb | Bi | Pt | Co | Ni |
| --- | --- | --- | --- | --- | --- |
| Content ratio | 10 | 15 | 12 | 5 | 3 |

**Table S2 |** The CHON element ratios of HEO@NC and NC characterized by Organic Element Analyze.

| Sample | C | H | O | N |
| --- | --- | --- | --- | --- |
| HEO@NC | 69.36 | 2.55 | 8.24 | 11.98 |
| NC | 70.45 | 2.75 | 0.87 | 15.03 |

Table S3 | The concentration of residual metal ions in the solution of HEO@NC-PI system. Dosage: [4CP]_0_: 0.1 mM, PI: 0.5 mM, reaction solution: 50 mL, catalyst: 0.1 g/L, reaction time: 60 min.

| Ion (mg/L) | Pb^2+^ | Bi^2+^ | Pt^2+^ | Co^2+^ | Ni^2+^ |
| --- | --- | --- | --- | --- | --- |
| HEO@NC | 0.002 | 0.02 | 0.008 | 0.014 | 0.014 |

Table S4 | Comparison of catalytic reactivity of HEO@NC catalyst with previous reported catalysts during PI activation.

| Catalyst | Catalyst dosage (g/L) | Pollutant (mM) | PI (mM) | Removal  efficiency | Rate constant  (min^-1^) | normalized k value ^a^ | Reference |
| --- | --- | --- | --- | --- | --- | --- | --- |
| HEO@NC | 0.1 | 4CP (0.1) | 0.24 | 100% (25min) | 0.198 | 0.825 | This study |
| α-Fe_2_O_3_ | 0.4 | 4CP (0.1) | 1.0 | 100% (50min) | 0.1015 | 0.025 | 8 |
| S-Fe^0^ | 0.1 | SDZ (0.06) | 1.0 | 97.64% (5min) | ~3.161 | 0.379 | 9 |
| N-rGO-CoSA | 0.2 | 4CP (0.1) | 1 | 95% (12min) | 0.241 | 0.121 | 10 |
| Fe, N-C | 0.5 | SDZ (0.04) | 5 | ~98% (90min) | 0.0586 | 9.38×10^-4^ | 11 |
| Fe/SCN | 0.5 | 4CP (0.1) | 2.5 | 100% (30min) | 0.189 | 0.015 | 12 |
| BM-SBC/Mn_2_O_3_ | 1 | SMX (0.02) | 1 | ~95% (30min) | 0.09 | 1.8×10^-3^ | 13 |
| Fe@N-C | 0.05 | SIZ (0.019) | 0.5 | ~90% (10min) | 0.196 | 0.149 | 14 |

^a^ The Specific activity (normalized k value) was calculated through dividing the observed rate constant of pollutant by the catalyst dosage and PI concentration, followed by multiplying organic contaminant concentration.

Table S5 | The concentration of metal ion in the solution of ions-PI system. Dosage: [4CP]_0_: 0.1 mM, PI: 0.5 mM/L, reaction solution: 50 mL, reaction time: 60 min.

| Ion (mg/L) | Pb | Bi | Pt | Co | Ni |
| --- | --- | --- | --- | --- | --- |
| equivalents Co^2+^ |  |  |  | 0.11 |  |
| equivalents Ni^2+^ |  |  |  |  | 0.13 |
| equivalents Pt^2+^ |  |  | 0.42 |  |  |
| equivalents Pb^2+^ | 0.35 |  |  |  |  |
| equivalents Bi^2+^ |  | 0.52 |  |  |  |

**Table S6 |** The interaction energy between NC or HEO@NC and adsorbed 4CP or PI.

| Configuration | Interaction energy (eV) |
| --- | --- |
| PI on HEO@NC | -5.815 |
| PI on NC | -3.381 |
| 4CP on HEO@NC | -1.767 |
| 4CP on NC | -0.501 |

**Table S7 |** Summary of DFT calculation results for PI adsorption on HEO@NC and NC.

| Configuration | $l_{O-O}$ (Å) | $\text{E}_{\text{ads}}$ (eV) | Q (*e*) |
| --- | --- | --- | --- |
| Free PI | 1.78 | —— | —— |
| NC (PI*) | 2.16 | -3.381 | 0.93 |
| HEO@NC (PI*) | 2.15 | -5.815 | 1.53 |

Table S8 | Comparison of the apparent PI utilization efficiency of different systems.

| Systems | [4CP]  (mM) | TOC removal  (%) | Given *e*^−^  (mM) ^a^ | Real PI consumption  (mM) | Obtained *e*^−^ (mM) ^b^ | PI theoretical consumption ^c^ | PI utilization  (%) ^d^ |
| --- | --- | --- | --- | --- | --- | --- | --- |
| HEO@NC/PI | 0.1 | 87.40 | 2.272 | 0.2530 | 0.506 | 1.136 | 449.15 |
| NC/PI | 0.1*51.8% | 26.76 | 0.360 | 0.0704 | 0.1408 | 0.18 | 255.68 |

^a^ The given electron equivalent of pollutant mineralization is calculated according to the mineralization rate. Take HEO@NC-PI as an example, the given *e*^−^ = [4CP] × TOC removal × 26 = 0.1 × 87.4% × 26 = 2.272 mM.

C_6_H_5_ClO + 11H_2_O − 26e^-^ → 6CO_2_ + Cl^-^ + 27H^+^

^b^ This value was calculated according to the actual dosage of oxidant (PI). One mole of PI accepts 2 moles of *e*^−^ in the reaction process. For HEO@NC-PI system as an example, the *e*^−^ equivalence of oxidant was calculated from: [Real PI consumption]×2 = 0.253×2 = 0.506 mM

^c^ The PI theoretical consumption value was calculated based on the PI consumption required for complete mineralization. For HEO@NC-PI system as an example, the PI theoretical consumption value was calculated from: given *e*^−^/2 = 2.272/2 = 1.136 mM

^d^ The apparent PI utilization efficiency is calculated by the ratio of the PI theoretical consumption/real PI consumption. In the HEO@NC-PI system, the apparent PI utilization efficiency is calculated as (1.136/0.253) × 100% = 449.15%. A similar phenomenon of high apparent PI utilization efficiency has also been reported in previous studies in heterogeneous systems^15–17^.

Table S9 | 4CP oxidation products in HEO@NC-PI and NC-PI catalyst surface and system solution

| Name^a^ | Chemical formula | structural formula | M-H  (m/z) | HEO@NC | | NC | |
| --- | --- | --- | --- | --- | --- | --- | --- |
|  |  |  |  | solution | catalyst | solution | catalyst |
| P128 | C_6_H_5_ClO |  | 126.9951 | 🗸 | 🗸 | 🗸 | 🗸 |
| P220 | C_12_H_9_ClO_2_ |  | 219.0213 | 🗸 | 🗸 | 🗸 | 🗸 |
| P236 | C_12_H_9_ClO_3_ |  | 235.0162 | 🗸 | 🗸 | 🗸 | 🗸 |
| P238 | C_12_H_8_Cl_2_O |  | 236.9874 | 🗴 | 🗸 | 🗴 | 🗸 |
| P254 | C_12_H_8_Cl_2_O_2_ |  | 252.9823 | 🗸 | 🗸 | 🗸 | 🗸 |
| P312 | C_18_H_13_ClO_3_ |  | 311.0475 | 🗴 | 🗸 | 🗴 | 🗸 |
| P346 | C_18_H_12_Cl_2_O_3_ |  | 345.0085 | 🗸 | 🗸 | 🗸 | 🗸 |
| P362 | C_18_H_12_Cl_2_O_3_ |  | 361.0034 | 🗴 | 🗸 | 🗴 | 🗸 |
| P380 | C_18_H_11_Cl_3_O_3_ |  | 378.9696 | 🗸 | 🗸 | 🗸 | 🗸 |
| P438 | C_24_H_16_Cl_2_O_4_ |  | 437.0347 | 🗴 | 🗸 | 🗴 | 🗴 |
| P472 | C_24_H_15_Cl_3_O_4_ |  | 470.9958 | 🗸 | 🗸 | 🗸 | 🗸 |
| P488 | C_24_H_15_Cl_3_O_5_ |  | 486.9906 | 🗴 | 🗸 | 🗴 | 🗸 |
| P506 | C_24_H_14_Cl_4_O_4_ |  | 504.9568 | 🗸 | 🗸 | 🗸 | 🗴 |
| P564 | C_30_H_19_Cl_3_O_5_ |  | 563.022 | 🗴 | 🗸 | 🗴 | 🗴 |
| P598 | C_30_H_18_Cl_4_O_5_ |  | 596.983 | 🗴 | 🗸 | 🗴 | 🗴 |
| P614 | C_30_H_18_Cl_4_O_6_ |  | 612.9779 | 🗴 | 🗸 | 🗴 | 🗴 |
| P632 | C_30_H_17_Cl_5_O_5_ |  | 630.9278 | 🗴 | 🗸 | 🗴 | 🗴 |
| P758 | C_36_H_20_Cl_6_O_6_ |  | 756.9313 | 🗴 | 🗸 | 🗴 | 🗴 |
| P884 | C_42_H_23_C_l7_O_7_ |  | 882.9185 | 🗴 | 🗸 | 🗴 | 🗴 |

^a^ Blue and purple color font indicate polymer-mediated dechlorination products and surface hydroxyl radical-mediated oxidative dechlorination products, respectively.

Table S10 | Hirshfeld charges, condensed Fukui functions, condensed dual descriptors and local electrophilicity/nucleophilicity index of 4CP.

| Atom | q(N) | q(N+1) | q(N-1) | f^-^ | f^+^ | f^0^ | CDD | Electrophilicity | Nucleophilicity |
| --- | --- | --- | --- | --- | --- | --- | --- | --- | --- |
| 1(C) | -0.0468 | -0.1722 | 0.0211 | 0.0679 | 0.1254 | 0.0966 | 0.0575 | 0.09024 | 0.15248 |
| 2(C) | -0.0445 | -0.166 | 0.0201 | 0.0646 | 0.1216 | 0.0931 | 0.057 | 0.08749 | 0.14506 |
| 3(C) | 0.0118 | -0.028 | 0.1026 | 0.0908 | 0.0398 | 0.0653 | -0.0509 | 0.02868 | 0.20385 |
| 4(C) | -0.0445 | -0.1664 | 0.0201 | 0.0646 | 0.122 | 0.0933 | 0.0574 | 0.08778 | 0.14503 |
| 5(C) | -0.0467 | -0.1717 | 0.0212 | 0.0679 | 0.125 | 0.0964 | 0.057 | 0.08993 | 0.1525 |
| 6(C) | 0.0674 | 0.0192 | 0.1721 | 0.1047 | 0.0482 | 0.0764 | -0.0565 | 0.03467 | 0.23514 |
| 7(H) | 0.0513 | -0.0109 | 0.093 | 0.0416 | 0.0622 | 0.0519 | 0.0206 | 0.04478 | 0.09353 |
| 8(H) | 0.0508 | -0.009 | 0.09 | 0.0393 | 0.0597 | 0.0495 | 0.0205 | 0.043 | 0.08817 |
| 9(H) | 0.0508 | -0.0091 | 0.09 | 0.0393 | 0.0599 | 0.0496 | 0.0206 | 0.04309 | 0.08817 |
| 10(H) | 0.0513 | -0.0107 | 0.093 | 0.0417 | 0.0621 | 0.0519 | 0.0204 | 0.04468 | 0.09354 |
| 11(O) | -0.2221 | -0.2606 | -0.1357 | 0.0864 | 0.0385 | 0.0625 | -0.0479 | 0.02774 | 0.1941 |
| 12(H) | 0.1811 | 0.1519 | 0.2271 | 0.046 | 0.0292 | 0.0376 | -0.0167 | 0.02103 | 0.10321 |
| 13(Cl) | -0.0601 | -0.1665 | 0.1852 | **0.2453** | 0.1064 | 0.1759 | **-0.1389** | 0.0766 | **0.55087** |

Table S11 | Hirshfeld charges, condensed Fukui functions, condensed dual descriptors and local electrophilicity/nucleophilicity index of 2CP.

| Atom | q(N) | q(N+1) | q(N-1) | f^-^ | f^+^ | f^0^ | CDD | Electrophilicity | Nucleophilicity |
| --- | --- | --- | --- | --- | --- | --- | --- | --- | --- |
| 1(C) | -0.0487 | -0.1761 | 0.0187 | 0.0674 | 0.1274 | 0.0974 | 0.06 | 0.0861 | 0.16831 |
| 2(C) | -0.0345 | -0.1539 | 0.0384 | 0.0729 | 0.1193 | 0.0961 | 0.0464 | 0.08062 | 0.18212 |
| 3(C) | -0.0482 | -0.1062 | 0.0795 | 0.1277 | 0.058 | 0.0929 | -0.0697 | 0.03918 | 0.31888 |
| 4(C) | -0.0454 | -0.1782 | 0.0058 | 0.0511 | 0.1328 | 0.092 | 0.0817 | 0.08972 | 0.12764 |
| 5(C) | -0.0133 | -0.1031 | 0.0559 | 0.0693 | 0.0897 | 0.0795 | 0.0205 | 0.06063 | 0.17292 |
| 6(C) | 0.0684 | 0.0239 | 0.1581 | 0.0897 | 0.0446 | 0.0671 | -0.0451 | 0.0301 | 0.22392 |
| 7(H) | 0.0543 | -0.0079 | 0.0957 | 0.0414 | 0.0622 | 0.0518 | 0.0208 | 0.042 | 0.10337 |
| 8(H) | 0.0491 | -0.0122 | 0.093 | 0.0439 | 0.0613 | 0.0526 | 0.0174 | 0.04141 | 0.10964 |
| 9(H) | 0.0513 | -0.0127 | 0.0896 | 0.0383 | 0.064 | 0.0512 | 0.0257 | 0.04325 | 0.09567 |
| 10(O) | -0.1939 | -0.2378 | -0.0521 | 0.1418 | 0.0439 | 0.0928 | -0.0979 | 0.02965 | 0.35393 |
| 11(H) | 0.1553 | 0.129 | 0.1986 | 0.0433 | 0.0264 | 0.0348 | -0.0169 | 0.01782 | 0.10814 |
| 12(H) | 0.0474 | 0.0038 | 0.1001 | 0.0527 | 0.0437 | 0.0482 | -0.009 | 0.0295 | 0.13154 |
| 13(Cl) | -0.0417 | -0.1684 | 0.1187 | **0.1604** | 0.1267 | 0.1436 | **-0.0337** | 0.08561 | **0.40047** |

Table S12 | Hirshfeld charges, condensed Fukui functions, condensed dual descriptors and local electrophilicity/nucleophilicity index of 2,4-DCP.

| Atom | q(N) | q(N+1) | q(N-1) | f^-^ | f^+^ | f^0^ | CDD | Electrophilicity | Nucleophilicity |
| --- | --- | --- | --- | --- | --- | --- | --- | --- | --- |
| 1(C) | -0.0403 | -0.1668 | 0.0216 | 0.062 | 0.1265 | 0.0942 | 0.0645 | 0.10234 | 0.14736 |
| 2(C) | -0.0397 | -0.1506 | 0.0213 | 0.0611 | 0.1109 | 0.086 | 0.0499 | 0.08972 | 0.14515 |
| 3(C) | 0.009 | -0.0295 | 0.0915 | 0.0825 | 0.0384 | 0.0605 | -0.0441 | 0.0311 | 0.19619 |
| 4(C) | -0.0515 | -0.1774 | -0.0057 | 0.0458 | 0.1259 | 0.0859 | 0.0801 | 0.10186 | 0.109 |
| 5(C) | -0.0077 | -0.0968 | 0.0478 | 0.0555 | 0.0891 | 0.0723 | 0.0336 | 0.07208 | 0.13201 |
| 6(C) | 0.0699 | 0.028 | 0.1522 | 0.0823 | 0.0419 | 0.0621 | -0.0404 | 0.0339 | 0.1957 |
| 7(H) | 0.0601 | 0.0002 | 0.0979 | 0.0379 | 0.0599 | 0.0489 | 0.022 | 0.04843 | 0.09004 |
| 8(H) | 0.0548 | -0.0005 | 0.0918 | 0.037 | 0.0553 | 0.0461 | 0.0183 | 0.04471 | 0.08798 |
| 9(H) | 0.0562 | -0.0026 | 0.089 | 0.0328 | 0.0588 | 0.0458 | 0.026 | 0.04759 | 0.07802 |
| 10(O) | -0.1897 | -0.2319 | -0.0645 | 0.1252 | 0.0421 | 0.0837 | -0.083 | 0.03409 | 0.29761 |
| 11(H) | 0.1583 | 0.1328 | 0.1975 | 0.0392 | 0.0255 | 0.0323 | -0.0137 | 0.0206 | 0.09324 |
| 12(Cl) | -0.0279 | -0.1516 | 0.1056 | 0.1334 | 0.1237 | 0.1286 | -0.0097 | 0.1001 | 0.31722 |
| 13(Cl) | -0.0512 | -0.153 | 0.154 | **0.2052** | 0.1018 | 0.1535 | **-0.1034** | 0.08235 | **0.48785** |

Table S13 | Hirshfeld charges, condensed Fukui functions, condensed dual descriptors and local electrophilicity/nucleophilicity index of 2,4,6-TCP.

| Atom | q(N) | q(N+1) | q(N-1) | f^-^ | f^+^ | f^0^ | CDD | Electrophilicity | Nucleophilicity |
| --- | --- | --- | --- | --- | --- | --- | --- | --- | --- |
| 1(C) | -0.0039 | -0.0936 | 0.0372 | 0.0411 | 0.0897 | 0.0654 | 0.0486 | 0.0849 | 0.08831 |
| 2(C) | -0.0517 | -0.1683 | 0.0045 | 0.0562 | 0.1166 | 0.0864 | 0.0604 | 0.11037 | 0.12081 |
| 3(C) | 0.0134 | -0.022 | 0.0872 | 0.0738 | 0.0354 | 0.0546 | -0.0384 | 0.03349 | 0.15861 |
| 4(C) | -0.0493 | -0.1603 | -0.0115 | 0.0378 | 0.111 | 0.0744 | 0.0732 | 0.10508 | 0.08124 |
| 5(C) | 0.0099 | -0.0841 | 0.0604 | 0.0505 | 0.094 | 0.0723 | 0.0436 | 0.08905 | 0.10848 |
| 6(C) | 0.0621 | 0.0268 | 0.1414 | 0.0793 | 0.0353 | 0.0573 | -0.044 | 0.03344 | 0.17043 |
| 7(H) | 0.0587 | 0.0037 | 0.0919 | 0.0332 | 0.055 | 0.0441 | 0.0217 | 0.05205 | 0.07142 |
| 8(H) | 0.0578 | 0.0046 | 0.0878 | 0.03 | 0.0532 | 0.0416 | 0.0232 | 0.05038 | 0.06454 |
| 9(O) | -0.1818 | -0.2206 | -0.0668 | 0.115 | 0.0389 | 0.0769 | -0.0761 | 0.03679 | 0.24712 |
| 10(H) | 0.1619 | 0.1383 | 0.1991 | 0.0372 | 0.0236 | 0.0304 | -0.0136 | 0.02236 | 0.07999 |
| 11(Cl) | -0.0221 | -0.1521 | 0.1243 | 0.1463 | 0.13 | 0.1382 | -0.0164 | 0.12307 | 0.31456 |
| 12(Cl) | -0.0392 | -0.1361 | 0.1488 | **0.188** | 0.0968 | 0.1424 | **-0.0912** | 0.0917 | **0.40416** |
| 13(Cl) | -0.0159 | -0.1365 | 0.0957 | 0.1116 | 0.1207 | 0.1161 | 0.0091 | 0.11426 | 0.23982 |

Table S14 | Physicochemical characteristics of the coal chemical wastewater.

| Parameter | COD | TOC | pH (unitless) | TN | Cl^-^ | SO_4_^2-^ |
| --- | --- | --- | --- | --- | --- | --- |
| Concentration (mg/L) | 30.5 | 13.82 | 6.88 | 2.17 | 13.28 | 13.29 |

Table S15 | The concentration of metal ion in the outlet streams solution of continuous-flow reactor system. Dosage: [4CP]_0_: 0.1 mM, PI: 1 mM/L.

| Bed volume | Pt (mg/L) | Pb (mg/L) | Ni (mg/L) | Bi (mg/L) | Co (mg/L) |
| --- | --- | --- | --- | --- | --- |
| 318.3099 | -0.0010 | -0.002 | 0.02 | 0.00 | 0.02 |
| 1432.395 | -0.0010 | -0.002 | -0.01 | 0.00 | 0.00 |
| 1962.911 | -0.0010 | -0.003 | -0.01 | 0.00 | 0.00 |
| 2387.324 | 0.0000 | -0.002 | 0.01 | 0.00 | 0.01 |
| 2652.582 | -0.0010 | -0.002 | 0.01 | 0.00 | 0.01 |
| 3023.944 | -0.0020 | -0.002 | -0.01 | 0.00 | 0.01 |
| 3183.099 | -0.0020 | -0.002 | -0.01 | 0.00 | 0.00 |
| 3713.615 | -0.0010 | -0.002 | -0.02 | 0.00 | 0.00 |
| 4244.132 | -0.0010 | -0.002 | -0.03 | 0.00 | 0.00 |
| 4668.545 | -0.0010 | -0.002 | -0.03 | 0.00 | 0.00 |
| 5199.062 | -0.0010 | -0.002 | -0.05 | 0.00 | 0.00 |
| 5729.578 | -0.0020 | -0.003 | -0.03 | 0.00 | 0.00 |
| 6207.043 | -0.0010 | -0.002 | -0.05 | 0.00 | 0.00 |
| 6578.404 | -0.0010 | -0.002 | -0.04 | 0.00 | 0.00 |
|  |  |  |  |  |  |

**Supporting References**

1. Lu, T. & Chen, F. Multiwfn: a multifunctional wavefunction analyzer. J. Comput. Chem. **33**, 580–592 (2012).

2. Lu, T. A comprehensive electron wavefunction analysis toolbox for chemists, multiwfn. J. Chem. Phys. **161**, 82503 (2024).

3. Bokare, A. D. & Choi, W. Singlet-oxygen generation in alkaline periodate solution. Environ. Sci. Technol. **49**, 14392–14400 (2015).

4. Chadi, N. E., Merouani, S., Hamdaoui, O., Bouhelassa, M. & Ashokkumar, M. H_2_O_2_/periodate (IO_4_^−^): a novel advanced oxidation technology for the degradation of refractory organic pollutants. Environ. Sci. Water Res. Technol. **5**, 1113–1123 (2019).

5. Kim, Y. et al. Revisiting the Oxidizing Capacity of the Periodate–H_2_O_2_ Mixture: Identification of the Primary Oxidants and Their Formation Mechanisms. Environ. Sci. Technol. **56**, 5763–5774 (2022).

6. Chen, T. et al. Understanding the Importance of Periodate Species in the pH-Dependent Degradation of Organic Contaminants in the H_2_O_2_ /Periodate Process. Environ. Sci. Technol. **56**, 10372–10380 (2022).

7. Liu, J.-Y. et al. Direct Electron Transfer-Driven Nontoxic Oligomeric Deposition of Sulfonamide Antibiotics onto Carbon Materials for In Situ Water Remediation. Environ. Sci. Technol. **58**, 12155–12166 (2024).

8. Wu, Y., Tan, X., Zhao, J. & Ma, J. α-Fe_2_O_3_ mediated periodate activation for selective degradation of phenolic compounds via electron transfer pathway under visible irradiation. J. Hazard. Mater. **454**, 131506 (2023).

9. Ling, C. et al. Sulfide-modified zero-valent iron activated periodate for sulfadiazine removal: performance and dominant routine of reactive species production. Water Res. **220**, 118676 (2022).

10. Long, Y. et al. Atomically Dispersed Cobalt Sites on Graphene as Efficient Periodate Activators for Selective Organic Pollutant Degradation. Environ. Sci. Technol. **55**, 5357–5370 (2021).

11. He, L. et al. Fe, N-doped carbonaceous catalyst activating periodate for micropollutant removal: significant role of electron transfer. Appl. Catal., B **303**, 120880 (2022).

12. Tang, Q. et al. Electron transfer mediated activation of periodate by contaminants to generate ^1^O_2_ by charge-confined single-atom catalyst. Nat. Commun. **15**, 9549 (2024).

13. He, L. et al. Sludge biochar as an electron shuttle between periodate and sulfamethoxazole: the dominant role of ball mill-loaded Mn_2_O_3_. Sep. Purif. Technol. **314**, 123627 (2023).

14. Luo, K. et al. Activation of periodate by N-doped iron-based porous carbon for degradation of sulfisoxazole: significance of catalyst-mediated electron transfer mechanism. J. Hazard. Mater. **457**, 131790 (2023).

15. Dou, J. et al. Neglected but Efficient Electron Utilization Driven by Biochar-Coactivated Phenols and Peroxydisulfate: Polyphenol Accumulation Rather than Mineralization. Environ. Sci. Technol. **57**, 5703–5713 (2023).

16. Zhang, Y.-J. et al. Distinguishing homogeneous advanced oxidation processes in bulk water from heterogeneous surface reactions in organic oxidation. Proc. Natl. Acad. Sci. **120**, e2302407120 (2023).

17. Zhang, Y.-J. et al. Simultaneous nanocatalytic surface activation of pollutants and oxidants for highly efficient water decontamination. Nat. Commun. **13**, 3005 (2022).
